# Accelerating public sector rice breeding with high-density KASP markers derived from whole genome sequencing of *indica* rice

**DOI:** 10.1007/s11032-018-0777-2

**Published:** 2018-03-07

**Authors:** Katherine A. Steele, Mark J. Quinton-Tulloch, Resham B. Amgai, Rajeev Dhakal, Shambhu P. Khatiwada, Darshna Vyas, Martin Heine, John R. Witcombe

**Affiliations:** 1 School of the Environment, Natural Resources and Geography, SENRGY, Bangor University, Bangor, Gwynedd LL57 2UW UK; 2 Biotechnology Division, Nepal Agricultural Research Council, PO Box No. 1135, Kathmandu, Nepal; 3 Anamolbiu Private Ltd., P.O. Box 28, Jagritichok, Bharatpur-11, Chitwan, Nepal; 4 Present Address: LI-BIRD, PO Box 324, Gairapatan, Kaski, Pokhara, Nepal; 5 LGC Genomics, Units 1 & 2, Trident Industrial Estate, Pindar Road, Hoddesdon, Herts EN11 0WZ UK; 6 LGC Genomics, TGS Haus 8, Ostendstr. 25, 12459 Berlin, Germany; 7 Present Address: NuGEN Technologies Inc., 201 Industrial Road, Suite 310, San Carlos, CA 94070 USA

**Keywords:** Bacterial blight, Genomic selection (GS), Kompetitive allele-specific PCR (KASP), Marker-assisted selection (MAS), Next-generation sequencing (NGS), Physical mapping, Rice blast, Single-nucleotide polymorphism (SNP), Allele mining software

## Abstract

Few public sector rice breeders have the capacity to use NGS-derived markers in their breeding programmes despite rapidly expanding repositories of rice genome sequence data. They rely on > 18,000 mapped microsatellites (SSRs) for marker-assisted selection (MAS) using gel analysis. Lack of knowledge about target SNP and InDel variant loci has hampered the uptake by many breeders of Kompetitive allele-specific PCR (KASP), a proprietary technology of LGC genomics that can distinguish alleles at variant loci. KASP is a cost-effective single-step genotyping technology, cheaper than SSRs and more flexible than genotyping by sequencing (GBS) or array-based genotyping when used in selection programmes. Before this study, there were 2015 rice KASP marker loci in the public domain, mainly identified by array-based screening, leaving large proportions of the rice genome with no KASP coverage. Here we have addressed the urgent need for a wide choice of appropriate rice KASP assays and demonstrated that NGS can detect many more KASP to give full genome coverage. Through re-sequencing of nine *indica* rice breeding lines or released varieties, this study has identified 2.5 million variant sites. Stringent filtering of variants generated 1.3 million potential KASP assay designs, including 92,500 potential functional markers. This strategy delivers a 650-fold increase in potential selectable KASP markers at a density of 3.1 per 1 kb in the *indica* crosses analysed and 377,178 polymorphic KASP design sites on average per cross. This knowledge is available to breeders and has been utilised to improve the efficiency of public sector breeding in Nepal, enabling identification of polymorphic KASP at any region or quantitative trait loci in relevant crosses. Validation of 39 new KASP was carried out by genotyping progeny from a range of crosses to show that they detected segregating alleles. The new KASP have replaced SSRs to aid trait selection during marker-assisted backcrossing in these crosses, where target traits include rice blast and BLB resistance loci. Furthermore, we provide the software for plant breeders to generate KASP designs from their own datasets.

**Electronic supplementary material:**

The online version of this article (10.1007/s11032-018-0777-2) contains supplementary material, which is available to authorized users.

## Introduction

Cost is a major factor that determines whether or not marker-assisted selection (MAS) is a viable breeding method for national programmes and smaller breeders. Despite advantages such as improved reliability, MAS will rarely be used if it is more expensive than phenotyping. Reducing the costs of markers increases the frequency of cases where MAS is more cost-effective than phenotyping. Kompetitive allele-specific PCR (KASP) is a cost-effective and flexible proprietary technology of LGC Genomics (Semagn et al. 2014); however, public sector rice breeders have been slow to adopt it because KASP assays have not been widely published in linkage maps to the same extent as SSRs. Where costs permit, SSRs are still the marker technology most commonly used by most public sector breeders, especially for marker-assisted rice breeding (Miah et al. 2013) because they alone provide a sufficient choice of mapped markers. Breeders can choose from over 18,000 SSRs (Narshimulu et al. 2011) while the use of KASP markers is limited by the number publically available and these offer limited options in crosses between *indica* lines.

Prior to this study, 2015 KASP assays were made publically available for rice (Pariasca-Tanaka et al. 2015) that were developed in rice using a array-based Illumina GoldenGate technology by the Generation Challenge Program of the Consultative Group for International Agricultural Research (CGIAR) to analyse crosses between *Oryza sativa* ssp. *indica* and *Oryza glaberrima*. The original 2015 SNPs had been identified from the OryzaSNP project (McNally et al. 2009) and Sanger sequencing. OryzaSNP used 20 genetically diverse genotypes to discover SNPs via long range PCR and re-sequencing of microarrays. To date, and to our knowledge, no large-scale SNP and InDel discovery effort has been published for rice where NGS whole genome re-sequencing was used specifically to identify potential KASP, yet there is an urgent need for large numbers of KASP markers in rice.

KASP is a single-step genotyping technology that reveals, via fluorescence resonance energy transfer (FRET), pre-identified co-codominant alleles for both SNP and InDel variations between parents and progeny in segregating crosses for MAS. KASP has the major advantage of improved cost-effectiveness because it is both cheaper and more reliable than other marker technologies, including other sequence-based markers, such as TaqMan (Patil et al. 2017). An accessible resource of genome-wide variations would facilitate KASP to be used for whole genome coverage in genomic selection (GS) which has been pioneered using and array-based technology. Array-based genotyping and NGS-based genotyping technologies (such as genotyping by sequencing) are not being taken up by public sector breeders for MAS because they lack the flexibility and ease afforded by SSRs (Yang et al. 2015). KASP offer the benefits of SSRs plus the added ability of being able to detect functional markers within target genes (Rasheed et al. 2016), and KASP are easier to use: either LGC Genomics can provide a full KASP genotyping service or the KASP reagents can be ordered from them for carrying out assays in a basic molecular laboratory. KASP technology is more rapid than SSRs, and it has scalability that makes it suitable for a wide range of experimental designs with greatly varying target loci and sample numbers (He et al. 2014). These can range from only a single marker, such as a selectable marker for a specific gene, through to several thousands of markers for applications such GS. The effectiveness of KASP has been demonstrated in plant-breeding applications, including quality control analysis of germplasm (Semagn et al. 2012; Ertiro et al. 2015), screening for candidate alleles and genotyping (Mideros et al. 2013; Pham et al. 2015), bulk segregant analysis and genetic mapping (Ramirez-Gonzalez et al. 2014; Mackay et al. 2014) and MAS (Cabral et al. 2014; Leal-Bertioli et al. 2015).

Marker-assisted breeding has been introduced in Nepal’s national programmes, mainly based on SSRs but recently incorporating existing KASP for background selection. However, few of the existing rice KASP were suitable for selection at the breeders’ targets of BLB and blast resistance genes and aroma quantitative trait loci (QTLs). Therefore, the objective of the work reported here was to identify appropriate SNPs and InDels, for this purpose, in order to facilitate the uptake of KASP for greater efficiency of rice breeding. At current rates, the KASP genotyping service is estimated to be 60% cheaper than running SSRs in-house at NARC’s laboratories in Kathmandhu, Nepal: Genotyping 475 samples with 10 assays costs $2.0 per data point with KASP (full genotyping service, including shipping costs), $3.9 with in-house KASP and $5.3 with in-house SSRs.

This study used whole genome NGS specifically to identify large numbers of SNP and InDel variations and used bioinformatics filtering of NGS reads to discover potential KASP assays throughout the rice genome. We re-sequenced nine *indica* rice lines and aligned the sequences to the *indica* reference genome to maximise the identification of applicable loci. The study provides new evidence for the effectiveness of using NGS sequence data from a limited number of lines and makes comparisons between the new potential KASP and those that were available prior to this work for density and genomic distribution throughout the rice physical map in a range of crosses.

## Materials and methods

### Plant materials and DNA extraction for NGS

Nine *indica* rice lines (Table S1) were selected for sequencing. Three (Sunaulo Sugandha, Anamol Masuli and Sugandha-1) were from a breeding programme in Nepal (Witcombe et al. 2013), and one (Khumal-4) is a widely grown mid-hill variety in Nepal. They are all being used as recurrent parents for rice breeding in Nepal. Sunaulo Sugandha and Sugandha-1 are aromatic. Four (IR64, IR71033, IR65482, IRBB60 and Loktantra) were chosen as donors of resistance to the diseases bacteria blight (caused by *Xanthomonas oryzae* pv. *oryzae*) and blast (caused by *Magnaporthe oryzae*). Seedlings were grown in a controlled environment room at Bangor University (BU) and DNA extracted at BU from the leaves of one representative seedling per variety using Qiagen DNEasy kits (Qiagen, Manchester, UK). The plants were grown to maturity and visually checked for phenotypic uniformity within each variety.

### Sequencing, read processing and read alignment

Paired-end sequencing, using the Illumina HiSeq 2000 platform, and read processing were carried out at LGC Genomics (Berlin, Germany). For bioinformatics analysis, Illumina adaptor sequences were removed and quality trimming of adaptor-clipped reads was performed, removing reads containing Ns and 3′-end trimming reads to get a minimum average Phred quality score of 20 over a window of ten bases. Reads with a final length of less than 20 bases were discarded. The sequences have been submitted to the NCBI Sequence Read Archive under BioProject accession PRJNA395505 (available at www.ncbi.nlm.nih.gov/bioproject/395505).

The reference genome sequence used was cultivar 93-11 of *Oryza sativa* ssp. *indica*. The Read Assembly version ASM465v1 of 93-11, sequenced and annotated by the Beijing Genome Institute (Yu et al. 2002; Zhao et al. 2004), was downloaded from EnsemblPlants (http://plants.ensembl.org). Sequencing reads were aligned against this reference using Bowtie2 (Langmead and Salzberg 2012). Discordant or mixed paired-read alignments were not permitted, with all other alignment parameters kept as default. Only read pairs with both reads aligning in the expected orientation were used in subsequent analyses.

### Variant calling

SAMtools (Li et al. 2009) was used to calculate genotype likelihoods and identify single nucleotide polymorphisms (SNPs) and InDels between the aligned sequencing reads and the *O. sativa* ssp. *indica* reference. SNPs or insertions with a read depth higher than 200 were filtered out (using vcfutils) due to likelihood of variable copy number repeats influencing read mapping. Also, those with a read depth of less than five were removed. Custom Perl scripts were used to identify variants between all pairwise combinations of the nine rice lines, based on the variant calls made for each variety against the *indica* reference. The positions of the variants were compared against the annotated gene and coding sequence positions to test whether they corresponded to functional mutations.

### Variant filtering for suitability as KASP markers

Variant Call Format (VCF) files generated by SAMtools (see above) were parsed using a custom Perl script (Supplementary File S1) to retrieve the flanking sequences 50 bp either side of each variation site and identify variants suitable for KASP markers following a stepwise identification process (Fig. S1). The criteria for selection were that the flanking sequences (a) did not contain any InDels, (b) contained a maximum of four ambiguous bases, (c) had a base coverage of at least five at any position and (d) had no more than four consecutive repeats of any one to five nucleotide sequences. Variants that passed this filtering were defined as potential KASP markers. The SNP positions of the potential KASP markers were used in the diversity analysis of potential KASP assays below.

### In silico analysis of diversity and marker density using the new and existing KASP markers

The sequence variants (SNPs and InDels) of each of the 1,329,325 potential KASP that passed the filtering (Fig. S1) were used to make 45 comparisons—the 36 possible pairwise comparisons between the nine re-sequenced lines and each of the lines compared with the *indica* reference genome. For the 2015 existing KASP markers based on rice SNPs that had previously been developed (Pariasca-Tanaka et al. 2015), the KASP primer sequences were aligned against the *indica* reference using BLAST (Altschul et al. 1990) to determine if the sequence reliably aligned to *indica* (those with at least 95% identity). This eliminated 205 KASP specific to *japonica*. A further 731 KASP were at sites where no polymorphism was detected between any of the nine lines and the *indica* reference. This left 1159 existing KASP markers that were used for the same 45 comparisons. The density of marker coverage was compared by finding the distribution of distances between all consecutive polymorphic markers for both potential and existing KASP for all 45 pairwise comparisons.

### Plant materials and DNA extraction for genotyping in segregating populations

For KASP genotyping, plants representing the nine sequenced parental lines and progeny lines (at F_1_ and BC_1_) derived from 15 crosses between pairs of parents were grown in the field or polyhouse in Nepal, in October 2015 and October 2016. All plants were from the marker-assisted breeding programmes of either Anamolbiu or NARC, and parental lines were used as controls for MAS. Leaf samples were collected from each plant and put into separate wells in 10 Plant Sample Collection Kits (Supplied by LGC Genomics) and the 10 plates containing samples were delivered to LGC Genomics (Hoddesdon, Herts., UK) for DNA extraction and KASP genotyping (full service). The first three plates were screened in the first round (69 KASP including 21 new ones) and third round (with a further 5 new KASP). Five plates were screened in the second round with 86 KASP. Two plates containing only BC_1_ material were screened with 40 KASP (39 new) in the fourth round.

### Development and validation of new KASP assays

The SNPs or InDels selected for validation in this study were located either near to/within target resistant gene alleles (for BLB or blast) or to known fragrance QTLs, or they were useful as background markers in regions where no existing KASP were suitable. They included 35 variants that passed the filtering criteria and 11 variants that did not pass. All 46 new KASP assays gave in silico validated primers in LGC’s Kranken Software, and KASP primers were produced by LGC and used in their standard protocol for KASP validation. Here, we define validation as where the KASP assay was successfully used for genotyping in at least one cross. In total, four separate rounds of genotyping were carried out on different sets of segregating lines, each round having a different combination of new and existing KASP assays.

Marker-level, cross-level and assay-level validations of the KASP assays were carried out using bioinformatics on genotype results from all four rounds of genotyping. KASP markers were considered to be validated if they successfully genotyped any of the tested progeny lines and identified both predicted parental alleles. Cross-level validation assessed whether a marker could be validated at the marker level using only progeny lines originating from a specific pair of parental lines. Assay-level validation tested whether or not each individual KASP assay had produced genotyping results. Genotyping results from within replicates of the same parental lines were not used for validation as they would be expected to be homozygous for the tested alleles, and thus, the genotyping results could not be used to validate successful binding of both of the KASP allele-specific primers.

### Identification and subsequent filtering of ‘background’ markers

From the existing 1159 KASP that reliably aligned to the *indica* reference genome, we identified those that were polymorphic in silico in at least three of the bi-parental crosses used for this study. Of these, 75 were selected as background markers for genotyping because they were distributed in genomic regions required for recurrent parent selection. Of the 75 existing KASP, 48 met our filtering criteria (Fig. S1) for selecting variants appropriate for marker generation. These existing KASP were used for genotyping in parental and progeny lines by LGC Genomics (Hoddesdon, Herts., UK).

## Results

### Sequencing read alignment and identification of variants

More sequencing reads of all of the nine re-sequenced rice lines aligned in the expected orientation to the *indica* reference (mean of 92.1% ± 0.96) than to the *japonica* reference (mean of 88% ± 0.69). Mean *indica* genome coverage was 89% with a mean sequencing depth of 59 for the nine lines (Table S2). We identified variations between the *indica* reference and at least one of the nine lines at 2,561,351 unique sites. For over half (56.5%) of these sites, two or more lines were polymorphic against the reference genome and for 3.4% of sites all nine were polymorphic against the reference, whereas more than one million variant sites were found in only a single line (Fig. S2). There was an average of 0.96 million homozygous variations (SNPs and InDels) between each of the nine rice lines compared with the *indica* reference variety 93-11 (Fig. S3). IR71033 was the most similar line to the reference (0.78 million variations) and Sunaulo Sugandha the least similar (1.1 million variations).

### Identification of potential KASP markers and functional markers

To identify KASP markers that would be informative for crosses between the nine lines and the *indica* reference, in silico filtering of the 2,561,351 variation sites was carried out, based on the composition of their flanking sequences (Fig. S1). The KASP marker sequences were determined for the 1,329,325 sites that passed the filtering criteria, i.e., a conversion rate of 51.9% of the total variation sites.

For each of the nine lines, those variations that were suitable for KASP markers were categorised according to the nature of the polymorphism against the *indica* reference (Table 1), as determined according to the annotated gene and coding sequence positions. The majority of potential KASP were situated in noncoding portions of the genome, with 78% located in intergenic regions and 11% in introns. Of the remaining 11% of variations located in the exons, 68% are predicted to result in functional differences due to changes in the amino acids encoded.

**Table 1:** Categorisation of variations suitable as KASP markers identified between each of the nine sequenced rice lines and the indica reference genotype

Line	SNPs						InDels				
	Intergenic	Intron	Exon				Intergenic	Intron	Exon		
			Nonsynonymous^a^	Synonymous	Unknown^b^	Ratio of Nonsyn/syn			Frameshift^a^	Inframe^a^	Ratio of FS/non-FS
IR64	276,103	36,791	25,280	11,946	1174	2.12	28,831	5632	1808	782	2.31
IR71033	214,507	29,360	20,848	9703	995	2.15	26,527	5007	1744	678	2.57
IR65482	316,846	41,673	29,910	14,158	1554	2.11	34,287	6527	2000	949	2.11
Sunulo-Sugandha	326,995	43,021	29,492	14,084	1336	2.09	32,242	6267	1868	924	2.02
Anmol-Masuli	306,934	40,889	28,462	13,469	1375	2.11	33,241	6486	1955	958	2.04
Khumal-4	260,116	34,685	23,594	11,057	1175	2.13	30,475	5803	1842	825	2.23
IRBB-60	217,103	30,887	21,796	10,245	992	2.13	27,830	5358	1796	750	2.39
Loktantra	306,922	39,801	27,752	13,149	1307	2.11	35,428	6622	2079	941	2.21
Sugandha-1	233,949	31,956	22,994	10,718	1143	2.15	29,016	5483	1842	815	2.26
Mean of nine lines	273,275 (70.4%)	36,563 (9.4%)	25,570 (6.6%)	12,059 (3.1%)	1228 (0.3%)	2.12	30,875 (8.0%)	5909 (1.5%)	1882 (0.5%)	847 (0.2%)	2.24

^a^Nonsynonymous SNPs and all InDels within exons are assumed to be functional markers

^b^SNPs within the coding regions of annotated genes were categorised as unknown if the corresponding amino acid could not be determined with certainty due to the presence of ambiguous bases

### Comparing diversity in nine *indica* lines with new KASP

This new approach of pairwise comparisons for each of the nine re-sequenced lines against each other and against the *indica* reference genome identified many more potential new KASP than previously existed for rice (Table 2). The highest marker diversity detected in the pairwise comparisons was 511,006 by the new set (IR65482 with Sunaulo Sugandha) compared with 522 by the existing set (Loktantra with Sunaulo Sugandha). The least informative number of KASP markers in the pairwise comparisons was 245,367 by the new set (IR64 with IR71033) compared with 361 by the existing set (IR64 with IR71033). A similar pattern was seen for comparisons with the *indica* reference where the average number of informative KASP markers was 388,540 for the new set and 451 for the existing set. The highest number of new markers against the reference genome was 459,229 for Sunaulo Sugandha, compared with a maximum of 496 for Loktantra with the existing markers.

**Table 2:**
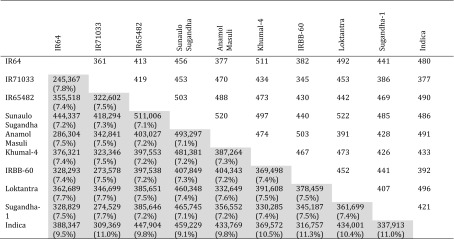
Number of informative markers for each pairwise comparison of the nine sequenced rice lines and the *indica* reference genotype

Numbers in the lower-left diagonal (shaded) correspond to counts of potential new informative KASP markers identified in this study based on SNPs, with percent of InDels shown in brackets. Numbers in the upper-right diagonal correspond to counts of informative markers from the existing set of 1890 KASP markers that could be aligned against the *indica* reference. All existing informative markers are SNPs

**Fig. 1:**
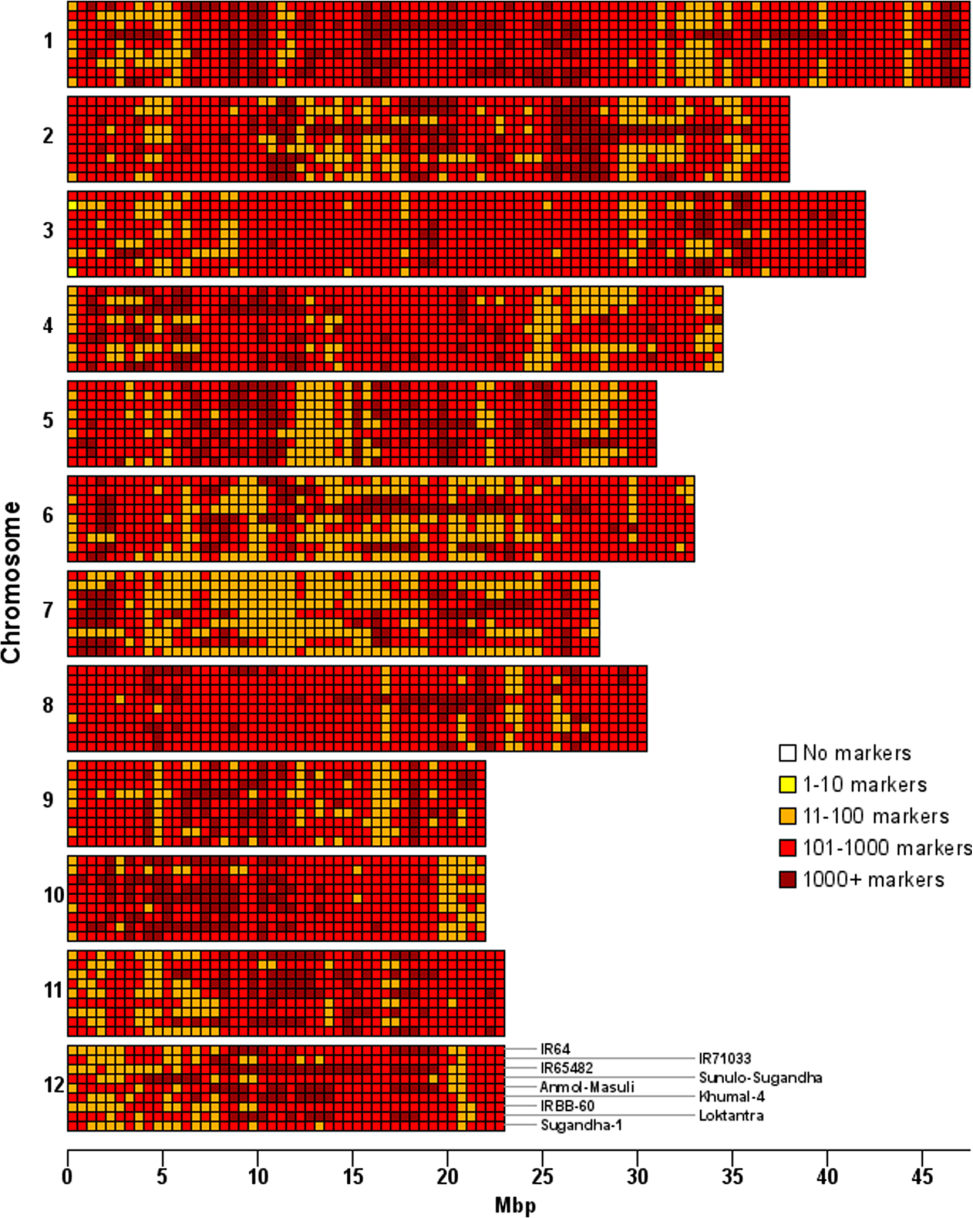
Distribution of potential new KASP markers polymorphic between each rice line and the indica reference. Rows represent the chromosomes, subdivided into the different lines in the order indicated on chromosome 12 (from top to bottom: IR64, IR71033, IR65482, Sunaulo Sugandha, Anamol Masuli, Khumal-4, IRBB-60, Loktantra, Sugandha-1) and columns the physical position. Each cell represents an interval of 0.5 Mbp

The new KASP markers were distributed throughout the entire genome with high levels of marker density (Fig. 1). In a great majority of cases (86.9%), the distance between consecutive informative markers was less than 1 kb with a median distance of 127 bp in all pairwise combinations. Chromosomal distribution plots of markers informative for each pairwise combination of the sequenced lines show very few regions with no markers (Fig. S4).

### Comparing diversity in nine *indica* lines with existing KASP

**Fig. 2:**
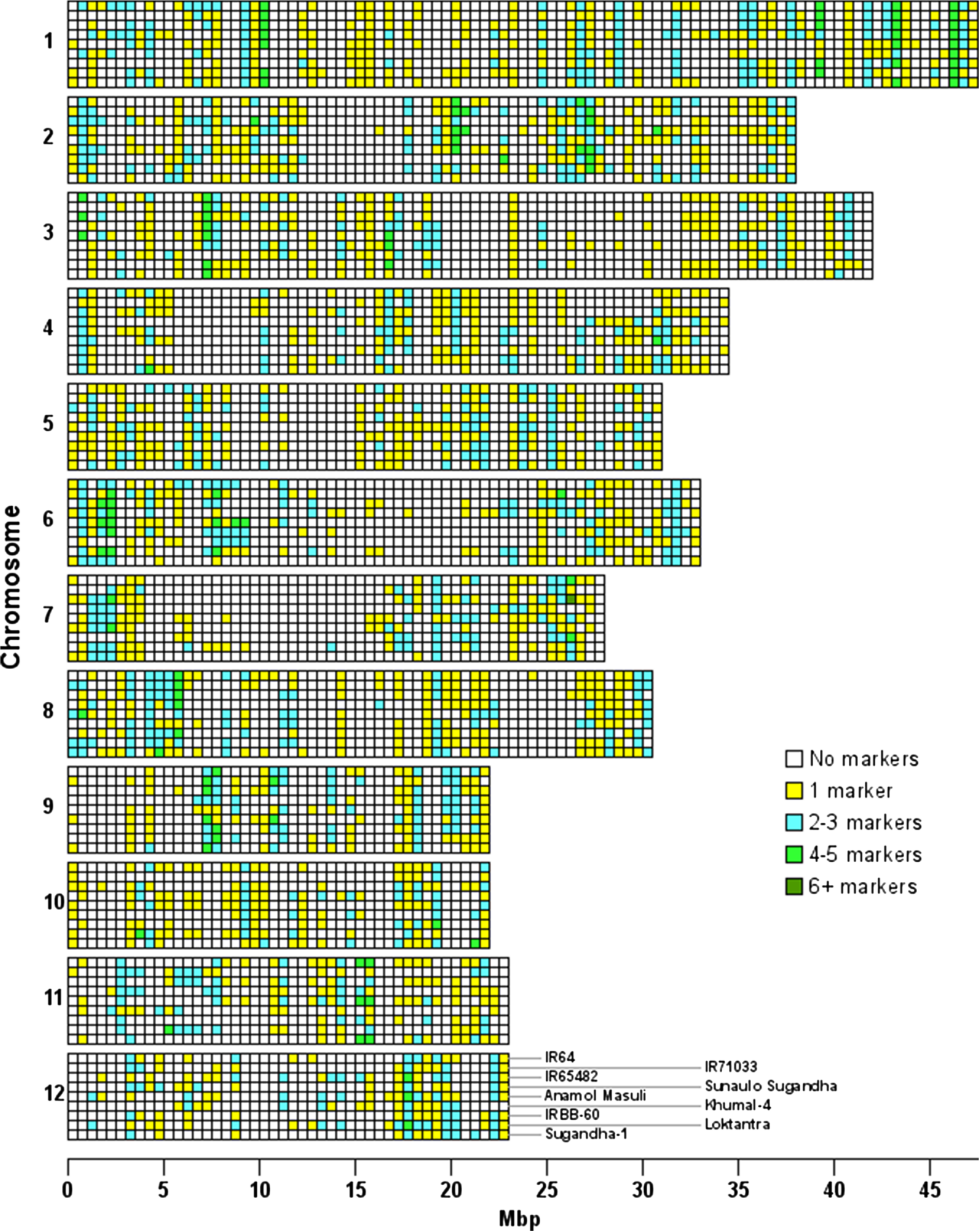
Distribution of previously existing rice KASP markers polymorphic between each rice line and the indica reference genome. Rows represent the chromosomes, subdivided into the different lines in the order indicated on chromosome 12 (from top to bottom: IR64, IR71033, IR65482, Sunaulo Sugandha, Anamol Masuli, Khumal-4, IRBB-60, Loktantra, Sugandha-1) and columns the physical position. Each cell represents an interval of 0.5 Mbp

Of the 1890 existing KASP markers that could be aligned against the *indica* reference, 1159 (61%) were polymorphic between at least one of the sequenced lines and the *indica* reference genome. However, they were not evenly distributed throughout the genome nor across all lines (Fig. 2). In pairwise comparisons between the lines, there were between 345 and 520 informative polymorphic markers for each cross combination (Table 2; Fig. S5). There were some areas of the genome that had polymorphisms in all of the crosses (e.g. between 0.5 and 10 Mbp on chromosome 6), but many regions had polymorphisms only in specific pairs of crosses. There were also many regions lacking any polymorphisms (e.g. on chromosome 7 between 9 and 16 Mbp there is only one region with any polymorphic markers and it is only in crosses with Loktantra). Consecutive informative existing KASP markers were closer than 1 kb in only 1.1% of cases. The median distance between markers is 353 kb across all pairwise combinations of lines, this is a median gap size over 2700 times longer than that found for the new markers (Fig. S6; Tables S3 and S4).

The positions of the 1159 markers that aligned to the indica reference and corresponded to polymorphic sites in our lines were compared with the positions of the new KASP markers. Matches were found for 727 (62.7%) of the existing markers, with new markers not being identified at the other genomic positions due to the filtering criteria applied by the marker detection algorithm (Fig. S1). The filtering method excluded 37% (432 of 1159) existing KASP markers because they had InDels or repeats of five or more bases in their flanking regions.

### KASP validation for use in genotyping

KASP genotyping was carried out on F_1_ and BC_1_ progeny of 15 crosses between pairs of the nine re-sequenced lines. For the purposes of KASP validation, genotyped progeny of different generations was grouped according to the parental lines initially crossed, with a KASP assay being considered validated for a particular group if genotyping was successful in showing segregation of alleles for one or more progeny lines from any generation of the cross. Eighty-three markers (35 new and 48 existing KASP) that passed our filtering criteria (Fig. S1) were tested on at least one cross, with a total of 412 unique marker-cross combinations. Successful genotyping results were obtained for 78 (94.0%) of these markers including 30 of the new markers, with 394 of 412 (95.6%) marker-cross combinations being successful (Tables S5 and S6).

Genotyping was also carried out with 38 markers (11 new and 27 existing KASP) that did not meet our filtering criteria; the 11 new markers were designed manually through visualisation of the aligned sequencing reads at sequences for target traits. Thirty-one (81.6%) of these markers gave genotyping results in at least one of the progeny tested, including nine of the new markers. Two hundred and thirty-two marker cross combinations were tested, with 201 (86.6%) being successful (Tables S5 and S6).

Parental lines were genotyped with the KASP markers as controls, and the results not only confirmed the presence of the predicted alleles in the parents but also revealed within-line genetic variation for some of the parents at some loci (data not shown). Expected allelic ratios were detected in segregating progeny for all successfully genotyped crosses (data not shown), and the results informed selection of donor alleles and recurrent (background) alleles for 70 existing KASP and 39 newly validated KASP (Table 3; Table S7), of which 30 were discovered from filtering and 9 identified by manual design.

**Table 3:**
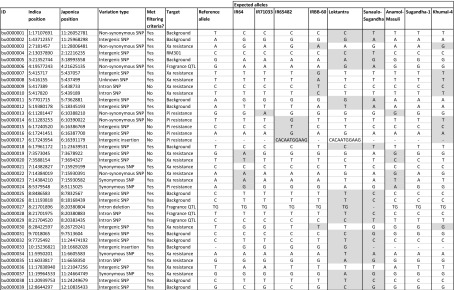
New validated KASP assays available from LGC genomics (for sequences see Table S7)

Positions are based on the *indica* ASM4565v1 and *japonica* IRGSP-1.0 reference genomes. Linkage analysis is underway to assign linkage to traits in relevant crosses; preliminary data for IR64 × Jumli Marshi shows that bu0000024 is associated with field resistance to BLB locus Pi33 (*χ*^2^ = 29.6, *P* < 0.01). Shading shows an example of a cross in which the KASP is being used for selection

## Discussion

SNPs provide the highest genome-wide density of genetic variants and occur in both coding and noncoding genomic regions. Due to their bi-allelic nature, not all SNPs and InDels will be polymorphic for all cross combinations. We showed that, for the existing 2015 rice KASP markers (all SNPs) published by Pariasca-Tanaka et al. 2015, in all cross combinations, there were very large gaps between markers across the rice genome (Fig. 2). Only 1890 existing KASP were applicable to *indica*, and the number that were informative between any pair of nine *indica* lines studied here varied from as few as 361 to, at most, 522. It is unsurprising that the existing set is insufficient to meet all rice-breeding challenges because, apart from being less numerous than available SSRs, they were derived from chip-based technologies based on SNPs nominated by the rice community to address particular breeding targets. Hence, a much higher density of SNPs or InDel variants is needed in order to identify suitable markers for selection in a broader range of specific crosses.

Thousands of SNPs have previously been employed in array-based platforms such as those used in the Illumina Bead Array and the Affymetrix GeneChip (Thomson 2014). However, unlike KASP, these fixed sets of SNPs do not meet the need of breeders that wish to assay a small number of polymorphic markers known to be linked to traits of interest in their breeding populations, and to have the opportunity to change the set of markers used in subsequent generation. Next-generation sequencing (NGS) technologies have been used to re-sequence diverse rice genomes or for genotyping in technologies such as genotyping by sequencing (GBS) (McCouch et al. 2010; Kumar et al. 2012), but most variants have only been made available on array-based platforms.

Here, NGS was used for re-sequencing nine *indica* breeding lines, chosen with no deliberate effort to select for high diversity, and it identified an average of 1.05 million SNP or InDel variants between any one of the individual rice lines and the *indica* reference genome, out of a total of 2.5 million variants across the whole set of lines (available at www.ncbi.nlm.nih.gov/bioproject/395505). By mining this data using bioinformatics filtering, we discovered hundreds of thousands of potential new KASP markers giving high resolution coverage over the entire genome (Fig. 1; Table 1). This analysis has vastly reduced the number of regions with no selectable markers (compare Figs. 1 and 2), offers breeders access to over 1.3 million informative KASP with a minimum of more than 245,000 potential markers for any paired combination of the 9 rice lines (Table 2) and has produced over 650 times more KASP marker sequences than were available in rice to date. Approximately 92,500 (7%) were located in exons and altered the amino acid sequence encoded and so could be used as functional markers (Table 1). For all pairwise comparisons between lines, over 98% of consecutive informative markers were less than 10 kb apart, with over 85% being less than 1 kb apart (Fig. S6). Some of these comparisons were between lines having common recent ancestors (Table S1), so this dataset should provide a high density of polymorphic KASP assays across the genome in almost any cross. Moreover, these estimates are conservative, as many more KASP markers would be identified if the filtering criteria were relaxed slightly to allow the detection of KASP markers in gaps at target genomic regions. Relaxing the criteria is a practical option as they were very stringent; they provided a 52% conversion rate for new markers from identified variations but excluded 37% of the 1159 existing KASP.

Early rice genome sequencing of *indica* and *japonica* revealed about one SNP per kb (Feltus et al. 2004), and the material that is subsequently selected to be re-sequenced determines the density of NGS based markers identified. Re-sequencing of 12 cultivated and wild accessions of *indica* that were chosen for diversity gave an average of 5.7 nucleotide differences per kb diversity (Xu et al. 2012). Here, we found an average of 3.1 variations per kb in *indica* lines used for breeding in Nepal. All lines were adapted to lowland or medium land and we made no attempt to include diverse lines adapted to greatly different rice ecosystems. They were simply chosen on the basis of being in current breeding programmes in Nepal and seven of the lines were either bred at IRRI or had IRRI lines in their recent ancestry. Hence, the high frequency of KASP markers (Fig. 1) we have discovered should also apply to most, or all, other *indica* material of interest to breeders. The total of 2.5 million SNPs in the nine lines compares favourably with the total of 18.9 million found in the 3000 Rice Genomes Project where the lines included were highly diverse across all the *O. sativa* cultivated groups (Li et al. 2014).

We have demonstrated how high-throughput sequencing data can be used to identify so many new KASP markers that they will be useful for many traits across many parental combinations. A set of 39 fully validated marker designs are given here (Table 3). These design sequences can be submitted directly to LGC Genomics for purchase of KASP primers through their KASP by Design (KBD) or KASP on Demand (KOD) services or for their full genotyping service. This allows breeders with no bioinformatics expertise to utilise these markers in their breeding programmes. The software provided (Supplementary File S1) can enable breeders to easily generate KASP marker designs using their own, or publicly available, NGS datasets—for any species. In addition, the sequencing reads for the nine re-sequenced lines is a valuable resource containing suitable variants for numerous breeding targets.

The work has led to suitable KASP assays for NARC and Anamolbiou (Nepal), and many more assays are being rolled out to rice breeders in India (SKUAST) and Pakistan (NIBGE) with the services of LGC Genomics. Work is currently underway, using data from the 3000 Rice Genomes Project, to generate over 20,000 KASP marker designs, of which 4000 will be fully validated, that will be applicable to a diverse range of rice varieties. These will be made available on the LGC Genomics website, allowing breeders to purchase KASP markers close to existing SSR markers or in a region of interest, without the need for any bioinformatics analysis. In the meantime, the paper authors can be contacted for details of KASP marker designs based on the nine re-sequenced lines, for any particular region of the rice genome. This increase in the number of usable KASP markers has great practical benefits to public sector plant breeders who can use the knowledge derived from this project to incorporate KASP into MAS to accelerate selection of new varieties. These KASP assays are new tools that can complement other innovations introduced to accelerate varietal adoption by farmers in developing nations (Witcombe et al. 2016) to expedite yield improvement and increase food security. By increasing the number of available KASP markers, this work is expected to remove the barriers to their adoption so they can accelerate progress in rice breeding for future generations.

## Electronic supplementary material

ESM 1 **File. S1** KASP marker design sequence generation software. (PDF 215 kb)

ESM 2 **Figure. S1** Overview of the criteria used for identification of potential KASP markers from variations identified using SAMtools. **Figure. S2** Number of variations identified at the same positions relative to the indica reference in all sequenced rice lines (maximum nine). **Figure. S3** Number of variations (homozygous SNPs, insertions and deletions) identified between each of the nine sequenced rice lines and the indica reference genome. **Figure. S4** Distribution of potential new rice KASP markers polymorphic between each rice line pair. Rows represent the chromosomes, subdivided into the different lines (ordered as indicated on chromosome 12), and columns the physical position. SubFigures show the distribution of markers informative for crosses against (a) IR64 (b) IR71033 (c) IR65482 (d) Sunaulo Sugandha (e) Anamol Masuli (f) Khumal-4 (g) IRBB-60 (h) Loktantra (i) Sugandha-1. **Figure. S5** Distribution of existing rice KASP markers polymorphic between each rice line pair. Rows represent the chromosomes, subdivided into the different lines (ordered as indicated on chromosome 12), and columns the physical position. SubFigures show the distribution of markers informative for crosses against (a) IR64 (b) IR71033 (c) IR65482 (d) Sunaulo Sugandha (e) Anamol Masuli (f) Khumal-4 (g) IRBB-60 (h) Loktantra (i) Sugandha-1. **Figure. S6** Distribution of distances between consecutive informative KASP markers, for new and existing markers, for all combined pairwise combinations of rice lines used in this study. Vertical bars represent the medians with boxes extending from the 25th to 75th percentiles. Whiskers extend from the 5th to 95th percentiles, dots represent the minimum and maximum distances. (PDF 4943 kb)

ESM 3 **Table S1** Details of nine *indica* rice cultivars and breeding lines used for whole genome NGS. **Table S2** Mapping rates, genome coverage and read depth of the nine sequenced rice lines, for all mapped reads and for uniquely-mapped reads only. Only those reads with both pairs aligning in the expected orientation were included. **Table S3** Distribution of distances (bp) between consecutive informative existing KASP markers (previously developed using chip-based technology) for all rice line pairings. **Table S4** Distribution of distances (bp) between consecutive informative potential new KASP markers (developed here using NGS data) for all rice line pairings. **Table S5** Summary of KASP assay validation results. Counts are given of the number of unique marker-cross combinations that have been validated (or otherwise), split according to whether or not the markers met our bioinformatics filtering criteria, and between new and existing markers. **Table S6** Details of marker-cross combinations tested for validation of 46 new and 75 existing KASP assays (N.B. some failed assays may be due to lack of polymorphism.) (PDF 443 kb)

ESM 4 **Table S7** Sequences for new validated KASP assays available from LGC genomics. (PDF 50 kb)
