# Supplementary material for: Accelerating public sector rice breeding with high-density KASP markers derived from whole genome sequencing of indica rice
Source: Mol Breed. 2018 Mar 7;38(4):38. doi: 10.1007/s11032-018-0777-2 (PMC5842261; doi:10.1007/s11032-018-0777-2)

Supplementary Figures

Accelerating public sector rice breeding with high-density KASP markers derived from whole genome sequencing of *indica* rice

Katherine A. Steele · Mark J. Quinton-Tulloch · Resham B. Amgai · Rajeev Dhakal · Shambhu P. Khatiwada · Darshna Vyas · Martin Heine · J. R. Witcombe

K.A.Steele (corresponding author: email k.a.steele@bangor.ac.uk, Tel 00 44 1248 388655)
M.J. Quinton-Tulloch · J.R. Witcombe
School of the Environment, Natural Resources and Geography, SENRGY, Bangor University, Bangor, Gwynedd, LL57 2UW, UK

R.B. Amgai · S.P. Khatiwada
Nepal Agricultural Research Council, Biotechnology Division, PO Box No. 1135 Kathmandu, Nepal

R. Dhakal
Anamolbiu Private Ltd., P.O. Box 28, Jagritichok, Bharatpur-11, Chitwan, Nepal. Current address: LI-BIRD, Head Office: PO Box 324, Gairapatan, Pokhara, Kaski, Nepal

D. Vyas
LGC Genomics, Units 1 & 2, Trident Industrial Estate, Pindar Road, Hoddesdon, Herts, EN11 0WZ, UK

M. Heine
LGC Genomics LGC Genomics TGS Haus 8, Ostendstr. 25, 12459 Berlin, Germany; ***Current address****: NuGEN Technologies Inc.201 Industrial Road, Suite 310 San Carlos, CA 94070, USA*

Fig. S1 Overview of the criteria used for identification of potential KASP markers from variations identified using SAMtools.

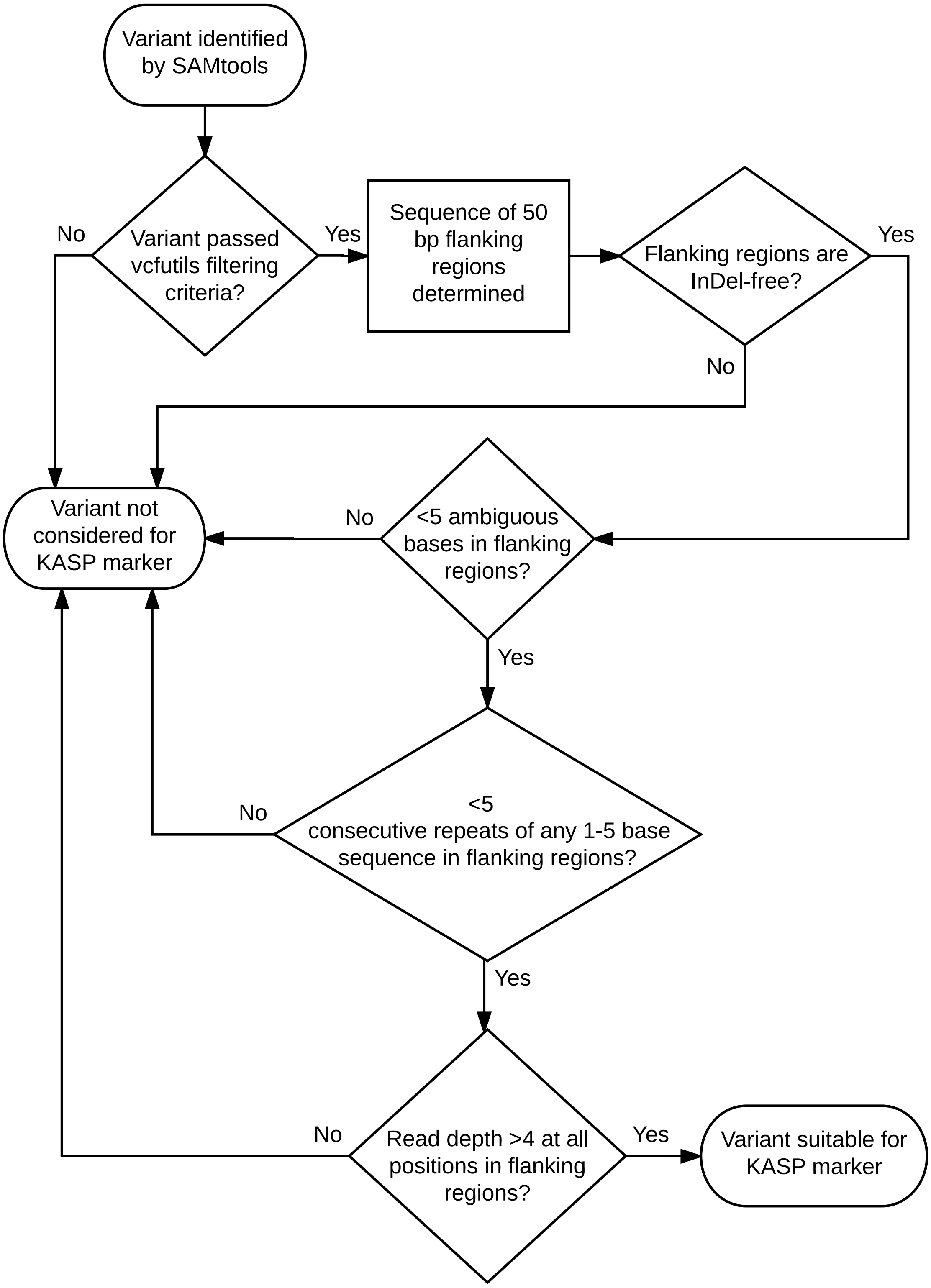

Figure. S2 Number of variations identified at the same positions relative to the indica reference in all sequenced rice lines (maximum nine).

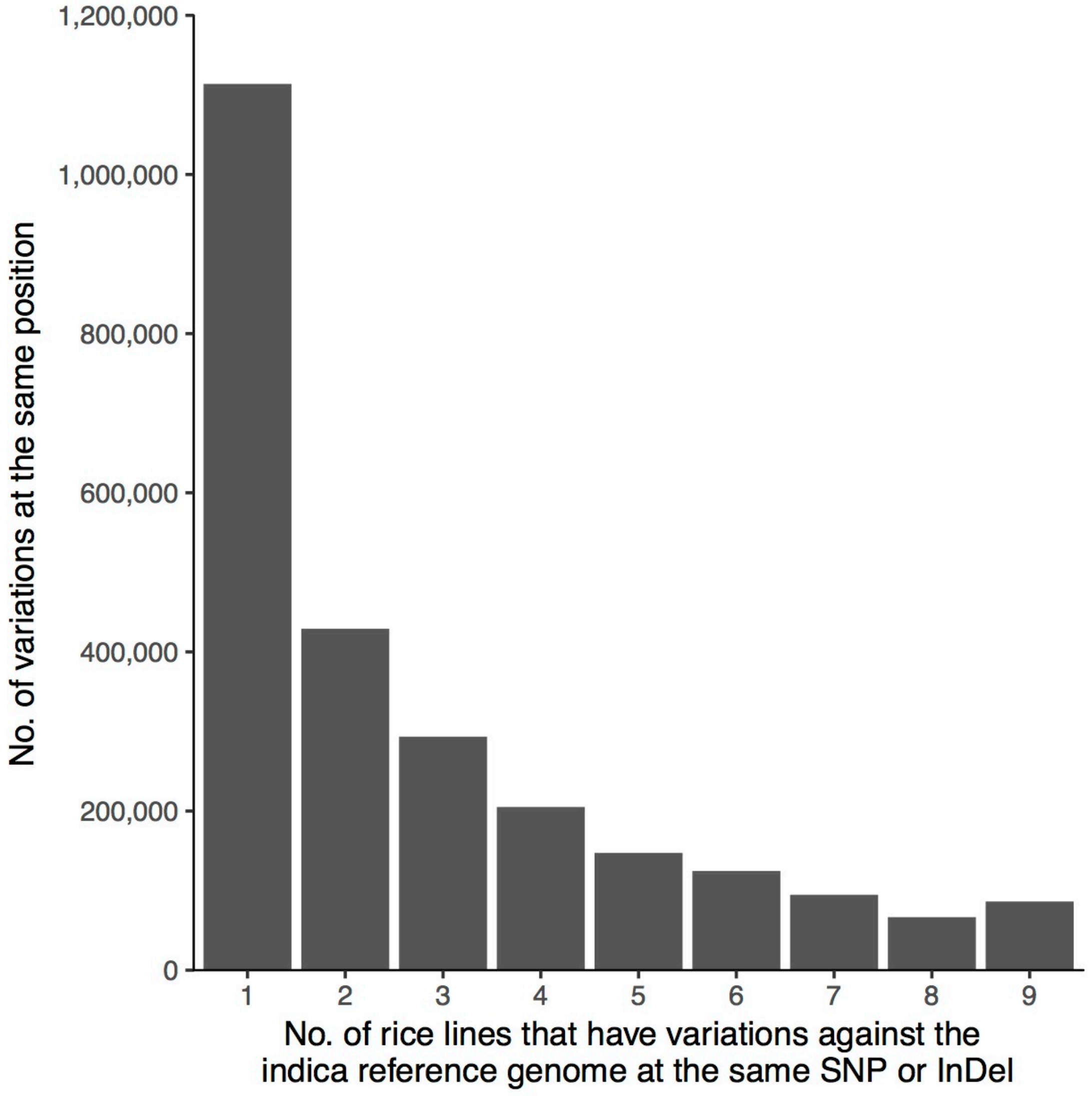

Figure. S3 Number of variations (homozygous SNPs, insertions and deletions) identified between each of the nine sequenced rice lines and the indica reference genome.

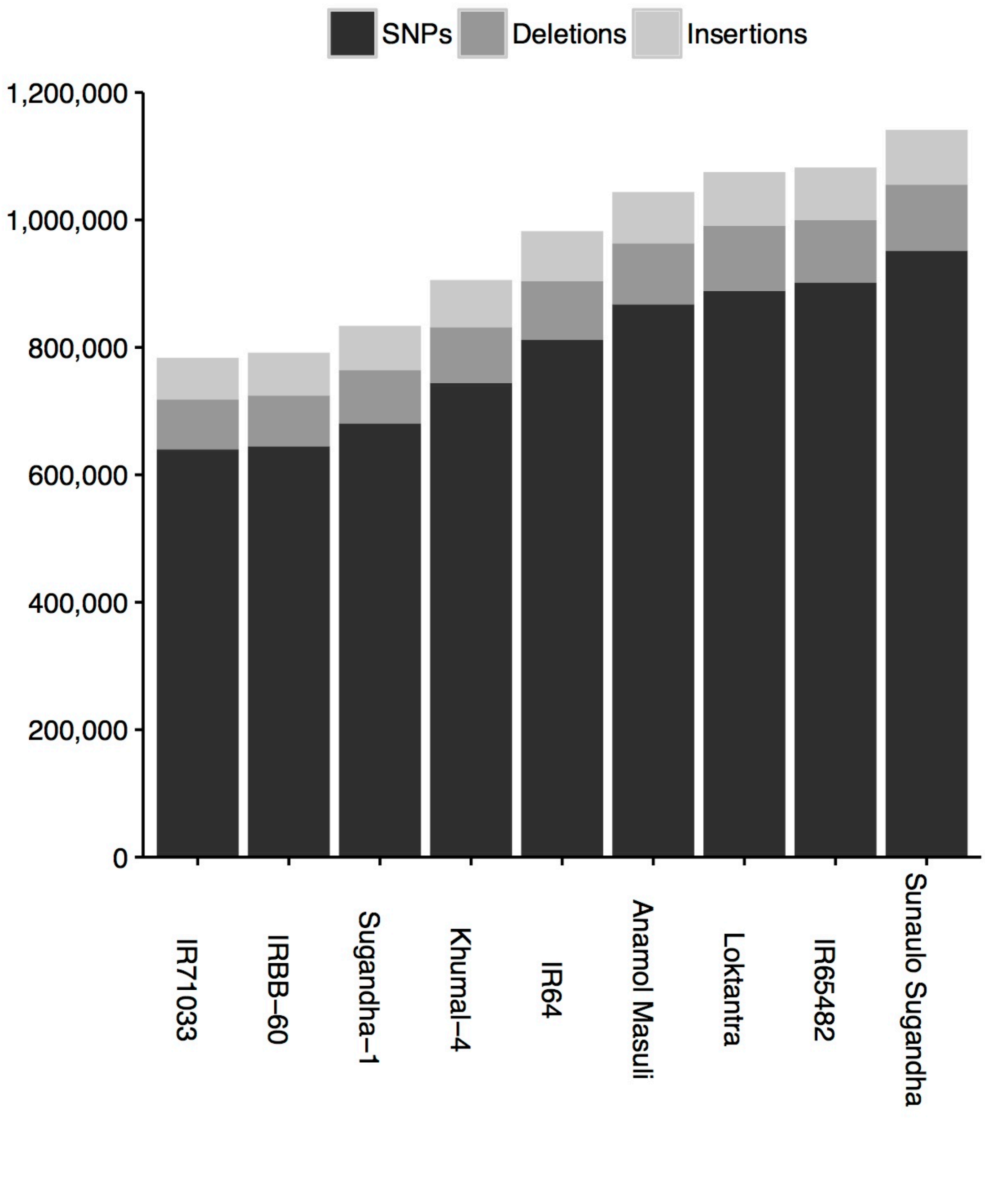

Fig. S4 Distribution of potential new rice KASP markers polymorphic between each rice line pair. Rows represent the chromosomes, subdivided into the different lines (ordered as indicated on chromosome 12), and columns the physical position. Subfigs show the distribution of markers informative for crosses against (a) IR64 (b) IR71033 (c) IR65482 (d) Sunaulo Sugandha (e) Anamol Masuli (f) Khumal-4 (g) IRBB-60 (h) Loktantra (i) Sugandha-1.

(a) IR64

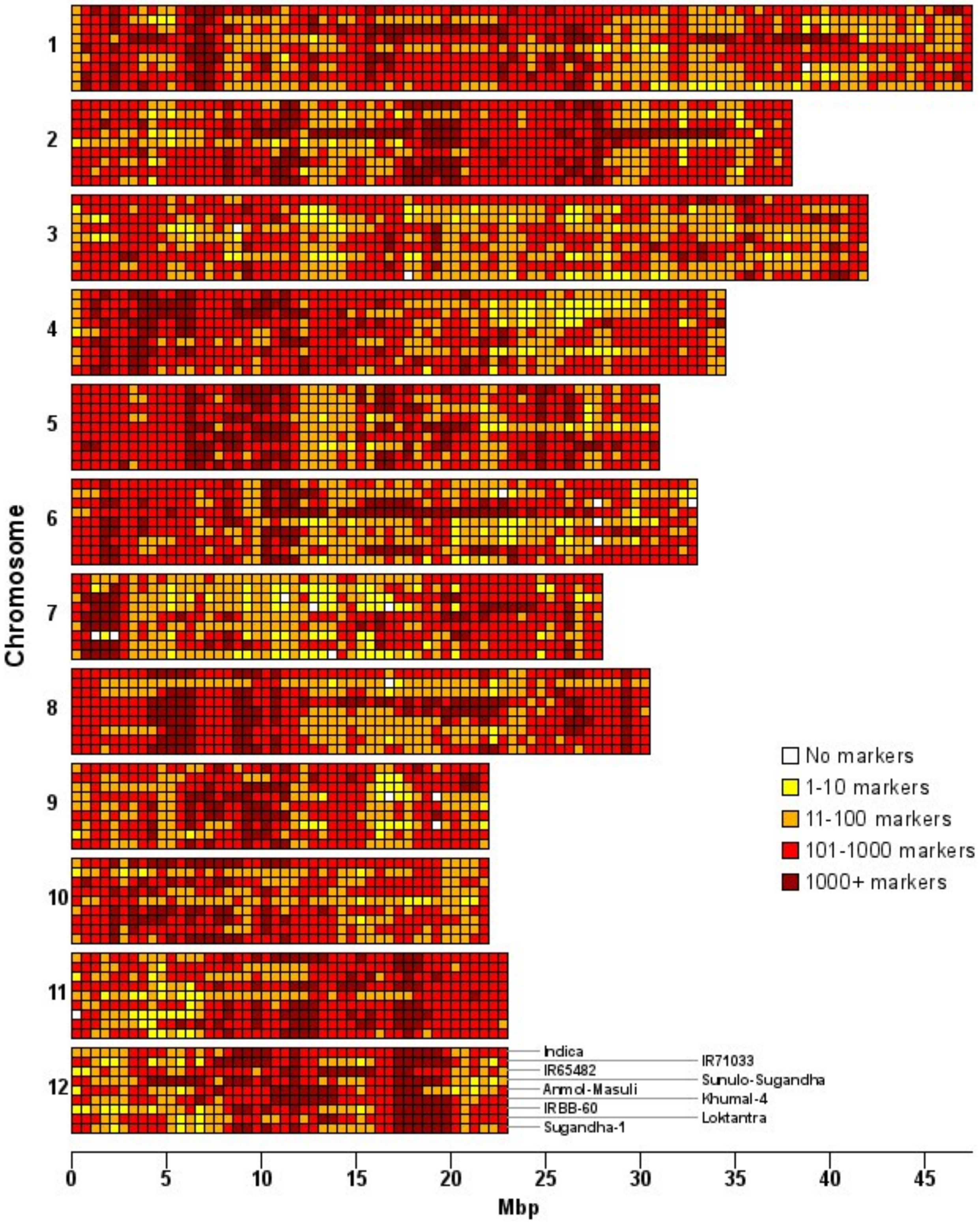

Chromosome
1
2
3
4
5
6
7
8
9
10
11
12
No markers
1-10 markers
11-100 markers
101-1000 markers
1000+ markers
Indica
IR65482
Anmol-Masuli
IRBB-60
Sugandha-1
IR71033
Sunulo-Sugandha
Khumal-4
Loktantra
0
5
10
15
20
25
30
35
40
45
Mbp

(b) IR71033

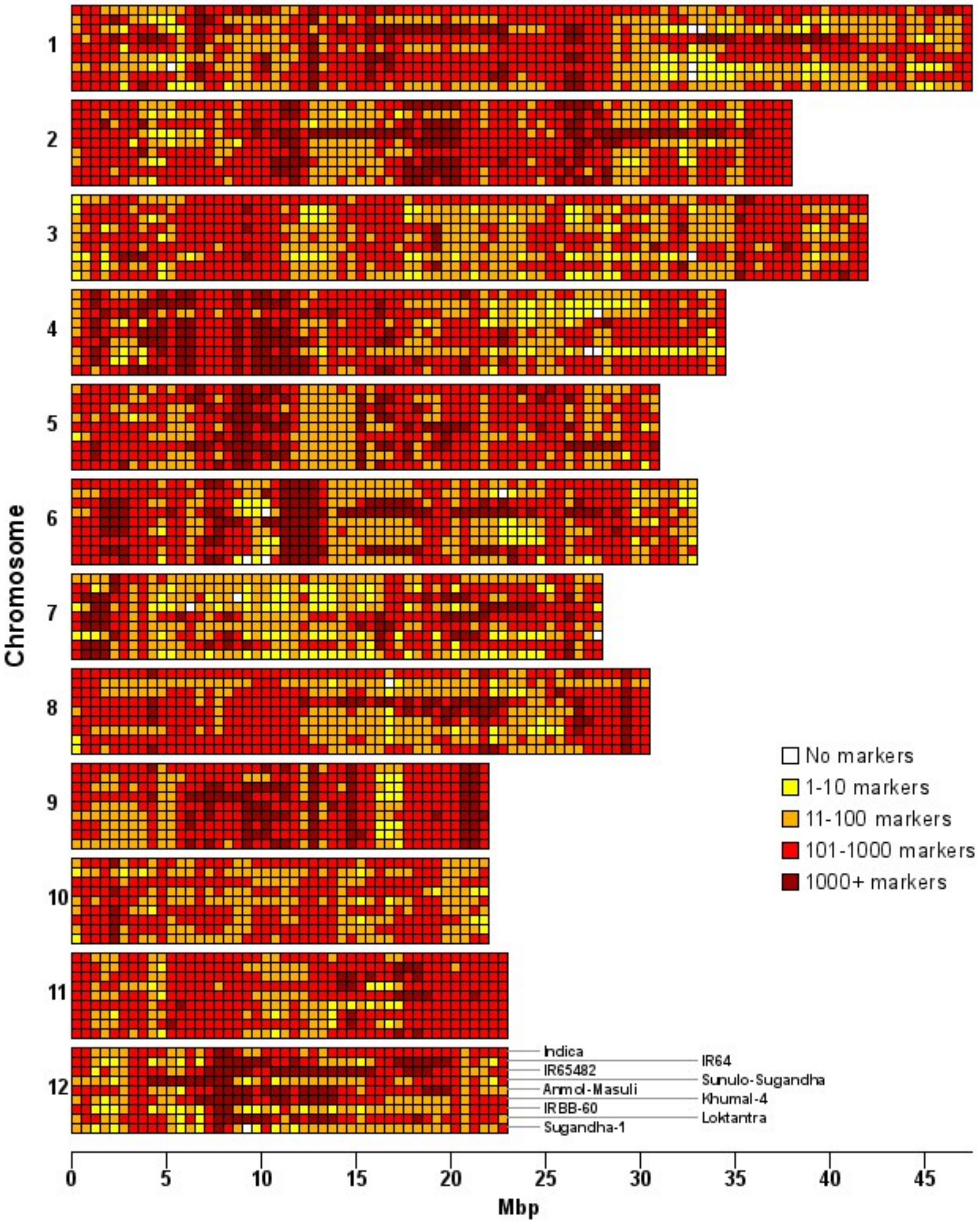
Chromosome
1
2
3
4
5
6
7
8
9
10
11
12
No markers
1-10 markers
11-100 markers
101-1000 markers
1000+ markers
Indica
IR65482
Anmol-Masuli
IRBB-60
Sugandha-1
IR64
Sunulo-Sugandha
Khumal-4
Loktantra
0
5
10
15
20
25
30
35
40
45
Mbp

(c) IR65482

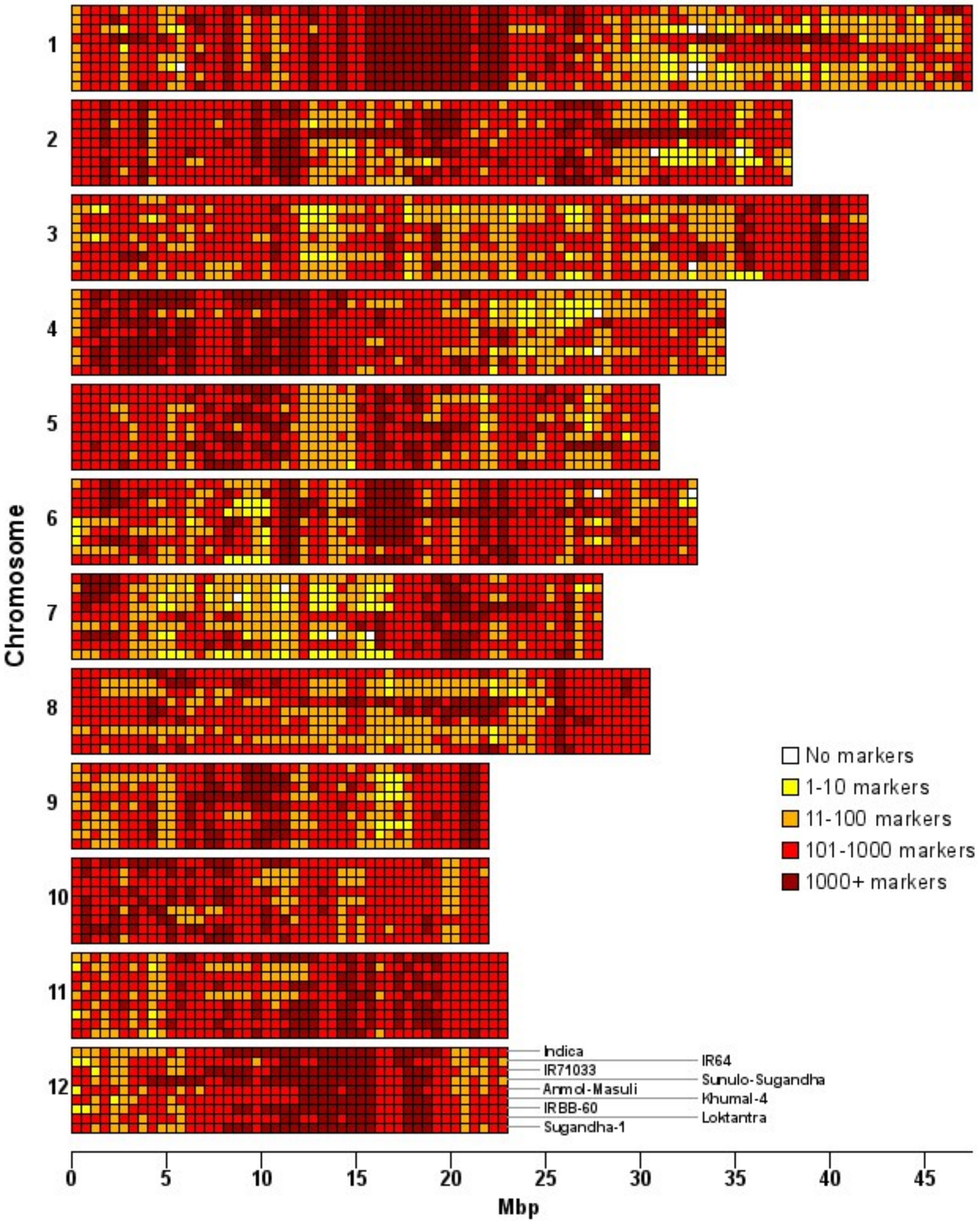

Chromosome
1
2
3
4
5
6
7
8
9
10
11
12
Indica
IR71033
Anmol-Masuli
IRBB-60
Sugandha-1
IR64
Sunulo-Sugandha
Khumal-4
Loktantra
No markers
1-10 markers
11-100 markers
101-1000 markers
1000+ markers
0
5
10
15
20
25
30
35
40
45
Mbp

(d) Sunaulo Sugandha

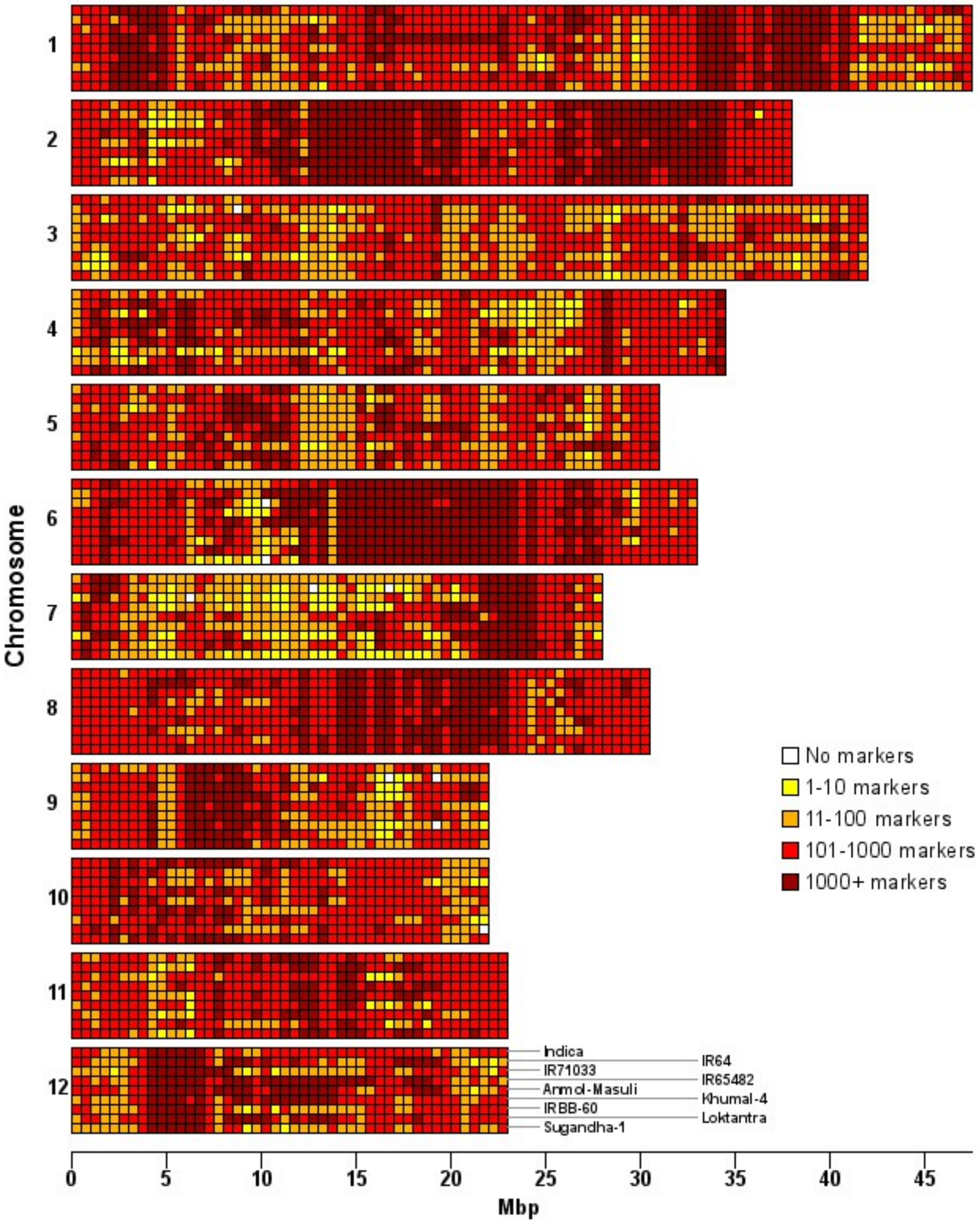

(e) Anamol Masuli

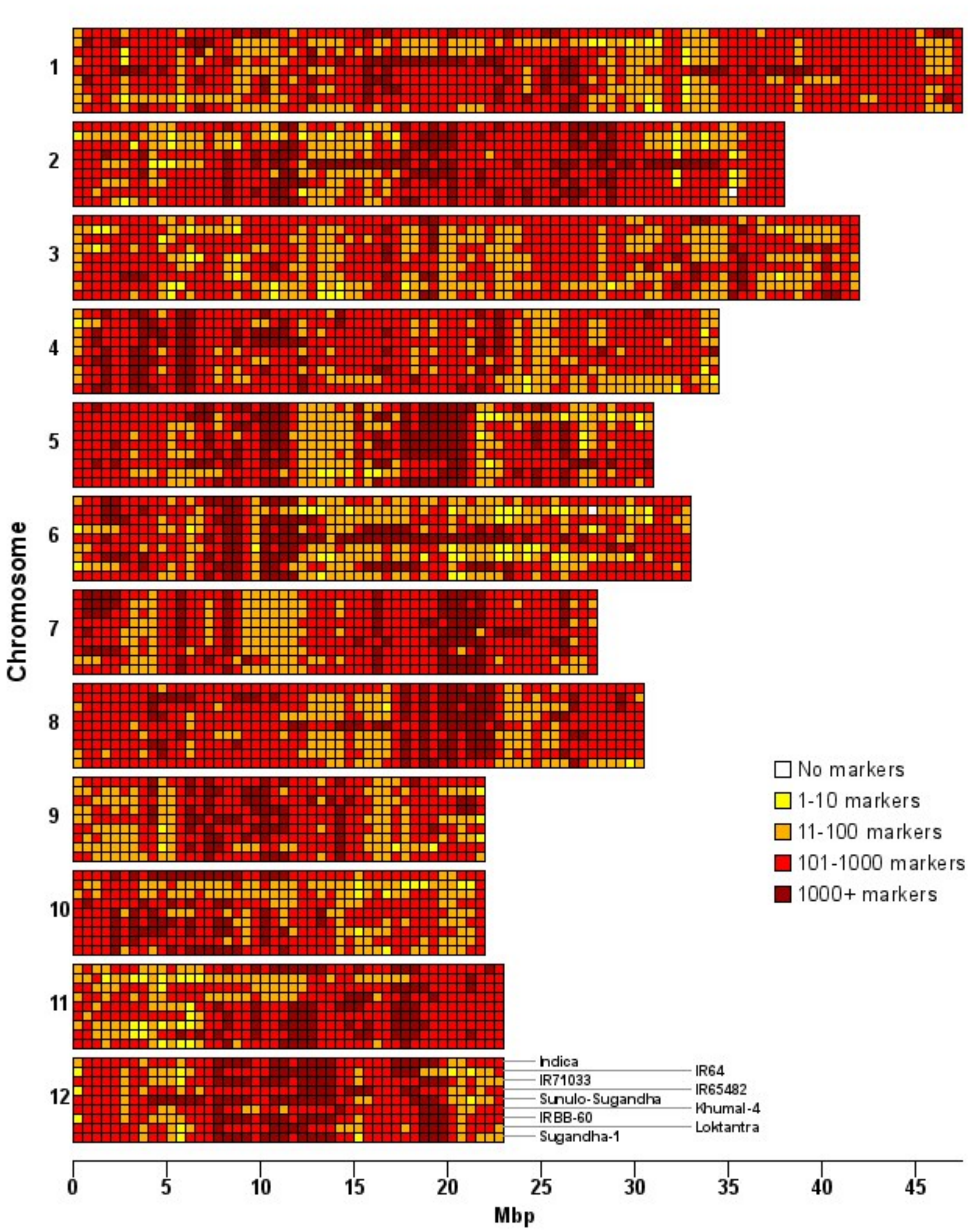

(f) Khumal-4

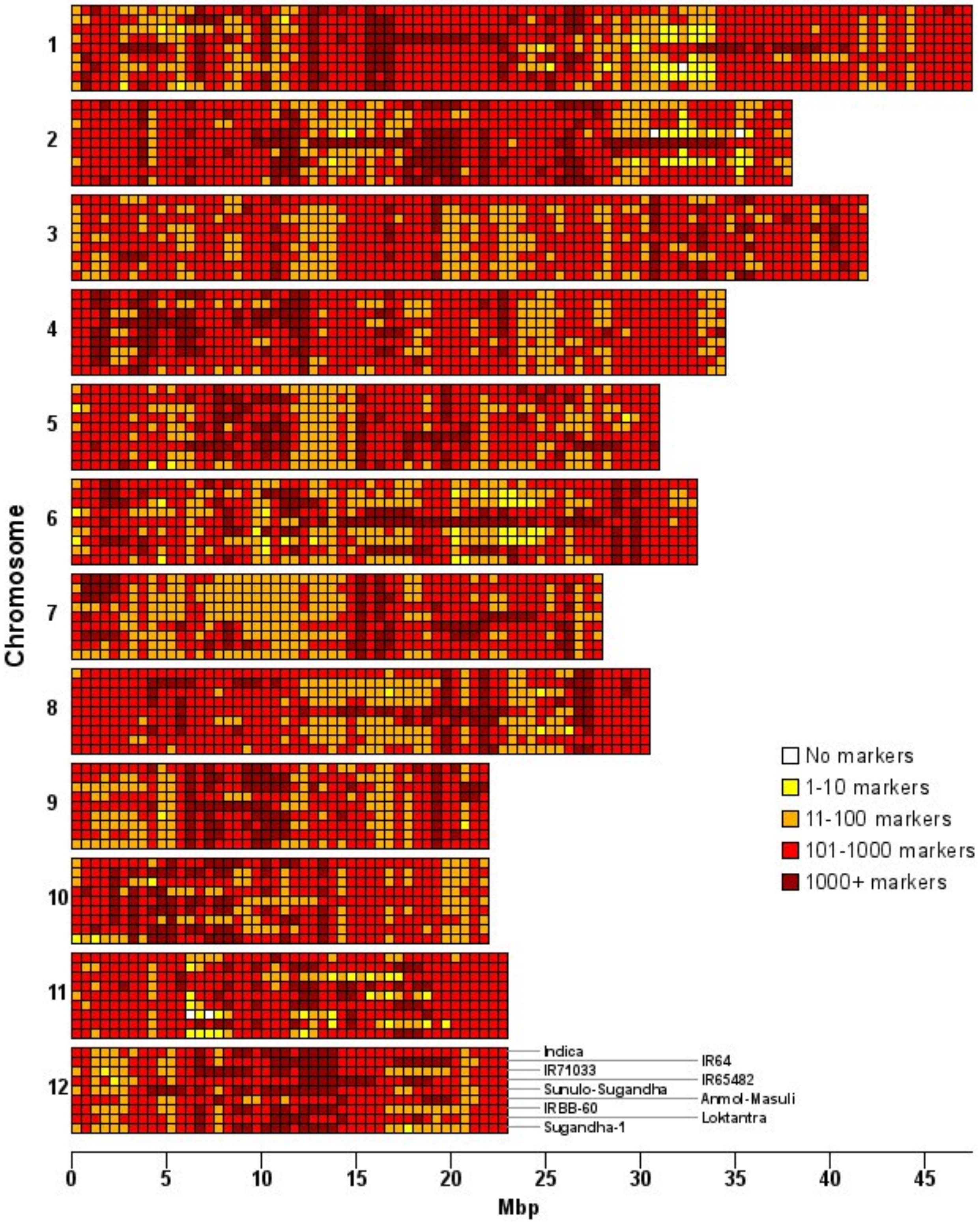

Chromosome
1
2
3
4
5
6
7
8
9
10
11
12
No markers
1-10 markers
11-100 markers
101-1000 markers
1000+ markers
Indica
IR71033
Sunulo-Sugandha
IRBB-60
Sugandha-1
IR64
IR65482
Anmol-Masuli
Loktantra
0
5
10
15
20
25
30
35
40
45
Mbp

(g) IRBB-60

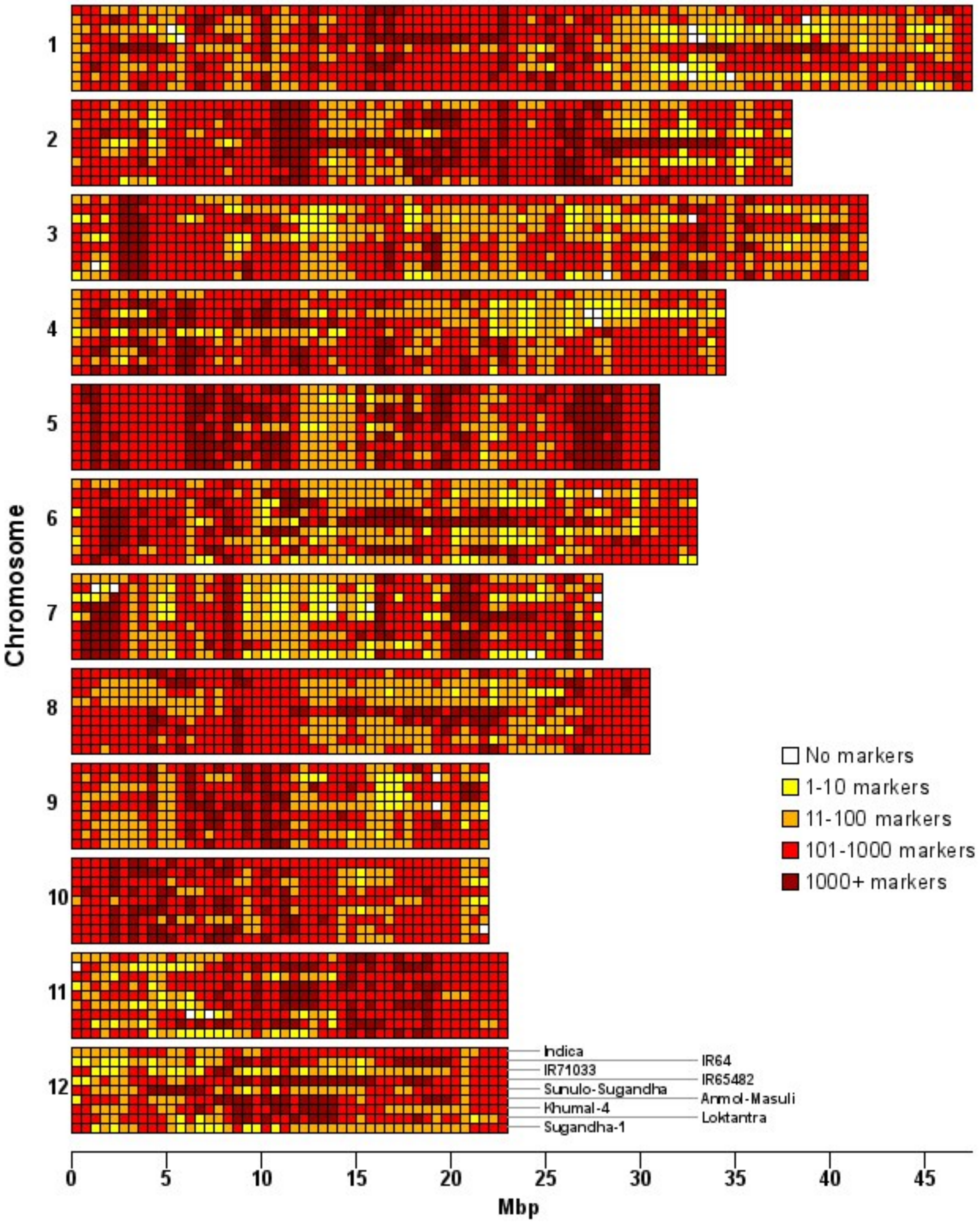
Chromosome
1
2
3
4
5
6
7
8
9
10
11
12
No markers
1-10 markers
11-100 markers
101-1000 markers
1000+ markers
Indica
IR71033
Sunulo-Sugandha
Khumal-4
Sugandha-1
IR64
IR65482
Anmol-Masuli
Loktantra
0
5
10
15
20
25
30
35
40
45
Mbp

(h) Loktantra

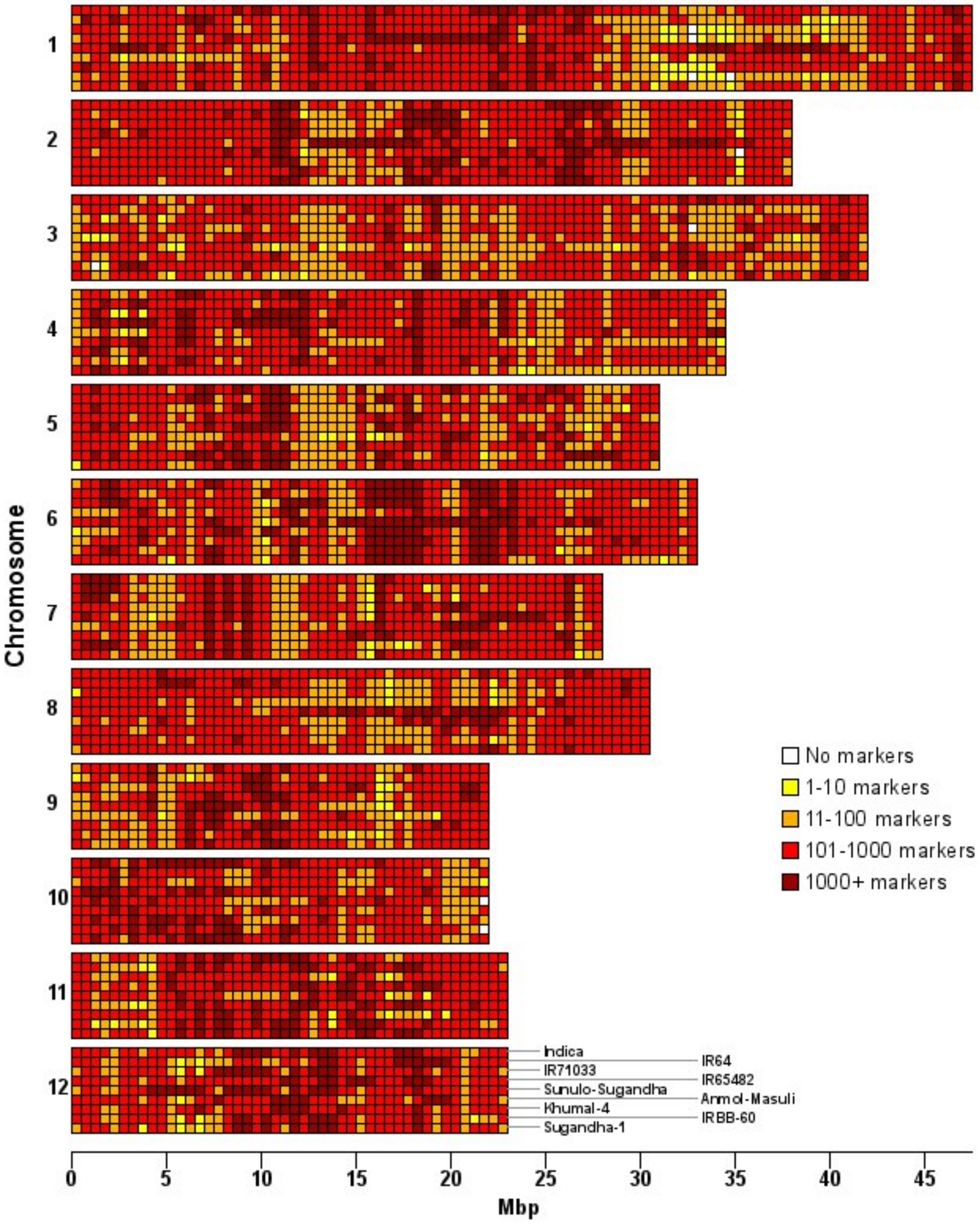

Chromosome
1
2
3
4
5
6
7
8
9
10
11
12
No markers
1-10 markers
11-100 markers
101-1000 markers
1000+ markers
Indica
IR71033
Sunulo-Sugandha
Khumal-4
Sugandha-1
IR64
IR65482
Anmol-Masuli
IRBB-60
0
5
10
15
20
25
30
35
40
45
Mbp

(i) Sugandha-1

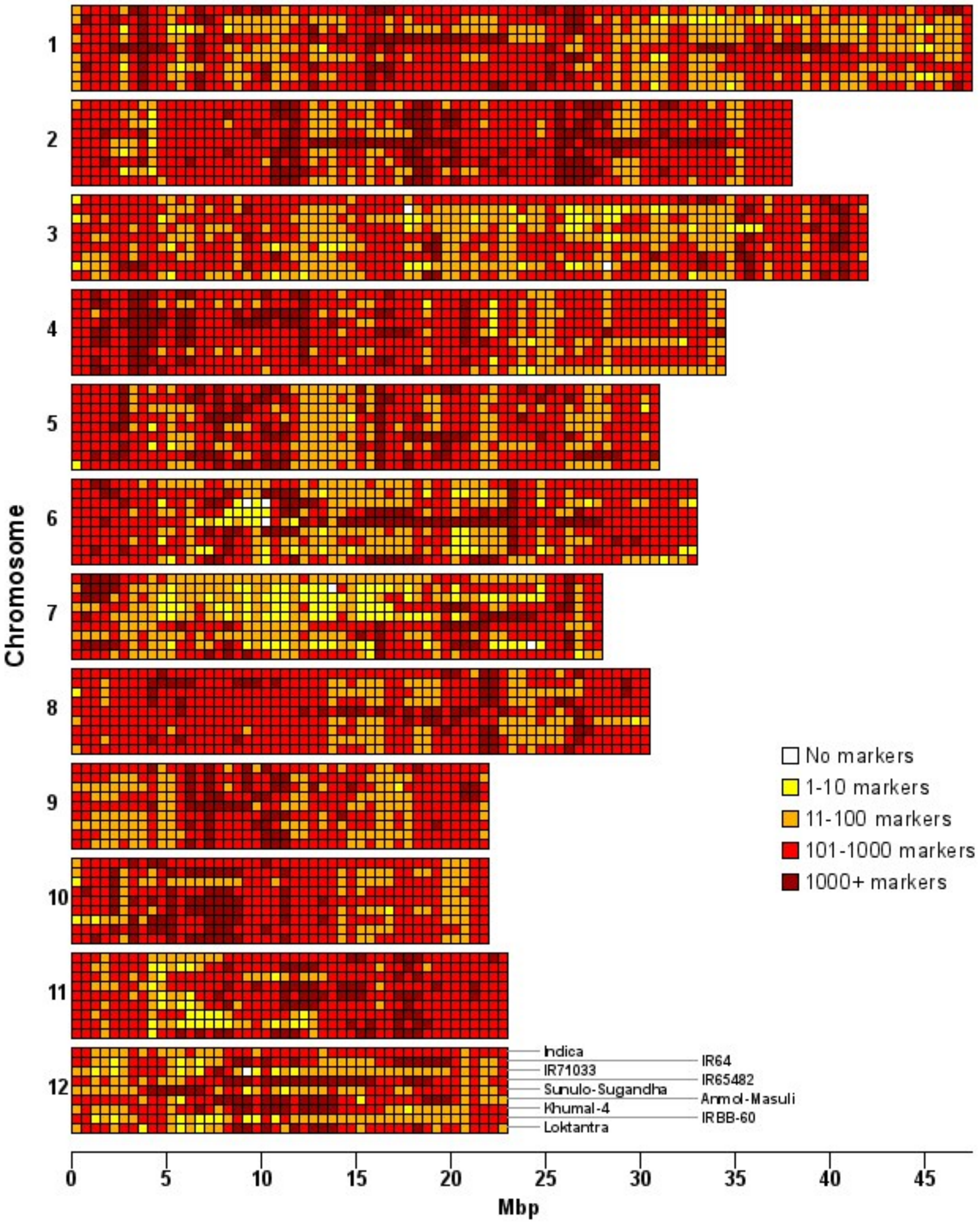
Chromosome
1
2
3
4
5
6
7
8
9
10
11
12
No markers
1-10 markers
11-100 markers
101-1000 markers
1000+ markers
Indica
IR71033
Sunulo-Sugandha
Khumal-4
Loktantra
IR64
IR65482
Anmol-Masuli
IRBB-60
0
5
10
15
20
25
30
35
40
45
Mbp

Fig. S5 Distribution of existing rice KASP markers polymorphic between each rice line pair. Rows represent the chromosomes, subdivided into the different lines (ordered as indicated on chromosome 12), and columns the physical position. Subfigs show the distribution of markers informative for crosses against (a) IR64 (b) IR71033 (c) IR65482 (d) Sunaulo Sugandha (e) Anamol Masuli (f) Khumal-4 (g) IRBB-60 (h) Loktantra (i) Sugandha-1.

(a) IR64

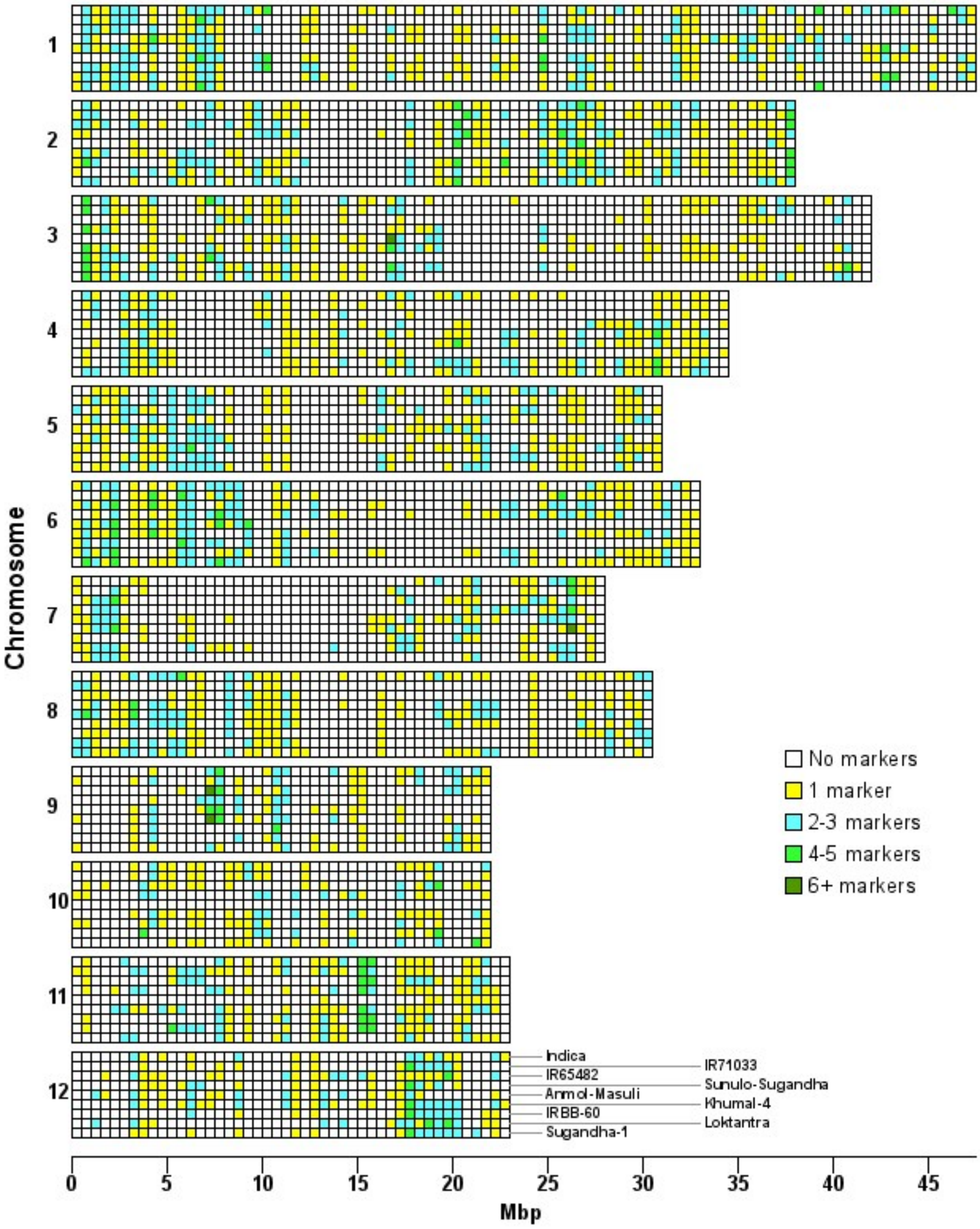
Chromosome
1
2
3
4
5
6
7
8
9
10
11
12
No markers
1 marker
2-3 markers
4-5 markers
6+ markers
Indica
IR65482
Anmol-Masuli
IRBB-60
Sugandha-1
IR71033
Sunulo-Sugandha
Khumal-4
Loktantra
0
5
10
15
20
25
30
35
40
45
Mbp

(b) IR71033

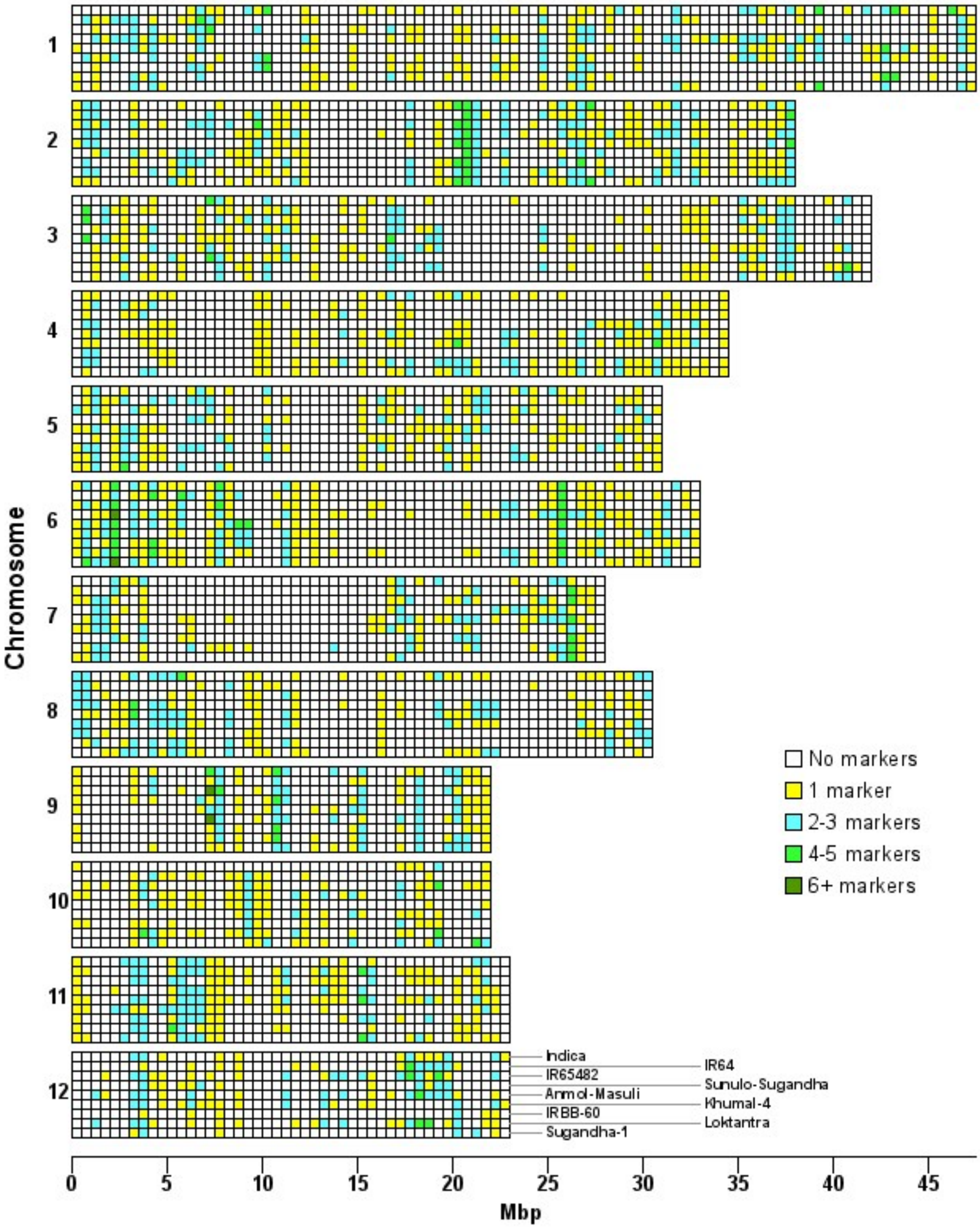
Chromosome
1
2
3
4
5
6
7
8
9
10
11
12
No markers
1 marker
2-3 markers
4-5 markers
6+ markers
Indica
IR65482
Anmol-Masuli
IRBB-60
Sugandha-1
IR64
Sunulo-Sugandha
Khumal-4
Loktantra
0
5
10
15
20
25
30
35
40
45
Mbp

(c) IR65482

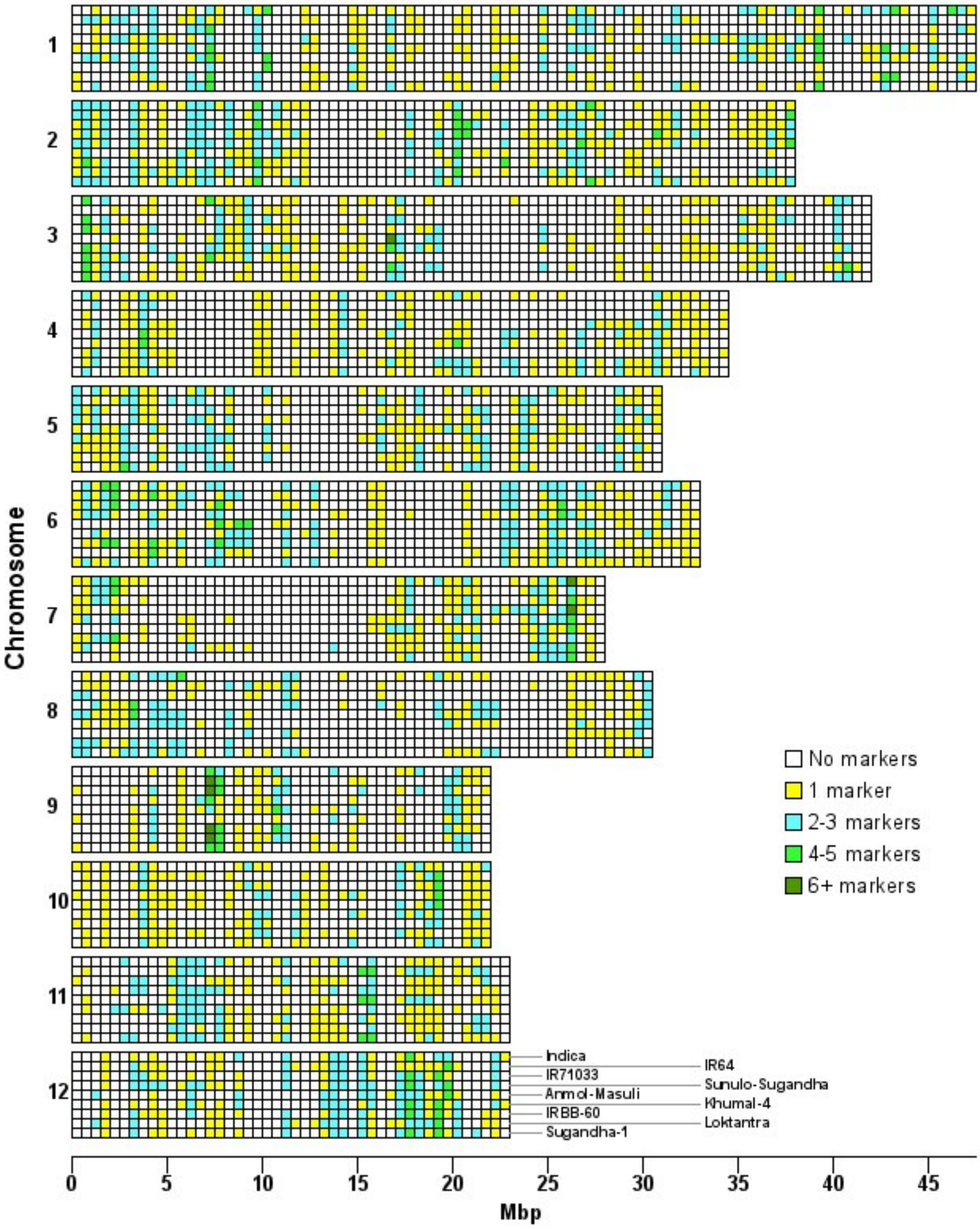

Chromosome
1
2
3
4
5
6
7
8
9
10
11
12
No markers
1 marker
2-3 markers
4-5 markers
6+ markers
Indica
IR71033
Anmol-Masuli
IRBB-60
Sugandha-1
IR64
Sunulo-Sugandha
Khumal-4
Loktantra
0
5
10
15
20
25
30
35
40
45
Mbp

(d) Sunaulo Sugandha

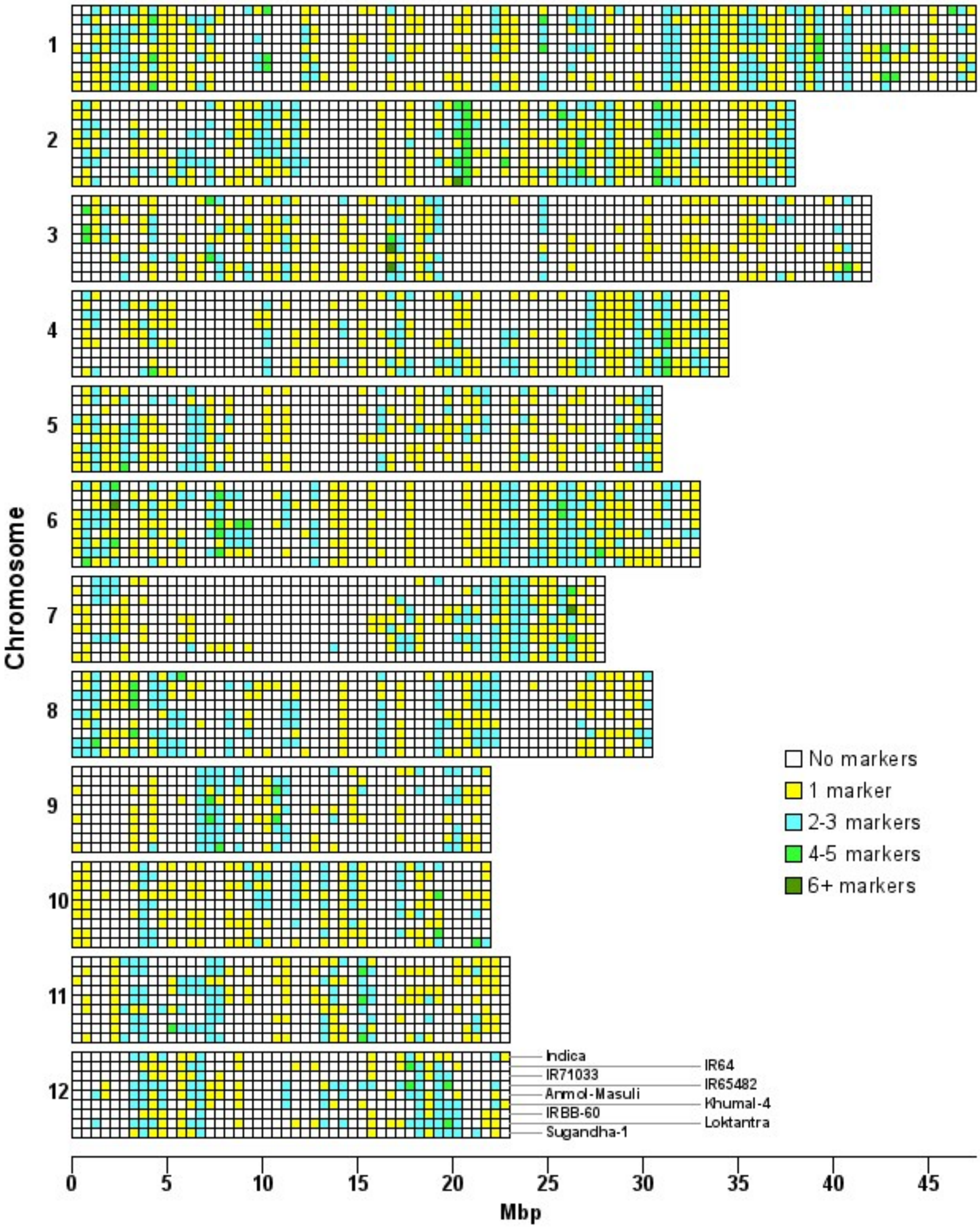

(e) Anamol Masuli

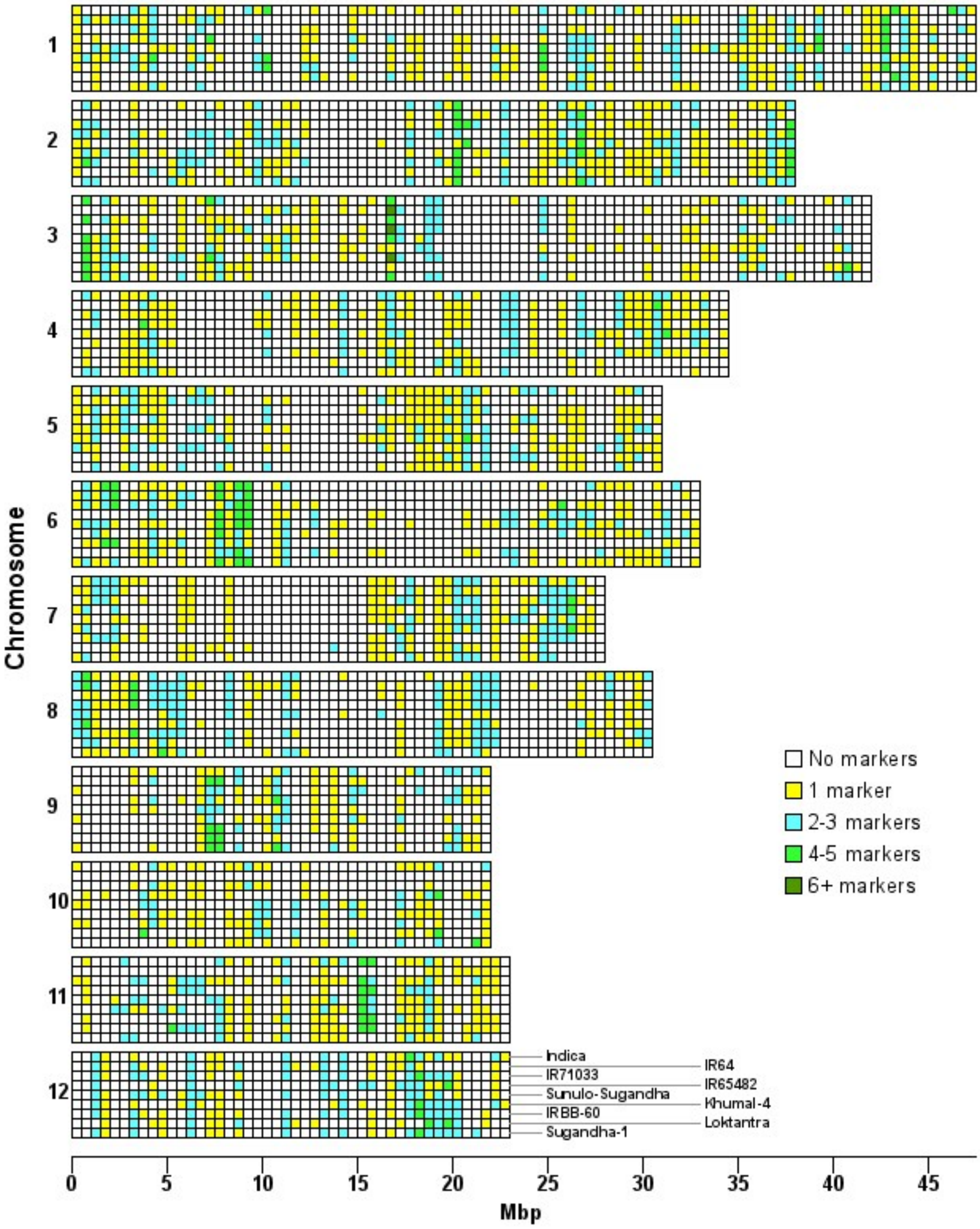

(f) Khumal-4

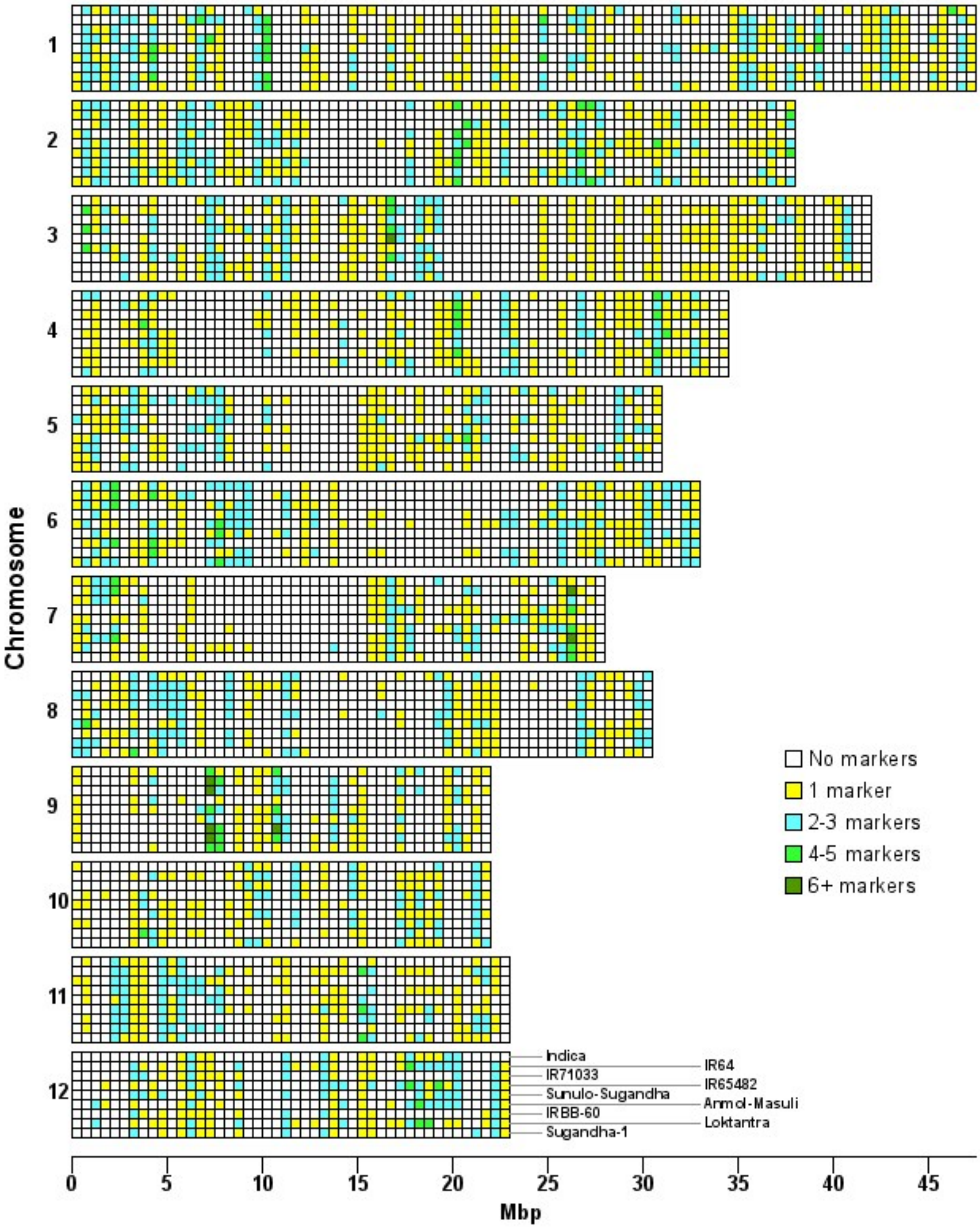
Chromosome
1
2
3
4
5
6
7
8
9
10
11
12
No markers
1 marker
2-3 markers
4-5 markers
6+ markers
Indica
IR71033
Sunulo-Sugandha
IRBB-60
Sugandha-1
IR64
IR65482
Anmol-Masuli
Loktantra
0
5
10
15
20
25
30
35
40
45
Mbp

(g) IRBB-60

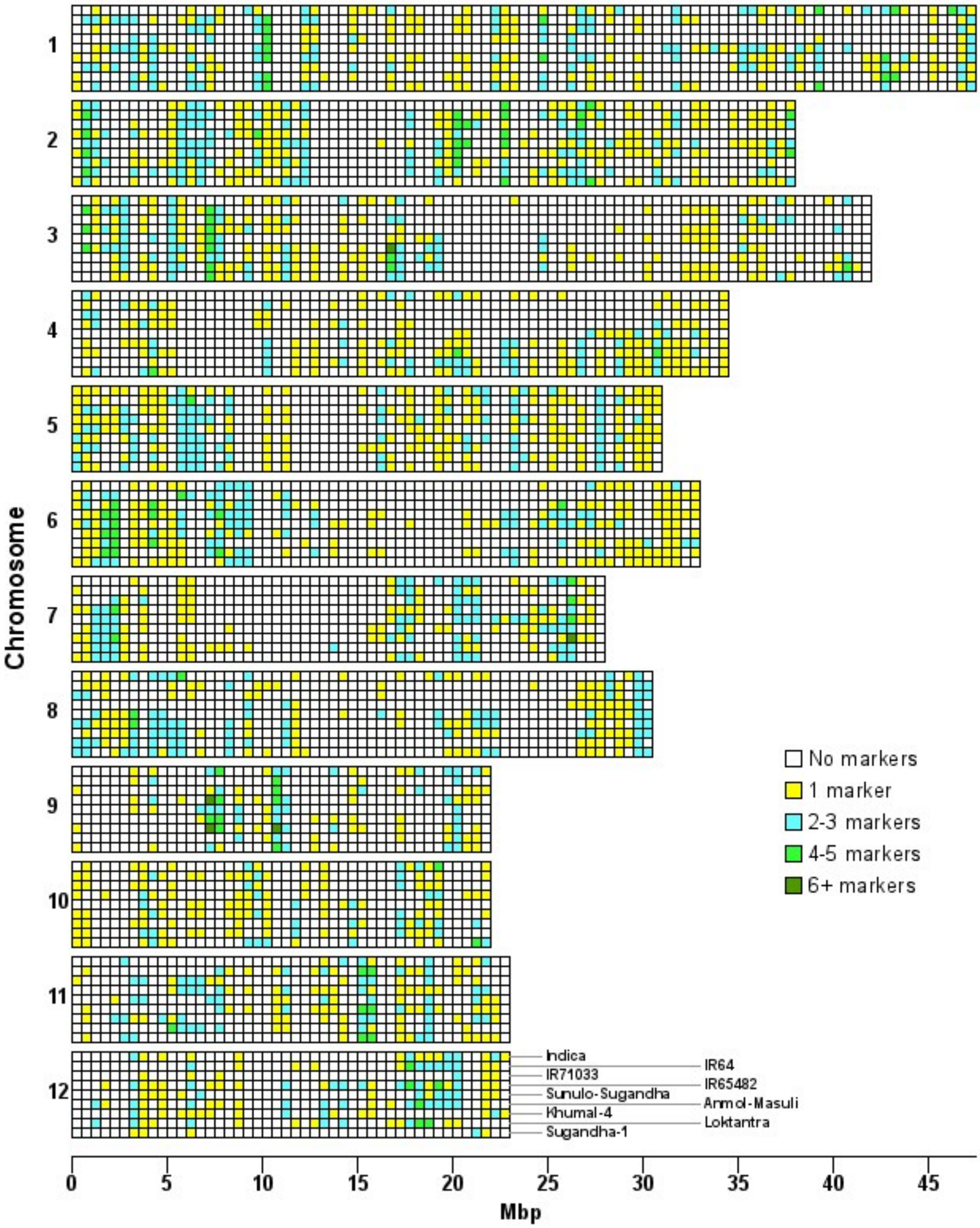

Chromosome
1
2
3
4
5
6
7
8
9
10
11
12
No markers
1 marker
2-3 markers
4-5 markers
6+ markers
Indica
IR71033
Sunulo-Sugandha
Khumal-4
Sugandha-1
IR64
IR65482
Anmol-Masuli
Loktantra
0
5
10
15
20
25
30
35
40
45
Mbp

(h) Loktantra

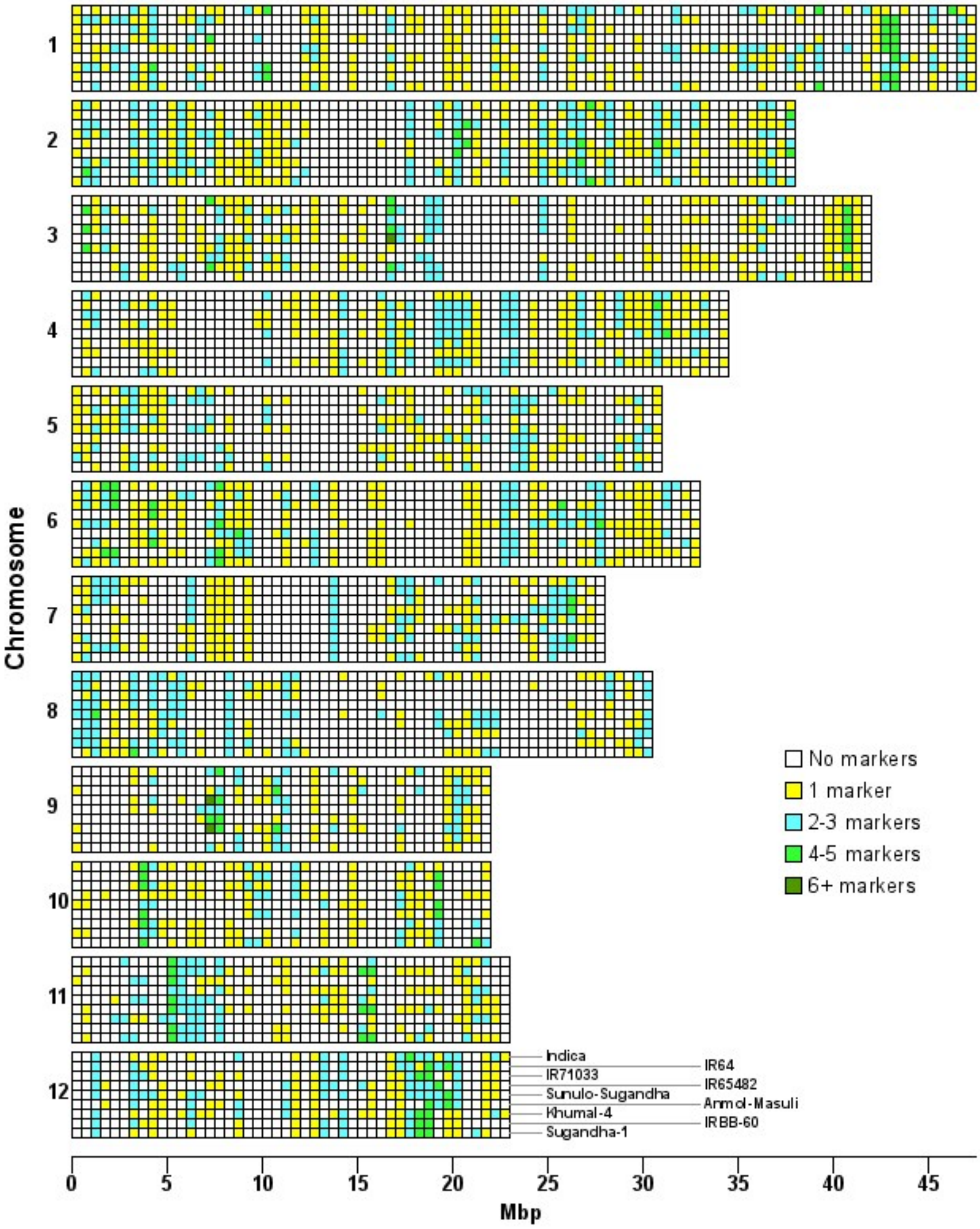
Chromosome
1
2
3
4
5
6
7
8
9
10
11
12
Mbp
0
5
10
15
20
25
30
35
40
45
No markers
1 marker
2-3 markers
4-5 markers
6+ markers
Indica
IR71033
Sunulo-Sugandha
Khumal-4
Sugandha-1
IR64
IR65482
Anmol-Masuli
IRBB-60

(i) Sugandha-1

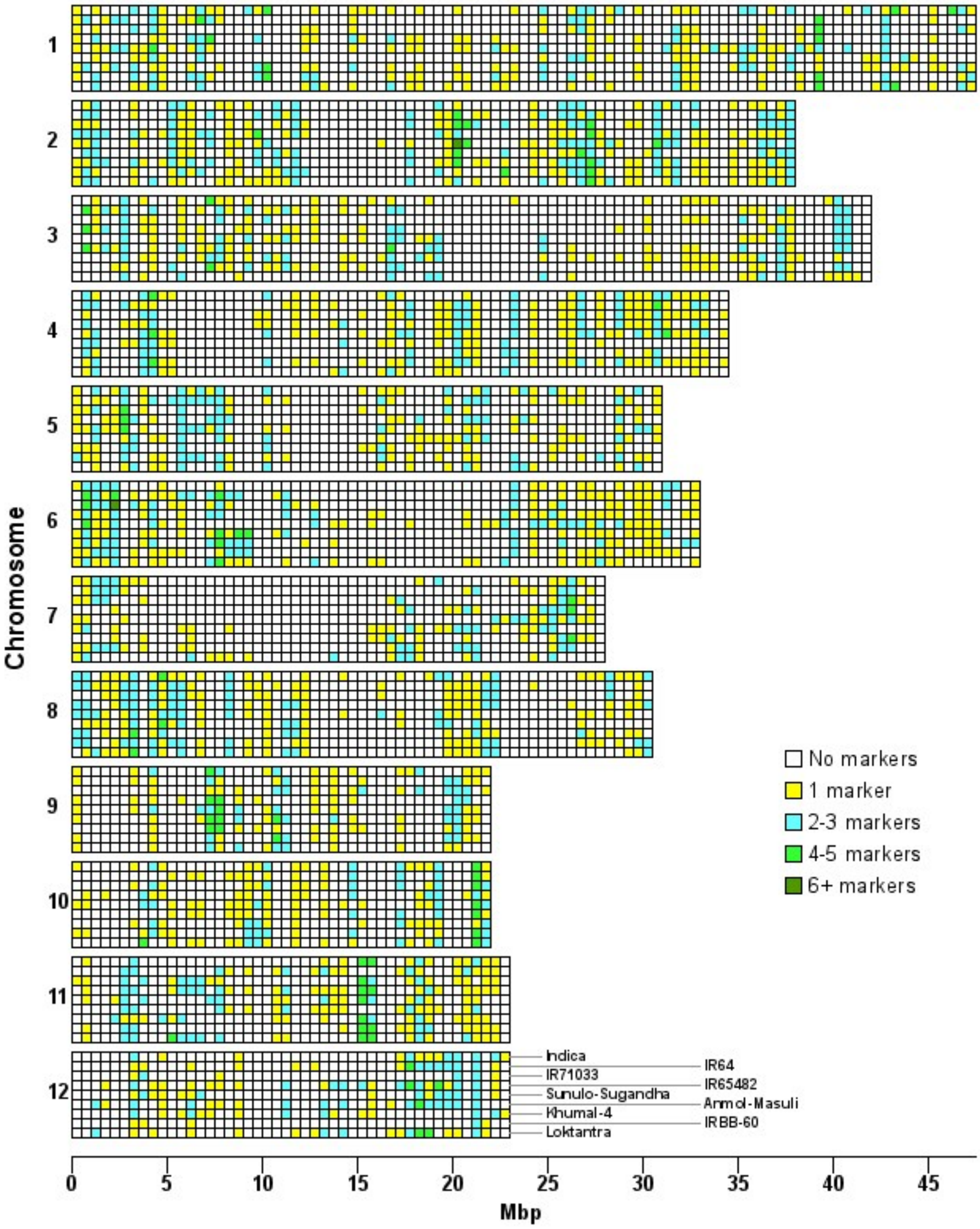
Chromosome
1
2
3
4
5
6
7
8
9
10
11
12
No markers
1 marker
2-3 markers
4-5 markers
6+ markers
Indica
IR64
IR71033
IR65482
Sunulo-Sugandha
Anmol-Masuli
Khumal-4
IRBB-60
Loktantra
0
5
10
15
20
25
30
35
40
45
Mbp

Fig. S6 Distribution of distances between consecutive informative KASP markers, for new and existing markers, for all combined pairwise combinations of rice lines used in this study. Vertical bars represent the medians with boxes extending from the 25th to 75th percentiles. Whiskers extend from the 5th to 95th percentiles, dots represent the minimum and maximum distances.

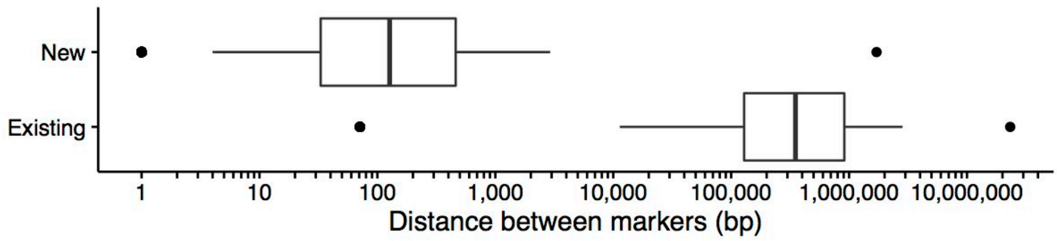

Supplement: Supplementary file 2 — Figure. S1 Overview of the criteria used for identification of potential KASP markers from variations identified using SAMtools. Figure. S2 Number of variations identified at the same positions relative to the indica reference in all sequenced rice lines (maximum nine). Figure. S3 Number of variations (homozygous SNPs, insertions and deletions) identified between each of the nine sequenced rice lines and the indica reference genome. Figure. S4 Distribution of potential new rice KASP markers polymorphic between each rice line pair. Rows represent the chromosomes, subdivided into the different lines (ordered as indicated on chromosome 12), and columns the physical position. SubFigures show the distribution of markers informative for crosses against (a) IR64 (b) IR71033 (c) IR65482 (d) Sunaulo Sugandha (e) Anamol Masuli (f) Khumal-4 (g) IRBB-60 (h) Loktantra (i) Sugandha-1. Figure. S5 Distribution of existing rice KASP markers polymorphic between each rice line pair. Rows represent the chromosomes, subdivided into the different lines (ordered as indicated on chromosome 12), and columns the physical position. SubFigures show the distribution of markers informative for crosses against (a) IR64 (b) IR71033 (c) IR65482 (d) Sunaulo Sugandha (e) Anamol Masuli (f) Khumal-4 (g) IRBB-60 (h) Loktantra (i) Sugandha-1. Figure. S6 Distribution of distances between consecutive informative KASP markers, for new and existing markers, for all combined pairwise combinations of rice lines used in this study. Vertical bars represent the medians with boxes extending from the 25th to 75th percentiles. Whiskers extend from the 5th to 95th percentiles, dots represent the minimum and maximum distances. (PDF 4943 kb) [file 11032_2018_777_MOESM2_ESM.pdf]
