# Supplementary material for: Accelerating public sector rice breeding with high-density KASP markers derived from whole genome sequencing of indica rice
Source: Mol Breed. 2018 Mar 7;38(4):38. doi: 10.1007/s11032-018-0777-2 (PMC5842261; doi:10.1007/s11032-018-0777-2)
Supplement: Supplementary file 3 — Table S1 Details of nine indica rice cultivars and breeding lines used for whole genome NGS. Table S2 Mapping rates, genome coverage and read depth of the nine sequenced rice lines, for all mapped reads and for uniquely-mapped reads only. Only those reads with both pairs aligning in the expected orientation were included. Table S3 Distribution of distances (bp) between consecutive informative existing KASP markers (previously developed using chip-based technology) for all rice line pairings. Table S4 Distribution of distances (bp) between consecutive informative potential new KASP markers (developed here using NGS data) for all rice line pairings. Table S5 Summary of KASP assay validation results. Counts are given of the number of unique marker-cross combinations that have been validated (or otherwise), split according to whether or not the markers met our bioinformatics filtering criteria, and between new and existing markers. Table S6 Details of marker-cross combinations tested for validation of 46 new and 75 existing KASP assays (N.B. some failed assays may be due to lack of polymorphism.) (PDF 443 kb) [file 11032_2018_777_MOESM3_ESM.pdf]

Supplementary Tables

Accelerating public sector rice breeding with high-density KASP markers derived from whole genome sequencing of *indica* rice

Katherine A. Steele · Mark J. Quinton-Tulloch · Resham B. Amgai · Rajeev Dhakal · Shambhu P. Khatiwada · Darshna Vyas · Martin Heine · J. R. Witcombe

K.A.Steele (corresponding author: email k.a.steele@bangor.ac.uk, Tel 00 44 1248 388655)
M.J. Quinton-Tulloch · J.R. Witcombe
School of the Environment, Natural Resources and Geography, SENRGY, Bangor University, Bangor, Gwynedd, LL57 2UW, UK

R.B. Amgai · S.P. Khatiwada
Nepal Agricultural Research Council, Biotechnology Division, PO Box No. 1135 Kathmandu, Nepal

R. Dhakal
Anamolbiu Private Ltd., P.O. Box 28, Jagritichok, Bharatpur-11, Chitwan, Nepal. Current address: LI-BIRD, Head Office: PO Box 324, Gairapatan, Pokhara, Kaski, Nepal

D. Vyas
LGC Genomics, Units 1 & 2, Trident Industrial Estate, Pindar Road, Hoddesdon, Herts, EN11 0WZ, UK

M. Heine
LGC Genomics LGC Genomics TGS Haus 8, Ostendstr. 25, 12459 Berlin, Germany; ***Current address****: NuGEN Technologies Inc.201 Industrial Road, Suite 310 San Carlos, CA 94070, USA*

Table S1 Details of nine *indica* rice cultivars and breeding lines used for whole genome NGS.

Line/variety name (short name)	Release status /description	Pedigree information
IR64	Released in many countries in Asia from 1985. Disease resistance donor.	Pedigree includes 18 landraces or wild rice species*
IR65482-4-136-2-2 (IR65482)	Disease resistance donor	IR31917-45-3-2-2/O. Australiensis
IR71033-4-1-127B (IR71033)	Disease resistance donor	IR31917-45-3-2/O. Minuta//IR31917-45-3-2
IRBB-60	Disease resistance donor	IR 24*5/IR 1545-339**
NR1487-2-1-2-2-1-1 (Loktantra)	Released for Nepal Terai in 2006. Disease resistance donor.	Mahsuri/IR4557-6-3-2
Anamol Masuli	Proposed for release in Nepal Terai	Masuli//Kalinga III/IR64
Khumal-4	Released for Nepal mid hills in 1987	IR28/Pokhreli Masino
Sugandha-1	Not released but adapted to Nepal Terai	From outcrossed Pusa Basmati-1 population.
Sunaulo Sugandha	Released for Nepal mid hills in 2007	From outcrossed Pusa Basmati-1 population.

*Khush GS (2005) IR varieties and their impact. Int. Rice Res. Inst. (p139)

** Huang N, Angeles ER, Domingo J, Magpantay G, Singh S, Zhang G, Kumaravadivel N, Bennett J, Khush GS (1997) Pyramiding of bacterial blight resistance genes in rice: Marker-assisted selection using RFLP and PCR. Theor. Appl. Genet. 95: 313–320.

Table S2 Mapping rates, genome coverage and read depth of the nine sequenced rice lines, for all mapped reads and for uniquely-mapped reads only. Only those reads with both pairs aligning in the expected orientation were included.

Genotype	Mapping rate (%)	Genome coverage (%)	Gene region coverage (%)	Depth	Gene region depth
IR64	92.17	89.16	94.23	61.04	62.31
IR71033	91.89	89.63	94.42	55.21	55.22
IR65482	90.82	88.93	93.97	54.44	54.76
Sunaulo Sugandha	91.34	89.15	94.22	56.76	58.32
Anamol Masuli	90.94	88.94	94.06	45.98	47.92
Khumal-4	92.6	89.2	94.22	55.2	55.63
IRBB-60	92.82	89.37	94.4	60.4	58.68
Loktantra	93.8	88.68	93.87	81.33	82.78
Sugandha-1	92.44	89.33	94.33	57.2	57.44

Table S3 Distribution of distances (bp) between consecutive informative existing KASP markers (previously developed using chip-based technology) for all rice line pairings.

Variety pair	Minimum	Maximum	Mean	Standard deviation	Median	Percentiles			
						5th	25th	75th	95th
IR64 / Indica	71	14377260	763484	1276446	354565	16291	135887	879400	2487683
IR64/IR71033	92	13940287	1003025	1947129	359116	14691	147115	1020040	3491611
IR64 / IR65482	162	14396946	878054	1549677	375908	16998	130451	979761	3375072
IR64 / Sunulo-Sugandha	71	18496087	759273	1415779	336563	17738	138504	837449	2774326
IR64 / Anmol-Masuli	162	23236882	809779	1854474	343097	4106	138836	846463	2427149
IR64 / Khumal-4	71	11682731	710592	1124595	348605	7850	140686	866083	2331663
IR64 / IRBB-60	71	22647712	945650	2115950	353663	17779	121015	892008	3459913
IR64 / Loktantra	71	10036748	750240	1209863	316955	9368	118867	799295	3240577
IR64 / Sugandha-1	71	17887867	832211	1749765	338791	17738	109361	846463	2932075
IR71033 / Indica	71	16864278	959493	1753385	368925	6797	127509	1061819	3433720
IR71033 / IR65482	92	12955480	898190	1600624	367336	3666	146951	1002187	3465116
IR71033 / Sunulo-Sugandha	71	12955480	800160	1271502	351185	19083	152021	869615	3199279
IR71033 / Anmol-Masuli	77	12341964	776455	1319080	352429	3666	109422	903305	2756393
IR71033 / Khumal-4	71	11276409	814688	1259075	370819	6867	128859	977794	2787502
IR71033 / IRBB-60	71	16771212	994786	2016261	354014	14691	107579	988468	3656490
IR71033 / Loktantra	71	14189717	807867	1356259	354014	7600	106358	952952	2876165
IR71033 / Sugandha-1	71	17693643	944703	1862453	335758	9368	106358	977794	3946741
IR65482 / Indica	177	13736002	747700	1158598	359674	14351	140512	884055	2998216
IR65482 / Sunulo-Sugandha	177	14396946	723507	1166356	334180	11221	135969	758652	2679446
IR65482 / Anmol-Masuli	77	7985632	744759	1068816	339713	10764	113231	927743	2622788
IR65482 / Khumal-4	177	10519024	777463	1161750	367336	11388	146503	982488	2701926
IR65482 / IRBB-60	162	14782009	811459	1588303	359909	8228	127508	827601	2971275
IR65482 / Loktantra	145	17913072	819921	1390627	372992	9368	130832	985360	2870291
IR65482 / Sugandha-1	162	15119635	792977	1375822	339094	9368	118269	953720	2821558
Sunulo-Sugandha / Indica	222	14377260	743936	1350692	348497	16133	147790	760158	2726792
Sunulo-Sugandha / Anmol-Masuli	71	7579336	695032	974935	344004	4800	127508	911373	2496863
Sunulo-Sugandha / Khumal-4	222	9240170	730168	1019329	330923	12650	140512	920909	2687274
Sunulo-Sugandha / IRBB-60	222	11532675	757686	1431872	314248	19083	121410	675426	3189446
Sunulo-Sugandha / Loktantra	145	10036748	688475	1072210	326411	10202	114044	733766	2696332
Sunulo-Sugandha / Sugandha-1	222	19147424	748958	1388430	348497	15186	128501	804158	2451338
Anmol-Masuli / Indica	71	13119692	749654	1194224	357772	10619	128859	846463	2725817
Anmol-Masuli / Khumal-4	71	11682731	773294	1291760	349835	14225	146503	945052	2356103
Anmol-Masuli / IRBB-60	71	19930483	715769	1327094	343097	9368	127509	852486	2306264
Anmol-Masuli / Loktantra	71	16822221	893630	1688951	367166	4056	122188	922816	3283897
Anmol-Masuli / Sugandha-1	71	18016506	840991	1522915	361037	4106	107138	1048136	2773161
Khumal-4 / Indica	325	10665551	841786	1314161	380402	19502	143290	982488	3133063
Khumal-4 / IRBB-60	162	11682731	788695	1272427	360635	12167	109361	912015	2814465
Khumal-4 / Loktantra	145	10519024	765646	1171133	353529	7600	123989	952758	2593244
Khumal-4 / Sugandha-1	162	13794913	843176	1355651	367166	14150	157389	1070570	2787502
IRBB-60 / Indica	162	15342412	928943	1779281	360635	12011	122981	993796	3283897
IRBB-60 / Loktantra	145	15375983	779185	1317081	352429	15636	128047	1014448	2758484
IRBB-60 / Sugandha-1	370	15304922	840925	1632458	338097	16291	123989	879400	2829404
Loktantra / Indica	145	7001248	738250	992005	363647	11388	148710	910171	2705200
Loktantra / Sugandha-1	145	10281290	903159	1412004	364550	11388	120917	1088474	3375072
Sugandha-1 / Indica	162	15523689	881525	1556095	404000	14351	135400	1017952	3148836

Table S4 Distribution of distances (bp) between consecutive informative potential new KASP markers (developed here using NGS data) for all rice line pairings.

Variety pair	Minimum	Maximum	Mean	Standard deviation	Median	Percentiles			
						5th	25th	75th	95th
IR64 / Indica	1	196295	1011.3	4119.4	137	4	35	505	3888
IR64 / IR71033	1	946417	1594.4	11028.6	130	4	33	485	3884
IR64 / IR65482	1	702167	1099.9	7906.7	121	4	32	442	2692
IR64 / Sunaulo Sugandha	1	971533	875.4	7414.3	115	4	31	410	2114
IR64 / Anamol Masuli	1	648660	1368.9	9382.9	129	4	32	477	3372
IR64 / IRBB-60	1	897618	1192.3	9636.2	127	4	33	448	2549
IR64 / Khumal-4	1	529682	1038.2	5566.3	131	4	33	474	2917
IR64 / Loktantra	1	547198	1080	6060.1	135	4	34	485	3103
IR64 / Sugandha-1	1	918097	1192.6	8447.6	130	4	33	467	2884
IR71033 / Indica	1	196775	1268.1	4941.4	144	4	36	566	5668
IR71033 / IR65482	1	1710485	1217.4	9175.6	126	4	32	464	3005
IR71033 / Sunaulo Sugandha	1	860320	926.8	7693.9	119	4	31	422	2213
IR71033 / Anamol Masuli	1	564274	1140.3	6772.7	129	4	33	473	3077
IR71033 / Khumal-4	1	766608	1211.1	6969.1	135	4	34	495	3335
IR71033 / IRBB-60	1	1081328	1426.9	11899.3	132	5	33	476	2930
IR71033 / Loktantra	1	1056748	1133.6	7076.9	134	5	34	486	3184
IR71033 / Sugandha-1	1	950407	1426.9	9689.4	136	4	34	500	3513
IR65482 / Indica	1	198515	880.1	3722.2	123	4	33	447	3248
IR65482 / Sunaulo Sugandha	1	579861	762.2	5650.8	112	4	30	397	2016
IR65482 / Anamol Masuli	1	518921	972.8	5174	123	4	32	453	2788
IR65482 / Khumal-4	1	859746	984.6	5861.5	125	4	33	454	2754
IR65482 / IRBB-60	1	1678591	985.2	8272.2	118	4	31	418	2310
IR65482 / Loktantra	1	1056748	1021.2	6031.7	124	4	32	453	2930
IR65482 / Sugandha-1	1	641013	1021.4	6595.9	124	4	33	445	2714
Sunaulo Sugandha / Indica	1	197566	851.4	3710.9	123	4	33	445	2921
Sunaulo Sugandha / Anamol Masul	1	551345	788.4	4772.2	117	4	31	417	2179
Sunaulo Sugandha / Khumal-4	1	443432	809.5	4572.6	120	4	32	426	2251
Sunaulo Sugandha / IRBB-60	1	635938	948.7	8056.3	118	4	32	414	2049
Sunaulo Sugandha / Loktantra	1	592109	843.2	5257.2	120	4	31	428	2324
Sunaulo Sugandha / Sugandha-1	1	769127	839.4	6281.6	121	4	32	424	2166
Anamol Masuli / Indica	1	166479	905	3590	130	4	34	477	3485
Anamol Masuli / Khumal-4	1	444039	1012.1	5305.8	131	4	33	476	2897
Anamol Masuli / IRBB-60	1	574516	967.1	6186.8	128	4	33	455	2553
Anamol Masuli / Loktantra	1	631897	1176.4	7182.9	130	4	33	472	3115
Anamol Masuli / Sugandha-1	1	522283	1106.2	6658.7	129	4	33	468	2968
Khumal-4 / Indica	1	161878	1062.7	4042.3	141	4	36	530	4450
Khumal-4 / IRBB-60	1	1056483	1058.6	7066.4	130	4	33	465	2704
Khumal-4 / Loktantra	1	689495	1004.5	5389.6	134	5	34	481	2971
Khumal-4 / Sugandha-1	1	508629	1189.1	6447.7	139	5	35	499	3334
IRBB-60 / Indica	1	196261	1230.1	4876.1	145	4	36	561	5283
IRBB-60 / Loktantra	1	1150752	1033.7	6953.8	130	5	34	459	2739
IRBB-60 / Sugandha-1	1	669683	1131	8310.1	135	5	34	475	2673
Loktantra / Indica	1	170048	905.5	3573	133	4	35	480	3541
Loktantra / Sugandha-1	1	490317	1096.2	6456.5	130	4	34	467	3035
Sugandha-1 / Indica	1	220679	1163.6	4455.8	146	4	36	559	5144

Table S5 Summary of KASP assay validation results. Counts are given of the number of unique marker-cross combinations that have been validated (or otherwise), split according to whether or not the markers met our bioinformatics filtering criteria, and between new and existing markers.

**MARKER-LEVEL**						
No. of markers that have produced genotyping results in one or more cross						
	Existing		Novel		Total	
	Validated	Not validated	Validated	Not validated	Validated	Not validated
Markers passing filtering criteria	48	0	30	5	78	5
Markers failing filtering criteria	22	5	9	2	31	7
	Existing		Novel		Total	
	Validated (%)	Not validated (%)	Validated (%)	Not validated (%)	Validated (%)	Not validated (%)
Markers passing filtering criteria	100.00	0.00	85.71	14.29	93.98	6.02
Markers failing filtering criteria	81.48	18.52	81.82	18.18	81.58	18.42
**CROSS-LEVEL**						
No. of unique marker-cross combinations that have produced genotyping results in one or more sample						
	Existing		Novel		Total	
	Validated	Not validated	Validated	Not validated	Validated	Not validated
Markers passing filtering criteria	335	13	59	5	394	18
Markers failing filtering criteria	158	19	43	12	201	31
	Existing		Novel		Total	
	Validated (%)	Not validated (%)	Validated (%)	Not validated (%)	Validated (%)	Not validated (%)
Markers passing filtering criteria	96.26	3.74	92.19	7.81	95.63	4.37
Markers failing filtering criteria	89.27	10.73	78.18	21.82	86.64	13.36
**ASSAY-LEVEL**						
No. of assays of progeny lines that have produced genotyping results						
	Existing		Novel		Total	
	Validated	Not validated	Validated	Not validated	Validated	Not validated
Markers passing filtering criteria	7124	536	5513	1074	12637	1610
Markers failing filtering criteria	3465	535	1712	501	5177	1036
	Existing		Novel		Total	
	Validated (%)	Not validated (%)	Validated (%)	Not validated (%)	Validated (%)	Not validated (%)
Markers passing filtering criteria	93.00	7.00	83.70	16.30	88.70	11.30
Markers failing filtering criteria	86.63	13.38	77.36	22.64	83.33	16.67

Table S6 Details of marker-cross combinations tested for validation of 46 new and 75 existing KASP assays (N.B. some failed assays may be due to lack of polymorphism.)

ID	Cross tested	Successful assays	Failed assays	Marker-cross combination validated?	Met filtering criteria?
novel_01	Loktantra_x_Sunaulo_Sugandha	187	3	Yes	Yes
novel_02	Loktantra_x_Sunaulo_Sugandha	186	4	Yes	Yes
novel_03	Loktantra_x_Sunaulo_Sugandha	187	3	Yes	Yes
novel_03	IR64_x_Anamol_Masuli	2	0	Yes	Yes
novel_03	IR64_x_Sugandha-1	38	0	Yes	Yes
novel_03	IR65482_x_Anamol_Masuli	19	0	Yes	Yes
novel_03	IR65482_x_Sugandha-1	19	0	Yes	Yes
novel_03	IR71033_x_Khumal-4	11	0	Yes	Yes
novel_03	IR71033_x_Sunaulo_Sugandha	30	0	Yes	Yes
novel_03	IRBB-60_x_Sunaulo_Sugandha	16	0	Yes	Yes
novel_03	Khumal-4_x_IRBB-60	7	0	Yes	Yes
novel_03	Loktantra_x_Sunaulo_Sugandha	82	2	Yes	Yes
novel_04	IR64_x_Sunaulo_Sugandha	20	0	Yes	Yes
novel_04	IR65482_x_Sunaulo_Sugandha	23	0	Yes	Yes
novel_04	IR71033_x_Sunaulo_Sugandha	31	0	Yes	Yes
novel_04	Loktantra_x_Sunaulo_Sugandha	247	8	Yes	Yes
novel_05	Loktantra_x_Sunaulo_Sugandha	190	0	Yes	Yes
novel_06	Loktantra_x_Sunaulo_Sugandha	181	9	Yes	Yes
novel_07	IRBB-60_x_Sunaulo_Sugandha	16	0	Yes	Yes
novel_07	Khumal-4_x_IRBB-60	7	0	Yes	Yes
novel_08	Khumal-4_x_IRBB-60	6	1	Yes	Yes
novel_09	IRBB-60_x_Sunaulo_Sugandha	16	0	Yes	No
novel_09	Khumal-4_x_IRBB-60	7	0	Yes	No
novel_10	IRBB-60_x_Sunaulo_Sugandha	16	0	Yes	No
novel_10	Khumal-4_x_IRBB-60	7	0	Yes	No
novel_11	Loktantra_x_Sunaulo_Sugandha	189	1	Yes	Yes
novel_12	Loktantra_x_Sunaulo_Sugandha	189	1	Yes	Yes
novel_13	IR71033_x_Anamol_Masuli	1	0	Yes	Yes
novel_13	IR71033_x_Khumal-4	9	2	Yes	Yes
novel_13	IR71033_x_Sugandha-1	41	1	Yes	Yes
novel_13	IR71033_x_Sunaulo_Sugandha	29	1	Yes	Yes
novel_14	IR65482_x_Khumal-4	15	3	Yes	No
novel_14	IR65482_x_Sugandha-1	18	1	Yes	No
novel_14	IR65482_x_Sunaulo_Sugandha	21	2	Yes	No
novel_15	IR65482_x_Anamol_Masuli	17	0	Yes	No
novel_15	IR65482_x_Sugandha-1	19	0	Yes	No
novel_15	IR65482_x_Sunaulo_Sugandha	17	0	Yes	No
novel_15	Loktantra_x_Sunaulo_Sugandha	56	0	Yes	No
novel_15	IR65482_x_Anamol_Masuli	0	2	No	No
novel_15	IR65482_x_Khumal-4	0	18	No	No

ID	Cross tested	Successful assays	Failed assays	Marker-cross combination validated?	Met filtering criteria?
novel_15	IR65482_x_Sunaulo_Sugandha	0	6	No	No
novel_16	IR65482_x_Anamol_Masuli	19	0	Yes	No
novel_16	IR65482_x_Khumal-4	12	6	Yes	No
novel_16	IR65482_x_Sugandha-1	19	0	Yes	No
novel_16	IR65482_x_Sunaulo_Sugandha	23	0	Yes	No
novel_16	Loktantra_x_Sunaulo_Sugandha	266	8	Yes	No
novel_17	IR65482_x_Anamol_Masuli	19	0	Yes	No
novel_17	IR65482_x_Khumal-4	12	6	Yes	No
novel_17	IR65482_x_Sugandha-1	19	0	Yes	No
novel_17	IR65482_x_Sunaulo_Sugandha	15	8	Yes	No
novel_17	Loktantra_x_Sunaulo_Sugandha	162	112	Yes	No
novel_18	Loktantra_x_Sunaulo_Sugandha	131	59	Yes	Yes
novel_19	IR64_x_Anamol_Masuli	2	0	Yes	No
novel_19	IR64_x_Khumal-4	24	2	Yes	No
novel_19	IR64_x_Sugandha-1	38	0	Yes	No
novel_19	IR65482_x_Sunaulo_Sugandha	23	0	Yes	No
novel_19	IR71033_x_Sunaulo_Sugandha	30	0	Yes	No
novel_19	IRBB-60_x_Sunaulo_Sugandha	16	0	Yes	No
novel_19	Loktantra_x_Sunaulo_Sugandha	272	2	Yes	No
novel_20	IR64_x_Anamol_Masuli	1	1	Yes	No
novel_20	IR64_x_Sugandha-1	38	0	Yes	No
novel_20	IR65482_x_Anamol_Masuli	19	0	Yes	No
novel_20	IR65482_x_Sugandha-1	18	1	Yes	No
novel_20	IR71033_x_Anamol_Masuli	1	0	Yes	No
novel_20	IR71033_x_Sugandha-1	36	6	Yes	No
novel_20	Loktantra_x_Sunaulo_Sugandha	81	3	Yes	No
novel_21	Loktantra_x_Sunaulo_Sugandha	82	2	Yes	Yes
novel_22	IR64_x_Anamol_Masuli	2	0	Yes	No
novel_22	IR64_x_Khumal-4	23	3	Yes	No
novel_22	IR65482_x_Anamol_Masuli	19	0	Yes	No
novel_22	IR65482_x_Khumal-4	15	3	Yes	No
novel_22	IR71033_x_Anamol_Masuli	1	0	Yes	No
novel_22	IR71033_x_Khumal-4	9	2	Yes	No
novel_22	Khumal-4_x_IRBB-60	7	0	Yes	No
novel_22	Loktantra_x_Sunaulo_Sugandha	262	12	Yes	No
novel_23	IR64_x_Anamol_Masuli	2	0	Yes	Yes
novel_23	IR64_x_Khumal-4	23	3	Yes	Yes
novel_23	IR65482_x_Anamol_Masuli	19	0	Yes	Yes
novel_23	IR65482_x_Khumal-4	13	5	Yes	Yes
novel_23	IR71033_x_Anamol_Masuli	1	0	Yes	Yes
novel_23	IR71033_x_Khumal-4	9	2	Yes	Yes
novel_23	Khumal-4_x_IRBB-60	6	1	Yes	Yes
novel_23	Loktantra_x_Sunaulo_Sugandha	81	3	Yes	Yes

ID	Cross tested	Successful assays	Failed assays	Marker-cross combination validated?	Met filtering criteria?
novel_24	Loktantra_x_Sunaulo_Sugandha	249	6	Yes	Yes
novel_25	Loktantra_x_Sunaulo_Sugandha	188	2	Yes	Yes
novel_26	Loktantra_x_Sunaulo_Sugandha	186	4	Yes	Yes
novel_27	Loktantra_x_Sunaulo_Sugandha	189	1	Yes	Yes
novel_28	Loktantra_x_Sunaulo_Sugandha	187	3	Yes	Yes
novel_29	Loktantra_x_Sunaulo_Sugandha	184	6	Yes	Yes
novel_30	IR65482_x_Khumal-4	13	5	Yes	Yes
novel_30	IR65482_x_Sunaulo_Sugandha	23	0	Yes	Yes
novel_30	Khumal-4_x_IRBB-60	7	0	Yes	Yes
novel_30	Loktantra_x_Sunaulo_Sugandha	432	13	Yes	Yes
novel_31	Loktantra_x_Sunaulo_Sugandha	189	1	Yes	Yes
novel_32	Loktantra_x_Sunaulo_Sugandha	186	4	Yes	Yes
novel_33	Loktantra_x_Sunaulo_Sugandha	189	1	Yes	Yes
novel_34	Loktantra_x_Sunaulo_Sugandha	267	7	Yes	Yes
novel_35	Loktantra_x_Sunaulo_Sugandha	26	58	Yes	Yes
novel_36	IR64_x_Khumal-4	21	2	Yes	Yes
novel_36	IR64_x_Sunaulo_Sugandha	20	0	Yes	Yes
novel_36	IR71033_x_Khumal-4	7	2	Yes	Yes
novel_36	IR71033_x_Sunaulo_Sugandha	31	0	Yes	Yes
novel_37	Loktantra_x_Sunaulo_Sugandha	267	7	Yes	Yes
novel_38	Loktantra_x_Sunaulo_Sugandha	187	3	Yes	Yes
novel_39	Loktantra_x_Sunaulo_Sugandha	135	55	Yes	Yes
novel_40	IR64_x_Khumal-4	0	23	No	Yes
novel_41	Loktantra_x_Sunaulo_Sugandha	0	190	No	Yes
novel_42	Loktantra_x_Sunaulo_Sugandha	0	190	No	Yes
novel_43	Loktantra_x_Sunaulo_Sugandha	0	190	No	Yes
novel_44	Loktantra_x_Sunaulo_Sugandha	0	190	No	Yes
novel_45	IR65482_x_Anamol_Masuli	0	2	No	No
novel_45	IR65482_x_Khumal-4	0	18	No	No
novel_45	IR65482_x_Sunaulo_Sugandha	0	6	No	No
novel_45	IR65482_x_Anamol_Masuli	0	2	No	No
novel_45	IR65482_x_Khumal-4	0	18	No	No
novel_45	IR65482_x_Sunaulo_Sugandha	0	6	No	No
novel_45	Loktantra_x_Sunaulo_Sugandha	0	218	No	No
novel_46	IR65482_x_Anamol_Masuli	0	2	No	No
novel_46	IR71033_x_Khumal-4	0	9	No	No
novel_46	IR71033_x_Sunaulo_Sugandha	0	4	No	No
novel_46	Khumal-4_x_IRBB-60	0	7	No	No
novel_46	Loktantra_x_Sunaulo_Sugandha	0	28	No	No
id10001497	IR64_x_Khumal-4	25	1	Yes	No
id10001497	IR64_x_Sunaulo_Sugandha	19	1	Yes	No
id10001497	IR65482_x_Khumal-4	15	3	Yes	No
id10001497	IR65482_x_Sunaulo_Sugandha	21	2	Yes	No

ID	Cross tested	Successful assays	Failed assays	Marker-cross combination validated?	Met filtering criteria?
id10001497	IR71033_x_Anamol_Masuli	1	0	Yes	No
id10001497	IR71033_x_Sugandha-1	8	34	Yes	No
id10001497	Loktantra_x_Sunaulo_Sugandha	77	7	Yes	No
id10003895	IR64_x_Sunaulo_Sugandha	20	0	Yes	No
id10003895	IR65482_x_Sunaulo_Sugandha	23	0	Yes	No
id10003895	IR71033_x_Sunaulo_Sugandha	28	2	Yes	No
id10003895	IRBB-60_x_Sunaulo_Sugandha	16	0	Yes	No
id10003895	Loktantra_x_Sunaulo_Sugandha	79	5	Yes	No
id10007241	IR64_x_Khumal-4	22	4	Yes	No
id10007241	IR64_x_Sugandha-1	38	0	Yes	No
id10007241	IR64_x_Sunaulo_Sugandha	20	0	Yes	No
id10007241	IR65482_x_Khumal-4	12	6	Yes	No
id10007241	IR65482_x_Sugandha-1	19	0	Yes	No
id10007241	IR65482_x_Sunaulo_Sugandha	23	0	Yes	No
id10007241	IR71033_x_Anamol_Masuli	1	0	Yes	No
id1001235	IR64_x_Anamol_Masuli	2	0	Yes	Yes
id1001235	IR64_x_Sugandha-1	38	0	Yes	Yes
id1001235	IR65482_x_Khumal-4	15	3	Yes	Yes
id1001235	IR65482_x_Sunaulo_Sugandha	23	0	Yes	Yes
id1001235	IR71033_x_Khumal-4	10	1	Yes	Yes
id1001235	IR71033_x_Sunaulo_Sugandha	30	0	Yes	Yes
id1001235	IRBB-60_x_Sunaulo_Sugandha	16	0	Yes	Yes
id1001235	Khumal-4_x_IRBB-60	7	0	Yes	Yes
id1001235	Loktantra_x_Sunaulo_Sugandha	83	1	Yes	Yes
id1019286	IR64_x_Anamol_Masuli	2	0	Yes	Yes
id1019286	IR64_x_Khumal-4	25	1	Yes	Yes
id1019286	IR64_x_Sunaulo_Sugandha	20	0	Yes	Yes
id1019286	IR65482_x_Anamol_Masuli	19	0	Yes	Yes
id1019286	IR65482_x_Khumal-4	16	2	Yes	Yes
id1019286	IR65482_x_Sunaulo_Sugandha	23	0	Yes	Yes
id1019286	IR71033_x_Anamol_Masuli	1	0	Yes	Yes
id1019286	IR71033_x_Khumal-4	9	2	Yes	Yes
id1019286	IR71033_x_Sunaulo_Sugandha	30	0	Yes	Yes
id1019286	IRBB-60_x_Sunaulo_Sugandha	16	0	Yes	Yes
id1019286	Khumal-4_x_IRBB-60	7	0	Yes	Yes
id1019286	Loktantra_x_Sunaulo_Sugandha	83	1	Yes	Yes
id11006360	IR64_x_Khumal-4	20	6	Yes	Yes
id11006360	IR65482_x_Anamol_Masuli	19	0	Yes	Yes
id11006360	IR65482_x_Sugandha-1	19	0	Yes	Yes
id11006360	IR65482_x_Sunaulo_Sugandha	23	0	Yes	Yes
id11006360	IR71033_x_Anamol_Masuli	1	0	Yes	Yes
id11006360	IR71033_x_Sugandha-1	41	1	Yes	Yes
id11006360	IR71033_x_Sunaulo_Sugandha	28	2	Yes	Yes

ID	Cross tested	Successful assays	Failed assays	Marker-cross combination validated?	Met filtering criteria?
id11006360	Khumal-4_x_IRBB-60	5	2	Yes	Yes
id11006360	Loktantra_x_Sunaulo_Sugandha	81	3	Yes	Yes
id11009699	IR64_x_Anamol_Masuli	2	0	Yes	Yes
id11009699	IR64_x_Sugandha-1	38	0	Yes	Yes
id11009699	IR64_x_Sunaulo_Sugandha	20	0	Yes	Yes
id11009699	IR65482_x_Khumal-4	14	4	Yes	Yes
id11009699	IR71033_x_Anamol_Masuli	1	0	Yes	Yes
id11009699	IR71033_x_Sugandha-1	41	1	Yes	Yes
id11009699	IR71033_x_Sunaulo_Sugandha	30	0	Yes	Yes
id11009699	Khumal-4_x_IRBB-60	7	0	Yes	Yes
id12001390	IR64_x_Sunaulo_Sugandha	20	0	Yes	No
id12001390	IR65482_x_Sunaulo_Sugandha	23	0	Yes	No
id12001390	IR71033_x_Sunaulo_Sugandha	29	1	Yes	No
id12001390	IRBB-60_x_Sunaulo_Sugandha	16	0	Yes	No
id12001390	Loktantra_x_Sunaulo_Sugandha	83	1	Yes	No
id12003239	IR64_x_Anamol_Masuli	2	0	Yes	Yes
id12003239	IR65482_x_Sugandha-1	18	1	Yes	Yes
id12003239	IR65482_x_Sunaulo_Sugandha	17	0	Yes	Yes
id12003239	IR71033_x_Khumal-4	2	0	Yes	Yes
id12003239	IR71033_x_Sugandha-1	41	1	Yes	Yes
id12003239	IR71033_x_Sunaulo_Sugandha	25	1	Yes	Yes
id12008557	IR65482_x_Anamol_Masuli	19	0	Yes	Yes
id12008557	IR65482_x_Khumal-4	13	5	Yes	Yes
id12008557	IR65482_x_Sugandha-1	19	0	Yes	Yes
id12008557	IR65482_x_Sunaulo_Sugandha	23	0	Yes	Yes
id12008557	Loktantra_x_Sunaulo_Sugandha	83	1	Yes	Yes
id12010012	IR64_x_Anamol_Masuli	2	0	Yes	Yes
id12010012	IR64_x_Khumal-4	19	7	Yes	Yes
id12010012	IR64_x_Sugandha-1	38	0	Yes	Yes
id12010012	IR64_x_Sunaulo_Sugandha	20	0	Yes	Yes
id12010012	IR71033_x_Anamol_Masuli	1	0	Yes	Yes
id12010012	IR71033_x_Khumal-4	8	3	Yes	Yes
id12010012	IR71033_x_Sugandha-1	41	1	Yes	Yes
id12010012	IR71033_x_Sunaulo_Sugandha	29	1	Yes	Yes
id2003908	IR64_x_Khumal-4	23	3	Yes	Yes
id2003908	IR64_x_Sunaulo_Sugandha	19	1	Yes	Yes
id2003908	IR65482_x_Khumal-4	12	6	Yes	Yes
id2003908	IR65482_x_Sunaulo_Sugandha	23	0	Yes	Yes
id2003908	IR71033_x_Khumal-4	9	2	Yes	Yes
id2003908	IR71033_x_Sunaulo_Sugandha	29	1	Yes	Yes
id2003908	Loktantra_x_Sunaulo_Sugandha	83	1	Yes	Yes
id2013887	IR64_x_Khumal-4	25	1	Yes	Yes
id2013887	IR64_x_Sunaulo_Sugandha	20	0	Yes	Yes

ID	Cross tested	Successful assays	Failed assays	Marker-cross combination validated?	Met filtering criteria?
id2013887	IR65482_x_Anamol_Masuli	19	0	Yes	Yes
id2013887	IR65482_x_Sugandha-1	19	0	Yes	Yes
id2013887	IR71033_x_Khumal-4	10	1	Yes	Yes
id2013887	IR71033_x_Sunaulo_Sugandha	29	1	Yes	Yes
id2015384	IR64_x_Anamol_Masuli	2	0	Yes	Yes
id2015384	IR64_x_Sugandha-1	38	0	Yes	Yes
id2015384	IR65482_x_Khumal-4	14	4	Yes	Yes
id2015384	IR65482_x_Sunaulo_Sugandha	23	0	Yes	Yes
id2015384	IR71033_x_Anamol_Masuli	1	0	Yes	Yes
id2015384	IR71033_x_Sugandha-1	42	0	Yes	Yes
id2015384	IRBB-60_x_Sunaulo_Sugandha	16	0	Yes	Yes
id2015384	Khumal-4_x_IRBB-60	5	2	Yes	Yes
id3004086	IR64_x_Sugandha-1	38	0	Yes	Yes
id3004086	IR65482_x_Anamol_Masuli	19	0	Yes	Yes
id3004086	IR65482_x_Khumal-4	12	6	Yes	Yes
id3004086	IR65482_x_Sunaulo_Sugandha	23	0	Yes	Yes
id3004086	IR71033_x_Anamol_Masuli	1	0	Yes	Yes
id3004086	IR71033_x_Khumal-4	9	2	Yes	Yes
id3004086	IR71033_x_Sunaulo_Sugandha	30	0	Yes	Yes
id3004086	Loktantra_x_Sunaulo_Sugandha	83	1	Yes	Yes
id3004859	IR64_x_Anamol_Masuli	2	0	Yes	Yes
id3004859	IR64_x_Sugandha-1	38	0	Yes	Yes
id3004859	IR65482_x_Sunaulo_Sugandha	17	0	Yes	Yes
id3004859	IR71033_x_Anamol_Masuli	1	0	Yes	Yes
id3004859	IR71033_x_Sugandha-1	42	0	Yes	Yes
id3004859	Loktantra_x_Sunaulo_Sugandha	56	0	Yes	Yes
id3008123	IR64_x_Khumal-4	24	2	Yes	Yes
id3008123	IR64_x_Sunaulo_Sugandha	20	0	Yes	Yes
id3008123	IR65482_x_Khumal-4	12	6	Yes	Yes
id3008123	IR65482_x_Sunaulo_Sugandha	23	0	Yes	Yes
id3008123	IR71033_x_Khumal-4	9	2	Yes	Yes
id3008123	IR71033_x_Sunaulo_Sugandha	29	1	Yes	Yes
id3008123	IRBB-60_x_Sunaulo_Sugandha	16	0	Yes	Yes
id3008123	Khumal-4_x_IRBB-60	7	0	Yes	Yes
id3008123	Loktantra_x_Sunaulo_Sugandha	83	1	Yes	Yes
id3010173	IR64_x_Anamol_Masuli	2	0	Yes	Yes
id3010173	IR64_x_Sunaulo_Sugandha	20	0	Yes	Yes
id3010173	IR65482_x_Anamol_Masuli	19	0	Yes	Yes
id3010173	IR65482_x_Sunaulo_Sugandha	23	0	Yes	Yes
id3010173	IR71033_x_Anamol_Masuli	1	0	Yes	Yes
id3010173	IR71033_x_Sunaulo_Sugandha	29	1	Yes	Yes
id3010173	IRBB-60_x_Sunaulo_Sugandha	16	0	Yes	Yes
id4007959	IR64_x_Anamol_Masuli	2	0	Yes	Yes

ID	Cross tested	Successful assays	Failed assays	Marker-cross combination validated?	Met filtering criteria?
id4007959	IR64_x_Khumal-4	23	3	Yes	Yes
id4007959	IR65482_x_Anamol_Masuli	18	1	Yes	Yes
id4007959	IR65482_x_Khumal-4	12	6	Yes	Yes
id4007959	IR71033_x_Anamol_Masuli	1	0	Yes	Yes
id4007959	IR71033_x_Khumal-4	9	2	Yes	Yes
id4007959	Khumal-4_x_IRBB-60	7	0	Yes	Yes
id4007959	Loktantra_x_Sunaulo_Sugandha	83	1	Yes	Yes
id4009710	IR64_x_Sunaulo_Sugandha	20	0	Yes	Yes
id4009710	IR65482_x_Sunaulo_Sugandha	23	0	Yes	Yes
id4009710	IR71033_x_Sunaulo_Sugandha	29	1	Yes	Yes
id4009710	IRBB-60_x_Sunaulo_Sugandha	16	0	Yes	Yes
id4009710	Loktantra_x_Sunaulo_Sugandha	83	1	Yes	Yes
id5000759	IR64_x_Anamol_Masuli	2	0	Yes	Yes
id5000759	IR64_x_Sunaulo_Sugandha	20	0	Yes	Yes
id5000759	IR65482_x_Anamol_Masuli	19	0	Yes	Yes
id5000759	IR65482_x_Sunaulo_Sugandha	23	0	Yes	Yes
id5000759	IR71033_x_Khumal-4	9	2	Yes	Yes
id5000759	IR71033_x_Sugandha-1	42	0	Yes	Yes
id5000759	IRBB-60_x_Sunaulo_Sugandha	16	0	Yes	Yes
id5000759	Loktantra_x_Sunaulo_Sugandha	83	1	Yes	Yes
id5006768	IR64_x_Khumal-4	24	2	Yes	No
id5006768	IR64_x_Sunaulo_Sugandha	20	0	Yes	No
id5006768	IR65482_x_Anamol_Masuli	18	1	Yes	No
id5006768	IR65482_x_Sugandha-1	18	1	Yes	No
id5006768	IR71033_x_Khumal-4	9	2	Yes	No
id5006768	IR71033_x_Sunaulo_Sugandha	30	0	Yes	No
id5006768	IRBB-60_x_Sunaulo_Sugandha	16	0	Yes	No
id5006768	Khumal-4_x_IRBB-60	7	0	Yes	No
id5006768	Loktantra_x_Sunaulo_Sugandha	83	1	Yes	No
id5008574	IR64_x_Khumal-4	21	5	Yes	Yes
id5008574	IR64_x_Sugandha-1	38	0	Yes	Yes
id5008574	IR64_x_Sunaulo_Sugandha	20	0	Yes	Yes
id5008574	IR65482_x_Khumal-4	12	6	Yes	Yes
id5008574	IR65482_x_Sugandha-1	19	0	Yes	Yes
id5008574	IR65482_x_Sunaulo_Sugandha	23	0	Yes	Yes
id5008574	IR71033_x_Anamol_Masuli	1	0	Yes	Yes
id6001041	IR64_x_Anamol_Masuli	2	0	Yes	Yes
id6001041	IR64_x_Sugandha-1	38	0	Yes	Yes
id6001041	IR65482_x_Khumal-4	15	3	Yes	Yes
id6001041	IR65482_x_Sunaulo_Sugandha	23	0	Yes	Yes
id6001041	IR71033_x_Anamol_Masuli	1	0	Yes	Yes
id6001041	IR71033_x_Sugandha-1	41	1	Yes	Yes
id6001041	Loktantra_x_Sunaulo_Sugandha	84	0	Yes	Yes

ID	Cross tested	Successful assays	Failed assays	Marker-cross combination validated?	Met filtering criteria?
id6001266	IR71033_x_Anamol_Masuli	0	1	No	No
id6001266	IR71033_x_Khumal-4	0	2	No	No
id6001266	IR71033_x_Sugandha-1	0	42	No	No
id6001266	IR71033_x_Sunaulo_Sugandha	0	26	No	No
id6003378	IR64_x_Anamol_Masuli	2	0	Yes	No
id6003378	IR64_x_Khumal-4	3	0	Yes	No
id6003378	IR64_x_Sugandha-1	37	1	Yes	No
id6003378	IR64_x_Sunaulo_Sugandha	13	0	Yes	No
id6012115	IR64_x_Sunaulo_Sugandha	7	0	Yes	Yes
id6012115	IR65482_x_Sunaulo_Sugandha	6	0	Yes	Yes
id6012115	IR71033_x_Sunaulo_Sugandha	4	0	Yes	Yes
id6012115	Loktantra_x_Sunaulo_Sugandha	27	1	Yes	Yes
id7000500	IR64_x_Khumal-4	23	3	Yes	Yes
id7000500	IR65482_x_Khumal-4	12	6	Yes	Yes
id7000500	IR71033_x_Khumal-4	9	2	Yes	Yes
id7000500	IRBB-60_x_Sunaulo_Sugandha	16	0	Yes	Yes
id7004596	IR64_x_Sunaulo_Sugandha	20	0	Yes	Yes
id7004596	IR65482_x_Sunaulo_Sugandha	23	0	Yes	Yes
id7004596	IR71033_x_Sunaulo_Sugandha	29	1	Yes	Yes
id7004596	IRBB-60_x_Sunaulo_Sugandha	16	0	Yes	Yes
id7004596	Loktantra_x_Sunaulo_Sugandha	81	3	Yes	Yes
id7005449	IR64_x_Anamol_Masuli	2	0	Yes	Yes
id7005449	IR64_x_Khumal-4	22	4	Yes	Yes
id7005449	IR64_x_Sugandha-1	38	0	Yes	Yes
id7005449	IR64_x_Sunaulo_Sugandha	20	0	Yes	Yes
id7005449	IR65482_x_Anamol_Masuli	19	0	Yes	Yes
id7005449	IR65482_x_Khumal-4	12	6	Yes	Yes
id7005449	IR65482_x_Sugandha-1	19	0	Yes	Yes
id7005449	IR65482_x_Sunaulo_Sugandha	23	0	Yes	Yes
id7005449	IR71033_x_Anamol_Masuli	1	0	Yes	Yes
id7005449	IR71033_x_Khumal-4	9	2	Yes	Yes
id7005449	IR71033_x_Sugandha-1	41	1	Yes	Yes
id7005449	IR71033_x_Sunaulo_Sugandha	29	1	Yes	Yes
id7005449	IRBB-60_x_Sunaulo_Sugandha	16	0	Yes	Yes
id7005449	Khumal-4_x_IRBB-60	6	1	Yes	Yes
id8000011	IR64_x_Anamol_Masuli	2	0	Yes	Yes
id8000011	IR64_x_Sugandha-1	38	0	Yes	Yes
id8000011	IR65482_x_Anamol_Masuli	17	0	Yes	Yes
id8000011	IR65482_x_Sugandha-1	18	1	Yes	Yes
id8000011	IR71033_x_Khumal-4	2	0	Yes	Yes
id8000011	IR71033_x_Sunaulo_Sugandha	25	1	Yes	Yes
id8000011	Loktantra_x_Sunaulo_Sugandha	55	1	Yes	Yes
id8002306	IR64_x_Anamol_Masuli	2	0	Yes	Yes

ID	Cross tested	Successful assays	Failed assays	Marker-cross combination validated?	Met filtering criteria?
id8002306	IR64_x_Khumal-4	2	1	Yes	Yes
id8002306	IR64_x_Sugandha-1	37	1	Yes	Yes
id8002306	IR65482_x_Anamol_Masuli	17	0	Yes	Yes
id8002306	IR65482_x_Sugandha-1	19	0	Yes	Yes
id8002306	IR71033_x_Anamol_Masuli	1	0	Yes	Yes
id8002306	IR71033_x_Khumal-4	2	0	Yes	Yes
id8002306	IR71033_x_Sugandha-1	42	0	Yes	Yes
id8003309	IR64_x_Sugandha-1	38	0	Yes	Yes
id8003309	IR64_x_Sunaulo_Sugandha	13	0	Yes	Yes
id8003309	IR65482_x_Sugandha-1	19	0	Yes	Yes
id8003309	IR65482_x_Sunaulo_Sugandha	17	0	Yes	Yes
id8003309	IR71033_x_Sugandha-1	42	0	Yes	Yes
id8003309	IR71033_x_Sunaulo_Sugandha	26	0	Yes	Yes
id8003309	IRBB-60_x_Sunaulo_Sugandha	16	0	Yes	Yes
id8003309	Loktantra_x_Sunaulo_Sugandha	56	0	Yes	Yes
id8005221	IR64_x_Anamol_Masuli	2	0	Yes	No
id8005221	IR64_x_Sugandha-1	38	0	Yes	No
id8005221	IR64_x_Sunaulo_Sugandha	13	0	Yes	No
id8005221	IR65482_x_Anamol_Masuli	17	0	Yes	No
id8005221	IR65482_x_Sugandha-1	19	0	Yes	No
id8005221	IR65482_x_Sunaulo_Sugandha	17	0	Yes	No
id8005221	IR71033_x_Anamol_Masuli	1	0	Yes	No
id8005221	IR71033_x_Sugandha-1	42	0	Yes	No
id8005221	IR71033_x_Sunaulo_Sugandha	26	0	Yes	No
id8005221	IRBB-60_x_Sunaulo_Sugandha	16	0	Yes	No
id8005221	Loktantra_x_Sunaulo_Sugandha	56	0	Yes	No
id8005359	IR64_x_Anamol_Masuli	2	0	Yes	Yes
id8005359	IR64_x_Khumal-4	23	3	Yes	Yes
id8005359	IR64_x_Sunaulo_Sugandha	20	0	Yes	Yes
id8005359	IR65482_x_Anamol_Masuli	19	0	Yes	Yes
id8005359	IR65482_x_Khumal-4	12	6	Yes	Yes
id8005359	IR65482_x_Sunaulo_Sugandha	23	0	Yes	Yes
id8005359	IR71033_x_Anamol_Masuli	1	0	Yes	Yes
id8005359	IR71033_x_Khumal-4	9	2	Yes	Yes
id8005359	IR71033_x_Sunaulo_Sugandha	30	0	Yes	Yes
id8005359	IRBB-60_x_Sunaulo_Sugandha	16	0	Yes	Yes
id8005359	Khumal-4_x_IRBB-60	7	0	Yes	Yes
id8005359	Loktantra_x_Sunaulo_Sugandha	82	2	Yes	Yes
id9002046	IR64_x_Sunaulo_Sugandha	20	0	Yes	No
id9002046	IR65482_x_Sunaulo_Sugandha	23	0	Yes	No
id9002046	IR71033_x_Sunaulo_Sugandha	29	1	Yes	No
id9002046	IRBB-60_x_Sunaulo_Sugandha	16	0	Yes	No
id9002046	Loktantra_x_Sunaulo_Sugandha	83	1	Yes	No

ID	Cross tested	Successful assays	Failed assays	Marker-cross combination validated?	Met filtering criteria?
id9002497	IR64_x_Anamol_Masuli	2	0	Yes	No
id9002497	IR64_x_Khumal-4	24	2	Yes	No
id9002497	IR65482_x_Sugandha-1	19	0	Yes	No
id9002497	IR65482_x_Sunaulo_Sugandha	23	0	Yes	No
id9002497	IR71033_x_Anamol_Masuli	1	0	Yes	No
id9002497	IR71033_x_Khumal-4	9	2	Yes	No
id9002497	Khumal-4_x_IRBB-60	6	1	Yes	No
id9002755	IR64_x_Anamol_Masuli	2	0	Yes	Yes
id9002755	IR64_x_Sunaulo_Sugandha	13	0	Yes	Yes
id9002755	IR65482_x_Anamol_Masuli	17	0	Yes	Yes
id9002755	IR65482_x_Sunaulo_Sugandha	17	0	Yes	Yes
id9002755	IR71033_x_Anamol_Masuli	1	0	Yes	Yes
id9002755	IR71033_x_Sunaulo_Sugandha	25	1	Yes	Yes
id9002755	IRBB-60_x_Sunaulo_Sugandha	16	0	Yes	Yes
id9006881	IR71033_x_Anamol_Masuli	1	0	Yes	Yes
id9006881	IR71033_x_Khumal-4	10	1	Yes	Yes
id9006881	IR71033_x_Sugandha-1	42	0	Yes	Yes
id9006881	IR71033_x_Sunaulo_Sugandha	29	1	Yes	Yes
K_id10005402	IR64_x_Khumal-4	0	23	No	No
K_id10005402	IR65482_x_Khumal-4	0	18	No	No
K_id10005402	IR71033_x_Khumal-4	0	9	No	No
K_id10005402	Khumal-4_x_IRBB-60	0	7	No	No
K_id10005402	Loktantra_x_Sunaulo_Sugandha	0	28	No	No
K_id10005853	IR64_x_Sunaulo_Sugandha	13	0	Yes	No
K_id10005853	IR65482_x_Anamol_Masuli	17	0	Yes	No
K_id10005853	IR65482_x_Sugandha-1	18	1	Yes	No
K_id10005853	IR71033_x_Sunaulo_Sugandha	25	1	Yes	No
K_id10005853	Loktantra_x_Sunaulo_Sugandha	56	0	Yes	No
K_id1003559	IR64_x_Sugandha-1	38	0	Yes	No
K_id1003559	IR64_x_Sunaulo_Sugandha	12	1	Yes	No
K_id1003559	IR65482_x_Sugandha-1	16	3	Yes	No
K_id1003559	IR65482_x_Sunaulo_Sugandha	16	1	Yes	No
K_id1003559	IR71033_x_Sugandha-1	39	3	Yes	No
K_id1003559	IR71033_x_Sunaulo_Sugandha	26	0	Yes	No
K_id1003559	IRBB-60_x_Sunaulo_Sugandha	16	0	Yes	No
K_id1003559	Loktantra_x_Sunaulo_Sugandha	53	3	Yes	No
K_id1004591	IR64_x_Anamol_Masuli	2	0	Yes	Yes
K_id1004591	IR64_x_Khumal-4	3	0	Yes	Yes
K_id1004591	IR64_x_Sugandha-1	38	0	Yes	Yes
K_id1004591	IR64_x_Sunaulo_Sugandha	13	0	Yes	Yes
K_id1005125	IR64_x_Anamol_Masuli	2	0	Yes	Yes
K_id1005125	IR64_x_Khumal-4	23	3	Yes	Yes
K_id1005125	IR64_x_Sugandha-1	38	0	Yes	Yes

ID	Cross tested	Successful assays	Failed assays	Marker-cross combination validated?	Met filtering criteria?
K_id1005125	IR64_x_Sunaulo_Sugandha	20	0	Yes	Yes
K_id1005125	IR65482_x_Anamol_Masuli	19	0	Yes	Yes
K_id1005125	IR65482_x_Khumal-4	12	6	Yes	Yes
K_id1005125	IR65482_x_Sugandha-1	19	0	Yes	Yes
K_id1005125	IR65482_x_Sunaulo_Sugandha	23	0	Yes	Yes
K_id1021259	IR64_x_Anamol_Masuli	2	0	Yes	Yes
K_id1021259	IR64_x_Khumal-4	2	1	Yes	Yes
K_id1021259	IR65482_x_Anamol_Masuli	17	0	Yes	Yes
K_id1021259	IR71033_x_Anamol_Masuli	1	0	Yes	Yes
K_id1021259	IR71033_x_Khumal-4	2	0	Yes	Yes
K_id1022207	IR64_x_Sunaulo_Sugandha	13	0	Yes	Yes
K_id1022207	IR65482_x_Sunaulo_Sugandha	17	0	Yes	Yes
K_id1022207	IR71033_x_Sunaulo_Sugandha	26	0	Yes	Yes
K_id1022207	IRBB-60_x_Sunaulo_Sugandha	16	0	Yes	Yes
K_id1022207	Loktantra_x_Sunaulo_Sugandha	55	1	Yes	Yes
K_id11000858	IR64_x_Khumal-4	23	3	Yes	Yes
K_id11000858	IR64_x_Sugandha-1	38	0	Yes	Yes
K_id11000858	IR65482_x_Khumal-4	12	6	Yes	Yes
K_id11000858	IR65482_x_Sugandha-1	19	0	Yes	Yes
K_id11000858	IR71033_x_Khumal-4	9	2	Yes	Yes
K_id11000858	IR71033_x_Sugandha-1	42	0	Yes	Yes
K_id11000858	Khumal-4_x_IRBB-60	7	0	Yes	Yes
K_id11010811	IR64_x_Anamol_Masuli	0	2	No	No
K_id11010811	IR64_x_Khumal-4	18	8	Yes	No
K_id11010811	IR64_x_Sugandha-1	0	38	No	No
K_id11010811	IR64_x_Sunaulo_Sugandha	7	13	Yes	No
K_id11010811	IRBB-60_x_Sunaulo_Sugandha	0	16	No	No
K_id11010811	Khumal-4_x_IRBB-60	5	2	Yes	No
K_id11010811	Loktantra_x_Sunaulo_Sugandha	27	57	Yes	No
K_id12005592	IR64_x_Anamol_Masuli	2	0	Yes	No
K_id12005592	IR64_x_Khumal-4	2	1	Yes	No
K_id12005592	IR65482_x_Sugandha-1	19	0	Yes	No
K_id12005592	IR65482_x_Sunaulo_Sugandha	17	0	Yes	No
K_id12005592	IR71033_x_Anamol_Masuli	1	0	Yes	No
K_id12005592	IR71033_x_Khumal-4	2	0	Yes	No
K_id12005592	Loktantra_x_Sunaulo_Sugandha	56	0	Yes	No
K_id2000235	IR64_x_Khumal-4	3	0	Yes	No
K_id2000235	IR64_x_Sugandha-1	38	0	Yes	No
K_id2000235	IR64_x_Sunaulo_Sugandha	13	0	Yes	No
K_id2000235	IR65482_x_Anamol_Masuli	17	0	Yes	No
K_id2000235	IR71033_x_Anamol_Masuli	1	0	Yes	No
K_id2000235	Loktantra_x_Sunaulo_Sugandha	56	0	Yes	No
K_id2004711	IR64_x_Sugandha-1	38	0	Yes	Yes

ID	Cross tested	Successful assays	Failed assays	Marker-cross combination validated?	Met filtering criteria?
K_id2004711	IR64_x_Sunaulo_Sugandha	20	0	Yes	Yes
K_id2004711	IR65482_x_Anamol_Masuli	19	0	Yes	Yes
K_id2004711	IR65482_x_Khumal-4	14	4	Yes	Yes
K_id2004711	IR71033_x_Anamol_Masuli	1	0	Yes	Yes
K_id2004711	IR71033_x_Khumal-4	9	2	Yes	Yes
K_id2004711	Khumal-4_x_IRBB-60	7	0	Yes	Yes
K_id3000492	IR64_x_Khumal-4	24	2	Yes	Yes
K_id3000492	IR64_x_Sugandha-1	38	0	Yes	Yes
K_id3000492	IR64_x_Sunaulo_Sugandha	19	1	Yes	Yes
K_id3000492	IR65482_x_Khumal-4	12	6	Yes	Yes
K_id3000492	IR65482_x_Sugandha-1	19	0	Yes	Yes
K_id3000492	IR65482_x_Sunaulo_Sugandha	23	0	Yes	Yes
K_id3000492	IR71033_x_Khumal-4	10	1	Yes	Yes
K_id3000492	IR71033_x_Sugandha-1	42	0	Yes	Yes
K_id3000492	IR71033_x_Sunaulo_Sugandha	29	1	Yes	Yes
K_id3006808	IR64_x_Anamol_Masuli	2	0	Yes	Yes
K_id3006808	IR64_x_Khumal-4	2	1	Yes	Yes
K_id3006808	IR64_x_Sugandha-1	38	0	Yes	Yes
K_id3006808	IR65482_x_Anamol_Masuli	17	0	Yes	Yes
K_id3006808	IR65482_x_Sugandha-1	19	0	Yes	Yes
K_id3006808	IR71033_x_Sunaulo_Sugandha	25	1	Yes	Yes
K_id3006808	Loktantra_x_Sunaulo_Sugandha	55	1	Yes	Yes
K_id3018242	IR64_x_Khumal-4	23	3	Yes	No
K_id3018242	IR64_x_Sugandha-1	38	0	Yes	No
K_id3018242	IR65482_x_Khumal-4	12	6	Yes	No
K_id3018242	IR65482_x_Sugandha-1	19	0	Yes	No
K_id3018242	IR71033_x_Khumal-4	9	2	Yes	No
K_id3018242	IR71033_x_Sugandha-1	42	0	Yes	No
K_id3018242	Khumal-4_x_IRBB-60	7	0	Yes	No
K_id3018242	Loktantra_x_Sunaulo_Sugandha	82	2	Yes	No
K_id4000585	IR64_x_Sugandha-1	38	0	Yes	No
K_id4000585	IR65482_x_Anamol_Masuli	19	0	Yes	No
K_id4000585	IR65482_x_Khumal-4	12	6	Yes	No
K_id4000585	IR65482_x_Sunaulo_Sugandha	23	0	Yes	No
K_id4000585	IR71033_x_Anamol_Masuli	1	0	Yes	No
K_id4000585	IR71033_x_Khumal-4	9	2	Yes	No
K_id4000585	IR71033_x_Sunaulo_Sugandha	29	1	Yes	No
K_id4005120	IR64_x_Sunaulo_Sugandha	13	0	Yes	No
K_id4005120	IR65482_x_Sunaulo_Sugandha	17	0	Yes	No
K_id4005120	IR71033_x_Anamol_Masuli	1	0	Yes	No
K_id4005120	IR71033_x_Khumal-4	2	0	Yes	No
K_id4005120	IR71033_x_Sugandha-1	38	4	Yes	No
K_id4005120	Loktantra_x_Sunaulo_Sugandha	56	0	Yes	No

ID	Cross tested	Successful assays	Failed assays	Marker-cross combination validated?	Met filtering criteria?
K_id4007212	IR64_x_Anamol_Masuli	2	0	Yes	Yes
K_id4007212	IR64_x_Khumal-4	3	0	Yes	Yes
K_id4007212	IR64_x_Sunaulo_Sugandha	13	0	Yes	Yes
K_id4007212	IR65482_x_Anamol_Masuli	17	0	Yes	Yes
K_id4007212	IR65482_x_Sunaulo_Sugandha	17	0	Yes	Yes
K_id4007212	IR71033_x_Anamol_Masuli	1	0	Yes	Yes
K_id4007212	IR71033_x_Khumal-4	2	0	Yes	Yes
K_id4007212	IR71033_x_Sunaulo_Sugandha	25	1	Yes	Yes
K_id4007212	IRBB-60_x_Sunaulo_Sugandha	15	1	Yes	Yes
K_id4008100	IR64_x_Khumal-4	0	3	No	No
K_id4008100	IR71033_x_Khumal-4	0	2	No	No
K_id4010708	IR64_x_Anamol_Masuli	1	1	Yes	No
K_id4010708	IR64_x_Khumal-4	24	2	Yes	No
K_id4010708	IR64_x_Sugandha-1	24	14	Yes	No
K_id4010708	IR64_x_Sunaulo_Sugandha	20	0	Yes	No
K_id4010708	IR71033_x_Anamol_Masuli	1	0	Yes	No
K_id4010708	IR71033_x_Khumal-4	9	2	Yes	No
K_id4010708	IR71033_x_Sugandha-1	34	8	Yes	No
K_id4010708	IR71033_x_Sunaulo_Sugandha	28	2	Yes	No
K_id4010708	IRBB-60_x_Sunaulo_Sugandha	16	0	Yes	No
K_id4010708	Khumal-4_x_IRBB-60	6	1	Yes	No
K_id4012434	IR64_x_Sugandha-1	0	38	No	Yes
K_id4012434	IR64_x_Sunaulo_Sugandha	0	13	No	Yes
K_id4012434	IR65482_x_Sugandha-1	0	19	No	Yes
K_id4012434	IR65482_x_Sunaulo_Sugandha	0	17	No	Yes
K_id4012434	IR71033_x_Sugandha-1	0	42	No	Yes
K_id4012434	IR71033_x_Sunaulo_Sugandha	0	26	No	Yes
K_id4012434	IRBB-60_x_Sunaulo_Sugandha	0	16	No	Yes
K_id4012434	Loktantra_x_Sunaulo_Sugandha	0	56	No	Yes
K_id5000953	IR64_x_Khumal-4	24	2	Yes	Yes
K_id5000953	IR64_x_Sugandha-1	38	0	Yes	Yes
K_id5000953	IR65482_x_Khumal-4	12	6	Yes	Yes
K_id5000953	IR65482_x_Sugandha-1	19	0	Yes	Yes
K_id5000953	IR71033_x_Sunaulo_Sugandha	30	0	Yes	Yes
K_id5000953	IRBB-60_x_Sunaulo_Sugandha	16	0	Yes	Yes
K_id5000953	Loktantra_x_Sunaulo_Sugandha	83	1	Yes	Yes
K_id6002100	IR64_x_Anamol_Masuli	2	0	Yes	No
K_id6002100	IR64_x_Khumal-4	23	3	Yes	No
K_id6002100	IR64_x_Sugandha-1	38	0	Yes	No
K_id6002100	IR64_x_Sunaulo_Sugandha	20	0	Yes	No
K_id6002100	IR71033_x_Anamol_Masuli	1	0	Yes	No
K_id6002100	IR71033_x_Khumal-4	9	2	Yes	No
K_id6002100	IR71033_x_Sugandha-1	42	0	Yes	No

ID	Cross tested	Successful assays	Failed assays	Marker-cross combination validated?	Met filtering criteria?
K_id6002100	IR71033_x_Sunaulo_Sugandha	29	1	Yes	No
K_id6002100	IRBB-60_x_Sunaulo_Sugandha	16	0	Yes	No
K_id6002100	Khumal-4_x_IRBB-60	7	0	Yes	No
K_id6002100	Loktantra_x_Sunaulo_Sugandha	83	1	Yes	No
K_id6004862	IR64_x_Anamol_Masuli	2	0	Yes	No
K_id6004862	IR65482_x_Anamol_Masuli	17	0	Yes	No
K_id6004862	IR71033_x_Anamol_Masuli	1	0	Yes	No
K_id6006336	IR64_x_Khumal-4	23	3	Yes	Yes
K_id6006336	IR64_x_Sugandha-1	38	0	Yes	Yes
K_id6006336	IR64_x_Sunaulo_Sugandha	20	0	Yes	Yes
K_id6006336	IR65482_x_Khumal-4	12	6	Yes	Yes
K_id6006336	IR65482_x_Sugandha-1	18	1	Yes	Yes
K_id6006336	IR65482_x_Sunaulo_Sugandha	23	0	Yes	Yes
K_id6006336	IR71033_x_Khumal-4	9	2	Yes	Yes
K_id6006336	IR71033_x_Sugandha-1	42	0	Yes	Yes
K_id6006336	IR71033_x_Sunaulo_Sugandha	29	1	Yes	Yes
K_id6006336	Loktantra_x_Sunaulo_Sugandha	83	1	Yes	Yes
K_id6011324	IR64_x_Sunaulo_Sugandha	20	0	Yes	No
K_id6011324	IR65482_x_Anamol_Masuli	19	0	Yes	No
K_id6011324	IR65482_x_Khumal-4	12	6	Yes	No
K_id6011324	IR65482_x_Sugandha-1	19	0	Yes	No
K_id6011324	IR71033_x_Sunaulo_Sugandha	29	1	Yes	No
K_id6011324	IRBB-60_x_Sunaulo_Sugandha	16	0	Yes	No
K_id6016490	IR64_x_Khumal-4	24	2	Yes	No
K_id6016490	IR64_x_Sugandha-1	38	0	Yes	No
K_id6016490	IR64_x_Sunaulo_Sugandha	20	0	Yes	No
K_id6016490	IR65482_x_Anamol_Masuli	19	0	Yes	No
K_id6016490	IR71033_x_Khumal-4	9	2	Yes	No
K_id6016490	IR71033_x_Sugandha-1	42	0	Yes	No
K_id6016490	IR71033_x_Sunaulo_Sugandha	29	1	Yes	No
K_id6016490	IRBB-60_x_Sunaulo_Sugandha	16	0	Yes	No
K_id6016490	Khumal-4_x_IRBB-60	7	0	Yes	No
K_id7000304	IR64_x_Anamol_Masuli	2	0	Yes	Yes
K_id7000304	IR64_x_Khumal-4	3	0	Yes	Yes
K_id7000304	IR64_x_Sugandha-1	37	1	Yes	Yes
K_id7000304	IR64_x_Sunaulo_Sugandha	13	0	Yes	Yes
K_id7000304	IR71033_x_Anamol_Masuli	1	0	Yes	Yes
K_id7000304	IR71033_x_Khumal-4	2	0	Yes	Yes
K_id7000304	IR71033_x_Sugandha-1	41	1	Yes	Yes
K_id7000304	IR71033_x_Sunaulo_Sugandha	26	0	Yes	Yes
K_id7000304	IRBB-60_x_Sunaulo_Sugandha	16	0	Yes	Yes
K_id7000304	IR64_x_Khumal-4	0	23	No	Yes
K_id7000304	IR64_x_Sunaulo_Sugandha	0	7	No	Yes

ID	Cross tested	Successful assays	Failed assays	Marker-cross combination validated?	Met filtering criteria?
K_id7000304	IR71033_x_Khumal-4	0	9	No	Yes
K_id7000304	IR71033_x_Sunaulo_Sugandha	0	4	No	Yes
K_id7000304	Khumal-4_x_IRBB-60	0	7	No	Yes
K_id7005611	IR64_x_Anamol_Masuli	2	0	Yes	Yes
K_id7005611	IR64_x_Khumal-4	3	0	Yes	Yes
K_id7005611	IR64_x_Sugandha-1	38	0	Yes	Yes
K_id7005611	IR64_x_Sunaulo_Sugandha	13	0	Yes	Yes
K_id7005611	IR65482_x_Anamol_Masuli	17	0	Yes	Yes
K_id7005611	IR65482_x_Sugandha-1	19	0	Yes	Yes
K_id7005611	IR65482_x_Sunaulo_Sugandha	17	0	Yes	Yes
K_id7005611	IRBB-60_x_Sunaulo_Sugandha	16	0	Yes	Yes
K_id9007356	IR65482_x_Anamol_Masuli	19	0	Yes	No
K_id9007356	IR65482_x_Khumal-4	15	3	Yes	No
K_id9007356	IR65482_x_Sugandha-1	17	2	Yes	No
K_id9007356	IR65482_x_Sunaulo_Sugandha	23	0	Yes	No
K_id9007356	IR71033_x_Anamol_Masuli	1	0	Yes	No
K_id9007356	IR71033_x_Khumal-4	9	2	Yes	No
K_id9007356	IR71033_x_Sugandha-1	42	0	Yes	No
K_id9007356	IR71033_x_Sunaulo_Sugandha	28	2	Yes	No
ud3001370	IR64_x_Khumal-4	0	3	No	No
ud3001370	IR65482_x_Anamol_Masuli	0	17	No	No
ud3001370	IR65482_x_Sugandha-1	0	19	No	No
ud3001370	IR65482_x_Sunaulo_Sugandha	0	17	No	No
ud3001370	IR71033_x_Khumal-4	0	2	No	No
ud4000418	IR64_x_Sunaulo_Sugandha	19	1	Yes	Yes
ud4000418	IR65482_x_Sunaulo_Sugandha	23	0	Yes	Yes
ud4000418	IR71033_x_Sunaulo_Sugandha	30	0	Yes	Yes
ud4000418	Khumal-4_x_IRBB-60	6	1	Yes	Yes
ud4000418	Loktantra_x_Sunaulo_Sugandha	83	1	Yes	Yes
ud7000203	IR64_x_Anamol_Masuli	2	0	Yes	Yes
ud7000203	IR64_x_Khumal-4	24	2	Yes	Yes
ud7000203	IR64_x_Sugandha-1	38	0	Yes	Yes
ud7000203	IR65482_x_Sunaulo_Sugandha	22	1	Yes	Yes
ud7000203	IR71033_x_Sunaulo_Sugandha	30	0	Yes	Yes
ud7000203	Khumal-4_x_IRBB-60	7	0	Yes	Yes
ud7000203	Loktantra_x_Sunaulo_Sugandha	83	1	Yes	Yes
wd6000320	IR64_x_Anamol_Masuli	2	0	Yes	Yes
wd6000320	IR64_x_Khumal-4	3	0	Yes	Yes
wd6000320	IR64_x_Sugandha-1	38	0	Yes	Yes
wd6000320	IR64_x_Sunaulo_Sugandha	13	0	Yes	Yes
wd8000350	IR64_x_Anamol_Masuli	2	0	Yes	Yes
wd8000350	IR65482_x_Anamol_Masuli	17	2	Yes	Yes
wd8000350	IR71033_x_Anamol_Masuli	1	0	Yes	Yes

ID	Cross tested	Successful assays	Failed assays	Marker-cross combination validated?	Met filtering criteria?
wd8003431	IR64_x_Anamol_Masuli	2	0	Yes	Yes
wd8003431	IR64_x_Khumal-4	3	0	Yes	Yes
wd8003431	IR65482_x_Anamol_Masuli	17	0	Yes	Yes
wd8003431	IR71033_x_Anamol_Masuli	1	0	Yes	Yes
wd8003431	IR71033_x_Khumal-4	2	0	Yes	Yes
