# Supplementary material for: Accelerating public sector rice breeding with high-density KASP markers derived from whole genome sequencing of indica rice
Source: Mol Breed. 2018 Mar 7;38(4):38. doi: 10.1007/s11032-018-0777-2 (PMC5842261; doi:10.1007/s11032-018-0777-2)
Supplement: Supplementary file 4 — Table S7 Sequences for new validated KASP assays available from LGC genomics. (PDF 50 kb) [file 11032_2018_777_MOESM4_ESM.pdf]

Table S7 Sequences for new validated KASP assays available from LGC genomics.

ID	Sequence
novel_01	AGGACTGCGCGCTATGGAAGCGACAYCTTCTGACCCTTACCGTCCAAATG[Y]TGATGGCAATCTACGTTGTCAGCAAGCAGTGGMGAGGTGACAAGTGGCTG
novel_02	TCTAACCCGTAGATCTTCACGTGATATGCCTTTATTAGTTTTAATTCTGT[R]GGCTTGGTTTAATCTCTGCGTTTAGTTAGTTTTGTGGGCTGGGTTTAATC
novel_03	TGATTTATTCAGTCACATTAGCGACCGCCCTTCTTCTAGAGGAAGCTCGACTACATCAACA[R]TTGGAAGTGACTTCCTTGACATACCTAGTKTACGGGGTCCAAAATCAAGTCATTCATCAG
novel_04	CAATATCTAAACTATTTGCTTCTCTAGAAGAGAAGCACCTGGAGCCCAGC[Y]AAACAGTTTCAGCTCCACCTAAAATAGGAGCGRAGTTGGGTGGAACACTC
novel_05	GCCTTGCACCCTTCGCTTGAGACGCCTCTGGCCCGGCAAGGCACTCGTAM[R]ACTCTTCGCCTCACGCCGTTCTTGCTCCATAGCTTTCGCCACGGGGTTCG
novel_06	GTACAGATGTTAGTGTTTTKGAAAATCCAGTGAAACATAAGTTGCTAACC[R]GGAAGCTTCCCCTCAGGTGGACACTGCTCAGGACCTGTGACATCAATCCG
novel_07	AGTAATGGTTCTCTTTGGCGTAAAATAAAGAGAAATCTGGCGTCTCGTCGTCTCCTCCGCTCC[K]CCTCTCCTTGCGCCAACAAGCCGACTCGCGATCTCCATCGGGCAAGAAGAGCAGAGCAGAGC
novel_08	GATGGTCTCCAGCGGCACCCTCAGCCCGGAGCTYGCCATTCAAGTTCTTG[W]SCAGTTTGATAAGGTATCTRCTTTACCATATCTCCTTCCACCTATTACTA
novel_09	TTGTGTTTTTTCCTGGCAAGTATGGTTTTTAATCTTCCAAATGGTTGGATGGG[Y]GCTGCAGCACTGCAACCATCAATGAATCGCTTGTCTAGCTGGTGGTTCAAAA
novel_10	TGAATCGCAGTCCTCCTGTGGTTTCGAGAAAAAAAAACTCACACATTGCAATATTGCCCATATTAAAACA[Y]TGATTTTTCCCCATAAACCAAAACAAGAAATAAAGTAACTAAGTGACCCTGGTGTGTACATGTTTATTA
novel_11	GCCTTGGAGGCCTGAGTGATGGATCCTATATATTCGTGAGGGGTGTGACC[R]GCGGAGRCATTGCATCACGTGGGAAACCCTAGCTGTCACTTCCCTCCCCG
novel_12	ATATGGGCWCCRACTTTCCAATGTGGATGGAAGATGGTGTGACACTGCAA[W]ATATTGTTAAACTCAGTTTGAGAGGCTCTGTAATGTGTGTCAAACTCCCA
novel_13	TACATCATCTTTCTGAGTACCTGATAGAAGAGCTCAAGGAGAAGAGGTACTTTGTT[R]TTCTAGATGATCTATGGATTTTACATGATTGGAATTGGATAAATGAAATTGCATTTCCTAAGAACAATAA
novel_14	CTTACCTTGGGGAGAAGCTAGTATTCAAAACGGGAGCATTCCCAAATCTTAGAACACTT[Y]GKATTTAYGAATTGGATCAGCTAAGAGARATNAGATTTGAGGACRGCAGCTC
novel_15	GGACAGCGACGTGCGGGTGTTGACTGTTGAGCCCCGAGGCCTACCTCGGCGAGAAGGAGAAGGT[Y]ACCACTCACCCGCGCTGTCGTMAACATGCYGGCCTACTTCAACAATGCGCAGCGCGTGGC
novel_16	GGAATTTCATTAGAGGAGAGAAAAATAGTTCAGAGAATACAGGWGAAGACATC[R]AAGGAGAAGAAGGRAGAAACCCGARAAAAACAAACGGAATCCGAAAAAGAAAAGACAATTAGGGAGGTTG
novel_17	TACTTGAGATACTTCTGTCAGTAACAACTGCTAGCTTTGTTTCTTGCAAACA[CACAATGGAAG/-]CACCAGTTTGATAAACAAAAGCTTTAACTGCATCCTGCTTTGCTTGGGCATTAAGCCCTCTTACATT
novel_18	TTAGCAACTTCATAAGAKTAGAGATGGGCAAGGGGCCCGTTTTCTGTTTC[Y]GCGCGGTGAATTCACCTGTTACGAGACGGGAATGGTTTGAATTGGTTCTC
novel_19	GAGGGGACAGTATTTTAGATCTTTTTCTTGGGTA[R]GGTTATAGTGCTTTGATGTACTCTTCCAACAGTTCAA
novel_20	TTTTTATGTGAAAGCTTCATCGTCAACACCTACGCAGGGCCGGCCCTGGGGCTA[Y]GTGTAACATCCTGAAAATTCGACCGACAAAAAGCTAAACTCTAAAAATATGGATTTTCAAAAACTTTT
novel_21	TCGACCAATGTATAATTTCCGGAGTCTACAAGGAGCAAGGGTCCACAAATGTAGTGAGAAAT[Y]GTCTTCCTCGTCATGTCCATGGATATCAAGAGAAAGAGACTCAAGGTAGCATGTGCCTA
novel_22	GTTATTGGCAGCTTCCCTCAATTGAGGAGGGCACCTTTCATCAGAATT[R]AAAATTCTTCTGAAAAACAATCTTTTGGAGTCAGTAGCACAAAGAGCTTTCATTTCATATA
novel_23	ACCAATGCACACTCAATTATTCTCCAAGTTTGTGCATCCCATA[W]ATCATCAATGACAAKAAAATATCTGTTTCACATGACTTTTAATTATCAACCAAGGATGTATTGATAC
novel_24	CGGGCATCGTGCTCAACGCCGCCACCTCCTTCTGGCTCCCCGTCCCTCCC[R]CCGCCGTCTTCGACTTCCTCCGYGACGAGACCTCTCGCAGCGAGGTGCCC
novel_25	TCCAATGCGACCGGTATCACATTAGATGGCTCCACCTCYGTCTTGGATAG[Y]GGCTTCGTCATGGCGAAGTCRAAGTTGAGTTGACGATGAACAGATCTCCT
novel_26	TTCAAATAACTTTTYGGATAGTAAATGATTTCAAATTGGAAAGTTGTCAA[Y]AACAAAGTTGTATAACTTATGAAGATCTACAACTTCTGTTTTGGTCATTT
novel_27	CACGGCCATGGGTGTGCACATTTGATTGGTGCGCAACAACAAGTGTATAT[TG/-]TTTGTGTGCTTCGTTAGTTGGCAGGTCCTAGTCACTAAATCACTATTGGA
novel_28	CTAGTCACTAAATCACTATTGGATTGGTACTAGCTACTTTTGTGCCTTGA[Y]GATGGGACTGGATTACTAGCCTTTTGGTTGCCTTTGTGGTATTCCGTTGT
novel_29	AAAAGATGTCCTGTATTATTTTGAGCAGATCTTGTACTGGTTGATCGGCT[Y]GCATGAAAATATTGTTGAGGATTATAATGCCATGCCAACTGAGTAAAGAA
novel_30	AATTAGTAACATGTTGGGTGATCCATCAATCGACTTCTATCGTCAGAAAA[K]AATTGCAGAGTAGTTCGTGTGCAATCAAGTGACTAAGCTCAAATCGAACA
novel_31	ACTTAGACCAGCAGGTGGTGCACTGACTTGGCTATTTTCTTGACGTTCTG[S]CCTATTCACCTTTGTACTAACATCTTCAAAGAACCGAGCACAGAAAGTAA
novel_32	ATGCATGGAACTGATTCTATGCATTTATTGCTCCAGATTGTTCCAACAAC[Y]ACTTAATAAAATTTTGACMGGAGAATACATAAGTGATTAATTAGTCGTTA
novel_33	CGATTTAAATCGATATGAGGATAACTACCCATCTCGTGGGCAAAACGGAA[G/-]GTTTCCAAGACAAACCTACAAAGAGGTATCGTACTCTCGCAGCAGCGGCG
novel_34	AGAACCAAATCTGTGATGTACTTGCACATAGGGAAACTTTATGCAACATCTATAT[W]CGGACSTCTGCAACATGCATCATCTTCTGGTGCATCCCATTACCATTCCATTGAATTTTGAAGTCTTT
novel_35	CTGTGCATGTTGTTCAGTCTTAGATAAGTGAGGTTTGACAAGTGTGATGCGAGCATCCCCA[R]TGGATCTTCCCCAAGATTACACSAACTTAGAGCTAAGTACTTGAGATGTGTAGTGTGGC
novel_36	CTCCTRTGCTTTATCCGTCTGGTRAGGAACATTGCAATGAGAACAATGCA[W]ATAAAGCCTGTTGCGCTACCAACTCCTATACCRATGCTTAAACCTGAAAG
novel_37	GCGAGATCGCCTTCCTGAAAGATGAGCTGAGCAGCATGAACGCGCTCCTCGAGAGGCTGGCCGACACGGAGGC[R]GCGCTCGATCCGCAGACGAAGGAGTGGAGGAGCCAGGTGAGGGAGATGAGCTATGACATTGAGGACTG
novel_38	ATTCTGCAGCGTCCCATTAAAACAATAGGACGCCACCGATCTAAAGCGTC[Y]GGCCTTCTCACGGACGCGATATTCTCATTGYGTCCATAGCCCACGGGCCA
novel_39	AGCCGAATTGAACACGTAGTCTCCTTAGTATCTCACCTATCAAGCAGTTC[S]TGAAGATATGATGGATGCTATTCTTTTGACTTCACCTCTCATAAAGTTGT
